# Genomic data imputation with variational auto-encoders

**DOI:** 10.1093/gigascience/giaa082

**Published:** 2020-08-06

**Authors:** Yeping Lina Qiu, Hong Zheng, Olivier Gevaert

**Affiliations:** Stanford Center for Biomedical Informatics Research, Department of Medicine, Stanford University, Stanford, CA 94305, USA; Department of Electrical Engineering, Stanford University, Stanford, CA 94305, USA; Stanford Center for Biomedical Informatics Research, Department of Medicine, Stanford University, Stanford, CA 94305, USA; Stanford Center for Biomedical Informatics Research, Department of Medicine, Stanford University, Stanford, CA 94305, USA; Department of Biomedical Data Science, Stanford University, Stanford, CA 94305, USA

**Keywords:** imputation, variational auto-encoder, deep learning

## Abstract

**Background:**

As missing values are frequently present in genomic data, practical methods to handle missing data are necessary for downstream analyses that require complete data sets. State-of-the-art imputation techniques, including methods based on singular value decomposition and K-nearest neighbors, can be computationally expensive for large data sets and it is difficult to modify these algorithms to handle certain cases not missing at random.

**Results:**

In this work, we use a deep-learning framework based on the variational auto-encoder (VAE) for genomic missing value imputation and demonstrate its effectiveness in transcriptome and methylome data analysis. We show that in the vast majority of our testing scenarios, VAE achieves similar or better performances than the most widely used imputation standards, while having a computational advantage at evaluation time. When dealing with data missing not at random (e.g., few values are missing), we develop simple yet effective methodologies to leverage the prior knowledge about missing data. Furthermore, we investigate the effect of varying latent space regularization strength in VAE on the imputation performances and, in this context, show why VAE has a better imputation capacity compared to a regular deterministic auto-encoder.

**Conclusions:**

We describe a deep learning imputation framework for transcriptome and methylome data using a VAE and show that it can be a preferable alternative to traditional methods for data imputation, especially in the setting of large-scale data and certain missing-not-at-random scenarios.

## Introduction

The massive and diverse data sets in genomics have provided researchers with a rich resource to study the molecular basis of diseases. The profiling of gene expression and DNA methylation have enabled the identification of cancer driver genes or biomarkers [1–6]. Many such studies on cancer genomics require complete data sets [7]. However, missing values are frequently present in these data due to various reasons, including low resolution, missing probes, and artifacts [8, 9]. Therefore, practical methods to handle missing data in genomic data sets are needed for effective downstream analyses.

One way to complete the data matrices is to ignore missing values by removing the entire feature if any of the samples has a missing value in that feature, but this is usually not a good strategy, as the feature may contain useful information for other samples. The most preferable way to handle missing data is to impute their values in the pre-processing step. Many approaches have been proposed for this purpose [10], including replacement using average values, estimation using the weighted K-nearest neighbor (KNN) method [11, 12], and estimation using singular value decomposition (SVD)–based methods [11]. KNN and SVD are 2 techniques that have been commonly used as benchmarks against new developments [13, 14]. KNN imputes the missing value of a feature in a given sample with the weighted average of the feature values in a number of similar samples, as calculated by some distance measure. SVD attempts to estimate data structure from the entire input, including the samples with missing values, and fill in the missing values iteratively according to the global structure. For this reason, SVD is inefficient on large matrices in practice, since new decompositions have to be estimated for each missing sample, which is a very time-consuming process. However, SVD serves as an important benchmarking method to determine how well other, faster methods perform compare to SVD.

In recent years, a branch of machine learning which emerged based on big data and deep artificial neural network architectures, usually referred to as deep learning, has advanced rapidly and shown great potential for applications in bioinformatics [15]. Deep learning has been applied in areas including genomics studies [16–18], biomedical imaging [19], and biomedical signal processing [20]. Auto-encoders (AE) operate on a deep learning–based model that forms the basis of various frameworks for missing value imputation, and AEs have shown promising results for genomic data, imaging data, and industrial data applications [21–26]. However, a simple AE without regularization is rarely ranked among the competitors for data imputation [27, 28]. When a simple AE only focuses on creating output close to the input without any constraints, the model may overfit on the training data instead of learning the latent structure, such as dependencies and regularities characteristic of the data distribution [22], which makes it unlikely to impute well when given new samples. A denoising auto-encoder (DAE) is a type of auto-encoder that specifically uses noise corruption to the input to create robust latent features [22]. DAE has been extensively used in the application of data imputation [23, 27]. The corrupting noise introduced in the DAE can be in many different forms, such as masking noise, Gaussian noise, and salt-and-pepper noise [29].

Variational auto-encoders (VAE) are probabilistic auto-encoders that have wide applications in image and text generation [30–32]. VAE learns the distributions of latent space variables that make the model generate output similar to the input. VAE has primarily been used as a powerful generative tool, having the ability to produce realistic fake contents in images, sound signal, or texts that highly resemble the real-life contents that they learn from. The generative power is made possible by regularizing the latent space [32]. Constraining the latent space distributions to be close to a standard Gaussian helps to achieve a smooth latent space where 2 close points in the latent space should lead to similar reconstructions, and any point sampled from the latent space should give a meaningful reconstruction [33]. VAE has been applied in genomic contexts, such as latent space learning of gene expression data [34]. In addition, recent works have applied VAE on single-cell RNA sequencing data for clustering, batch correction, and differential expression analysis [35, 36]. However, VAE has not been extensively studied for genomic data imputation for bulk RNA expression and DNA methylation data, while large amounts of retrospective genomic and epigenomic data are available through databases like the Gene Expression Omnibus (GEO) [37] and the Short Read Archive (SRA) [38].

Here, we examine the VAE mechanism and its application to genomic missing value imputation with bulk transcriptome and methylome data. We show that for both missing completely at random (MCAR) and missing not at random (MNAR) cases in transcriptome data and methylome data, VAE achieves similar or better performances than the de facto standards, and thus is a strong alternative to traditional methods for data imputation [39]. We demonstrate that in a MNAR scenario where the missing data distribution is not the same as the seen data, a shift correction method can be implemented to improve VAE's extrapolation performance. Furthermore, we investigate the effect of latent space regularization on imputation with a generalization of the variational auto-encoder: $\beta $-VAE [40]. In the context of $\beta $-VAE results, we provide insights on why VAE can achieve good imputation performance compared to a regular, deterministic AE.

## Materials and Methods

### Data sets

We use 2 data sets to perform data imputation: pan-cancer RNA sequencing data from The Cancer Genome Atlas (TCGA) data sets [2, 41, 42] and DNA methylation data [43–46]. Both data sets contain only numeric values. The RNA sequencing data is expressed in reads per kilobase of transcript, per million mapped reads, which is a normalized unit of transcript expression. The DNA methylation data is obtained from bisulfite sequencing, and it contains the numeric values of the methylation level at each 5'—C—phosphate—G—3' (CpG) site. The RNA sequencing data has a feature dimension of 20,531 genes. There are 15% of the genes containing more or less NA values, while the remaining 85% of the genes are complete. Within the 15% of the genes who have missing values, on average 8.5% of the values are missing. The NA values are introduced in the Synapse pre-processing pipeline, where genes with mostly 0 reads or with residual batch effects after batch correction were removed from the adjusted samples and replaced with NAs. In order to have a ground truth to evaluate the missing value imputation frameworks, we remove the 15% of genes with NA values in our pre-processing, which results in a feature dimension of 17,176 genes. We then normalize the data by log transformation and z-score transformation. We use 667 glioma patient samples, including those with glioblastoma (GBM) and low-grade glioma (LGG), to train and test the missing value imputation framework. In pre-processing the DNA methylation data, we remove the NA values, and normalize the data by negative log transformation and z-score transformation. We use the smallest chromosome subset (Chromosome 22) so that the resulting data dimension is not prohibitive for benchmarking different computation methods. The resulting data has 21,220 CpG sites and 206 samples.

### Missing data simulations

Each data set is split into 80% for training and 20% for hold-out testing. The training data set is further split 80/20%, where 20% is the validation data set for hyper-parameter tuning. After hyper-parameters are selected, the entire training set is used for training. The sample split for the RNA sequencing data set is stratified by the glioma subtypes (LGG versus GBM), and the split is random for the DNA methylation data since the samples are homogenous. The training data is a complete data set without missing values. Missing values are introduced to the testing data in 2 forms: MCAR and MNAR (Table 1) [47].

**Table 1:** Simulation experiments on RNA sequencing data and DNA methylation data.

Data	Missing type	Missing scenario
RNA sequencing data	MCAR	5% completely random missing
		10% completely random missing
		30% completely random missing
	MNAR	50% random missing in genes with the highest 10% guanine-cytosine content (GC) content
		5% genes are entirely missing
		50% random missing in genes with the lowest 10% expression level
DNA methylation data	MCAR	5% completely random missing
		10% completely random missing
		30% completely random missing
	MNAR	5% CpG sites are entirely missing
		50% random missing in CpG sites with coverage lower than 6 reads

In the MCAR cases, we randomly mask a number of elements in each row by replacing the original values with NAs. To test a range of missing severity, we make the number of masked elements amount to 5%, 10%, and 30% of the total number of elements, respectively.

Each of the MNAR simulations is motivated by a different real-world condition specific to either gene expression data or methylation data. For the gene expression data, we simulate 3 MNAR scenarios, each of which has 5% of the total data values missing. In the first scenario, the masked values are concentrated at certain genes. Such genes are selected based on their GC content, which is the percentage of nitrogenous bases on a RNA fragment that are either guanine (G) or cytosine (C). GC content that is too high or too low influences RNA sequencing coverage, and potentially results in missing values from these genes [48]. We select genes with GC content at the highest 10% and randomly mask half of these values. In the second simulation case, certain genes are masked entirely. In some pre-processing pipelines of RNA sequencing data, genes with residual batch effects after batch correction are replaced with NAs in the adjusted samples. Such pre-processing may give rise to the MNAR case where some genes are entirely missing in some samples. We randomly select 5% of the genes and mask all values from these genes in the testing data; as a result, the corrupted data miss all values for specific genes. The third scenario is based on gene expression level. When the RNA sequencing depth is relatively low, it is relatively easy to miss genes that have low expression levels, because the reads generated from those genes are too few to be captured during sequencing [49]. Therefore, we consider a possible scenario where lowly expressed genes are prone to be missing. In the testing data, we first choose gene expression values at the lowest 10% quantile, and then randomly mask half of these values.

For the DNA methylation data, we simulate 2 MNAR scenarios. The first scenario is completely missing certain CpG sites, which is similar to the second MNAR case in gene expression data, where we select 5% of the features and mask them entirely in the testing data. In the second case, we mask CpG sites that have less coverage than a certain threshold. Some CpG sites may have very few reads mapped to them, which undermines the confidence in the measurement of methylation level. Thus, we choose an arbitrary coverage threshold of 6 reads for the methylation status of a CpG site to be confidently determined. Methylation levels of CpGs with fewer than 6 reads mapped to them are treated as missing values in the analysis here.

For each simulation scenario described above, we create 10 random trials to measure the average imputation performance. The uncorrupted testing data is used to compute the imputation root mean squared error (RMSE).

### Variational auto-encoder

An AE is an unsupervised deep neural network that is trained to reconstruct an input *X* by learning a function ${h_{w,\ b}}( X ) \approx X$. This is done by minimizing the loss function between the input *X* and the network's output $X^{\prime}$:$\ L( {X,\ X^{\prime}} )$. The most common loss function is the RMSE:

(1) $$\begin{equation*}
L\ \left( {X,X^{\prime}} \right) = \sqrt {{{\left| {\left| {X - X^{\prime}} \right|} \right|}^2}} \
\end{equation*}$$

An auto-encoder consists of an encoder and a decoder. The encoder transforms the input to a latent representation, often such that the latent representation is in a much smaller dimension than the input [50]. The decoder then maps the latent embedding to the reconstruction of *X*. An auto-encoder is often used as a dimensional reduction technique to learn useful representations of data [51].

While in a regular auto-encoder the latent space is encoded and then decoded deterministically—that is, there is no probabilistic modeling of the latent space—a VAE learns a probability distribution in the latent space. VAE is often used as a generative model by sampling from the learned latent space distribution and generating new samples that are similar in nature to the original data [32]. The assumption of VAE is that in the distribution of data X, $P( X )$ is related to the distribution of the latent variable z, $P( z )$ by

(2) $$\begin{equation*}
{P_\theta }\ \left( X \right) = \smallint {P_\theta }\left( {X{\rm{|}}z} \right)P\left( z \right)dz\
\end{equation*}$$

Here, ${P_\theta }( X )$, also known as the marginal likelihood, is the probability of each data point in X under the entire generative process, parametrized by $\theta $. The model aims to maximize ${P_\theta }( X )$ by optimizing the parameter $\theta $ so as to approximate the true distribution of data. In practice, ${P_\theta }(X|z)$ will be nearly 0 for most z, and it is therefore more practical to learn a distribution ${Q_\phi }(z|X)$, which gives rise to a z that is likely to produce X, and then compute $P( X )$ from ${E_{z\sim{Q_\phi }}}P( {X{\rm{|}}z} ).\ {P_\theta }$ (X) and ${E_{z\sim{Q_\phi }}}P( {X{\rm{|}}z} )$ can be shown to have the following relationship [32]:

(3) $$\begin{eqnarray*}
log{P_\theta }( X ) - D[{Q_\phi }(z|X)||{P_\theta }(z|X)] =&& {{E_{z\sim{Q_\phi }}}\ } [log{P_\theta }(X|z)] \nonumber \\
&& - D [{Q_\phi }(z|X)||P( z )]
\end{eqnarray*}$$

The left side of (3) is the quantity we want to maximize, $\log {P_\theta }( X )$, plus an error term, which is the Kullback-Liebler divergence between the approximated posterior distribution ${Q_\phi }( {z{\rm{|}}X} )$ and the true posterior distribution ${P_\theta }( {z{\rm{|}}X} )$. The Kullback-Liebler divergence is a measure of how 1 distribution is different from another, and is always non-negative. Thus, maximizing the log likelihood log $P( X )$ can be achieved by maximizing the evidence lower bound (ELBO):

(4) $$\begin{equation*}
{\textit ELBO}\ = \log {P_\theta }\left( X \right)\ - \mathcal{D}[{Q_\phi }(z|X)|{\rm{|}}{P_\theta }\left( {z{\rm{|}}X} \right){\rm{]}}
\end{equation*}$$

The right side of (3) is something we can optimize by a gradient descent algorithm. ${P_\theta }( {X{\rm{|}}z} )$ is modeled by the decoder network of the VAE parametrized by $\theta $, and ${Q_\phi }(z|X$) is modeled by the encoder network parametrized by $\phi $. For continuous value inputs, ${P_\theta }( {X{\rm{|}}z} )$ and ${Q_\phi }(z|X$) are most commonly assumed to be Gaussian distributions [33]. $P( z )$ is fixed prior to distribution and is assumed to be a standard multivariate normal distribution $\mathcal{N}( {0,\ I} )$. The first term,${E_{z\sim{Q_\phi }}}[log{P_\theta }(X|z)]$, is the expectation of the log probability of X given the encoder's output. Maximizing this term is equivalent to minimizing the reconstruction error of the AE. The second term, $D[{Q_\phi }(z|X)||P( z )]$, is the divergence between the approximated posterior distribution ${Q_\phi }(z|X)$ and the prior $P( z )$, and minimizing this term can be considered as adding a regularization term to prevent overfitting.

VAE is trained with the training data that follows a standard Gaussian distribution after z-score transformation. We impute missing values in the testing data with a trained VAE by an iterative process. Initially, the missing values are replaced with random values sampled from a standard Gaussian distribution. Then, the following sequence of steps are repeated until an empirically determined iteration threshold is reached: compute the latent variable z distribution given input X with the encoder; take the mean of latent variable distribution as the input to the decoder and compute the distribution of reconstructed data $\hat{X}$; take the mean of the reconstructed data distribution as the reconstructed values; replace the missing values with reconstructed values; and leave non-missing values unchanged. The testing data should be scaled by the model's training data mean and variance before the imputation iterations, and should be inverse scaled after imputation.

### VAE imputation with shift correction

Regular implementation of VAE has an underlying assumption that the training data follows the same distribution as testing data. Below, we will discuss how to modify this assumption to better impute MNAR scenarios.

Since the VAE learns the data distribution from the training data, the output of imputation also follows the learned distribution, which is similar to the training data. When the missing values are drawn from a different distribution than the training data, the imputation performance will drop due to the distribution shift. In the MNAR simulations where half of the lowest 10% of values are masked, the missing values are considered to be shifted from the original training data to a smaller mean.

The lowest-value-missing scenario represents a common type of missing values in biomedical data. When certain experimental conditions (e.g., low RNA sequencing depth) allow us to make assumptions that the majority of missing values are low-expression values, we essentially have prior knowledge that the distribution of missing values is shifted to the end of lower values. We can therefore use VAE with the shift-correction implementation. Recall that in (3), the underlying assumption is that the training data follows a Gaussian distribution X ∼ $\mathcal{N}( {\mu ,\ \sigma } )$, where *μ* and $\sigma $ are the outputs of the decoder network that represent the mean and variance, respectively, of the observed training data, as well as the missing data. When the lowest values are missing, the learnt distribution has a larger mean than the actual missing data, causing the reconstructed $\hat{X}$ to have larger values. To correct this, we modify the assumption of training data distribution to follow $\mathcal{N}( {\mu + \lambda \sigma ,\ \sigma } )$, where $\mu $ and $\sigma $ are the outputs of the decoder network that represent the mean and variance, respectively, of the missing data, and $\lambda $ is a hyperparameter. The mean of the observed training data is then shifted to $\mu + \lambda \sigma $. VAE with shift correction is recommended for use when certain experimental conditions warrant the assumption that missing values are concentrated on the lower end of the data distribution. However, when such assumptions are unknown or the pattern of missing data is more likely to be random, the standard VAE without shift correction is recommended for use.

To test the lowest 10% missing case, we simulate a 10% lowest-value-missing scenario on the validation data set, and select the shift correction parameter value that produces the smallest validation error. In reality, we may not know the actual ranges and amounts of low values missing in the testing data, and thus cannot simulate the situation on the validation data precisely. For a range of the lowest-value-missing scenarios where half of the lowest 5%, 10%, 20%, and 30% values are missing, we impute with a single $\lambda $, which is selected based on the lowest 10% missing case. We thereby determine whether it is possible to select $\lambda $ without precise knowledge of the missing scenario in the testing data.

### *β*–variational auto-encoder

*β*-VAE is a generalization of the VAE with a focus to discover interpretable factorized latent factors [40]. A hyperparameter beta is introduced to the VAE loss to balance the reconstruction loss term with the regularization loss term. The loss of $\beta $-VAE is defined as:

(5) $$\begin{equation*}
{L_{\beta - {\rm{VAE}}}} = \ - {E_{z\sim{Q_\phi }}}\left[ {log{P_\theta }\left( {X{\rm{|}}z} \right)} \right] + \beta \mathcal{D}[{Q_\phi }(z|X)|{\rm{|}}P\left( z \right){\rm{]}}
\end{equation*}$$

where $\beta $ is a hyperparameter.

$\beta $-VAE ($\beta $ > 1) has been shown to perform better than VAE in certain image generation tasks and has attracted increasing research interest [52]. However, no prior work has investigated the effect of $\beta $ on imputation. Since VAE can be considered as a special case of $\beta $-VAE, we extend our study to $\beta $-VAE with a varying $\beta $ to further understand the effect of regularization on VAE imputation and to investigate the potential possibility of increasing its performance.

When $\beta $ is 1, it is the same as VAE. When $\beta $ > 1, a stronger regularization is enforced, and the resulting latent space is smoother and more disentangled, which is a preferred property in certain learning tasks because more disentangled latent space has greater encoding efficiency [40].

In comparison, when $\beta \ $ = 0, the regularization term is effectively removed. With the regularization term removed, the loss function only consists of the reconstruction loss term:

(6) $$\begin{equation*}
\ {L_{{\rm{VA}}{{\rm{E}}^{\prime}}}} = \ - {E_{z\sim{Q_\phi }}}\left[ {og{P_\theta }\left( {X{\rm{|}}z} \right)} \right]
\end{equation*}$$

which resembles the reconstruction loss function of a simple AE without any regularization. This can usually be expressed in the mean squared error between the input *X* and the reconstruction${\rm{\ }}X{\rm{^{\prime}}}$ [53]:

(7) $$\begin{equation*}
L\ \left( {X,X^{\prime}} \right) = \left\| {X - X^{\prime}} \right\|_2^2
\end{equation*}$$

However, the loss of VAE without the regularization term as shown in (6) has a key difference from the loss of a simple AE shown in (7). If (6) is viewed from a deterministic perspective, it is easy to distinguish the difference.

With the assumption that ${P_\theta }$ and ${Q_\phi }$are Gaussian distributions,

$$\begin{equation*}
{P_\theta }\ \left( {X{\rm{|}}z} \right)\sim\ N\ \left( {X{\rm{|}}{\mu _\theta }\left( z \right),\ diag\left( {{\sigma _\theta }\left( z \right)} \right)} \right),
\end{equation*}$$

$$\begin{equation*}
{Q_\phi }\ \left( {z{\rm{|}}X} \right)\sim\ N\ (z|{\mu _\phi }\left( X \right),\ diag\left( {{\sigma _\phi }\left( X \right)} \right))
\end{equation*}$$

the loss in (6) can be computed as the mean squared error between inputs and their mean reconstructions output by the decoder [33]:

(8) $$\begin{equation*}
\ {L_{{\rm{VA}}{{\rm{E}}^{\prime}}}} = \left\| {X - {\mu _\theta }\left( z \right)} \right\|_2^2
\end{equation*}$$

Unlike the deterministic reconstruction $X^{\prime}$ in (7), *z* in (8) is stochastic. However, the stochasticity of *z* can be relegated to a random variable that does not depend on $\phi ,$ so that we can view (8) from a deterministic perspective. Using the reparameterization trick [32], *z* can be represented by:

(9) $$\begin{equation*}
z\ = {\mu _\phi }\ \left( X \right) + {\sigma _\phi }\left( X \right)\ \odot \ \varepsilon ,\ \varepsilon \sim \mathcal{N}\left( {0,\ I} \right)
\end{equation*}$$

where $\odot$ is the element-wise product. Therefore, the input to the decoder can be considered as the output of encoder ${\mu _\phi }( X )$ corrupted by a random Gaussian noise $\varepsilon $ multiplied by ${\sigma _\phi }( X )$. Consequently, the loss in (8) can be considered as the loss of a deterministic AE, which has noise injected to the latent space. In contrast, noise is not present in the deterministic regular AE loss in (7).

We perform 3 random missing experiments (5%, 10%, and 30% missing) with $\beta $-VAE and vary the hyperparameter $\beta $ between 0, 1, 4, and 10 to evaluate how $\beta $ affects imputation accuracies. This will help us understand the VAE mechanism and how to use it in imputation.

### Model parameter and hyper-parameter tuning

Model parameter tuning and hyper-parameter tuning are conducted on the validation data set. The latent dimension is usually several magnitudes smaller than the input dimension in AE implementations, but there is no golden rule to determine its size. We test 3 latent dimension sizes: 50, 200, and 400. Furthermore, we test 2 architectures with 3 or 5 hidden layers. The hidden layers adjacent to the bottleneck layer have 10-fold size increases, and each adjacent layer outwards after that has a constant size increase factor. For example, for a 5–hidden layer VAE with a latent size of 50, the hidden layer dimensions are 3,000, 500, 50, 500, and 3,000, with input and output dimensions of 17,176; for a 3–hidden layer VAE with a latent size of 200, the hidden layer dimensions are 2,000, 200, and 2,000. We found that 5 hidden layers show better performance than 3 hidden layers, and that latent dimensions of 200 and 400 produce similar performances that are both better than 50. We therefore use a VAE with 5 hidden layers of dimensions of 6,000, 2,000, 200, 2,000, and 6,000 in our subsequent experiments. Supplementary Figure S1 shows the performance differences of the 6 different model architectures described above. The Rectified Linear Unit (ReLU) function is used as the activation function on the hidden layers.

We use the Adam optimizer and search for optimal learning rates on a grid of 1e-5, 5e-5, 1e-4, and 5e-4. A learning rate of 5e-5 is selected after the grid search. We find that model performance is not very sensitive to batch size, and use a batch size of 250 and training epochs of 250. The number of iterations to perform the iterative imputation is also determined empirically. The imputed values are found to converge very quickly, and results remain mostly stable after 2 or 3 iterations. We use 3 as the iteration threshold.

### Evaluation methods

To evaluate the VAE imputation framework, we compare it to the other most commonly used missing-value estimation methods: a KNN method and an iterative SVD-based method. We also construct a baseline using the mean-value imputation method. KNN selects K number of samples that are most similar to the target sample with a missing gene based on Euclidean distance, and which all have values present in that gene. Imputation is a weighted average of the values of that gene in those K samples. We chose K = 10 in our evaluations based on a study that reported that K values in the range of 10–25 gave the best imputation results [11]. Next, the SVD method decomposes the data matrix to a linear combination of eigengenes and corresponding coefficients. Genes are regressed against L most significant eigengenes, during which process the missing genes are not used [54]. The obtained coefficients are linearly multiplied by eigengenes to get a reconstruction with missing genes filled. This process is repeated until the total change in the matrix reaches a certain threshold. The reconstruction performance of SVD depends on the number of eigengenes selected for regression. We test a range of values and determine that the optimal performance is reached by full-rank reconstruction. Hence, we use full-rank SVD in our evaluations. The mean-value imputation method fills in the missing elements of each feature with the mean value of that feature across all non-missing samples.

We evaluate the RMSE of the imputed data and uncorrupted ground truth,

$$\begin{equation*}
{\textit RMSE}\ = \frac{{\mathop \sum \nolimits_{i = 1}^{{n_{missing}}} \sqrt {{{\left( {{x_i} - x_i^{\prime}} \right)}^2}} }}{{{n_{missing}}}}\
\end{equation*}$$

where ${x_i}$ is the ground truth of the masked value, and $x_i^{\prime}\ $is the reconstructed value for the masked value.

To further evaluate the imputation effect on a biomedical analysis, we compare the univariate correlation to clinical variables on the RNA sequencing data imputed by different methods. We conduct this analysis with the TCGA glioma cohort containing both LGG and GBM samples, and use 2 clinical variables: tumor histologic grade and survival time. The tumor grade and survival information for each brain tumor patient are publicly available [55]. The histologic grade variable in the TCGA brain tumor data contains 3 levels: Grade II, III, and IV, indicating increasing levels of tumor malignancy. We directly use the grade value as an ordinal variable of 3 levels, and calculate the Spearman correlation coefficient between each gene and the grade variable. The survival time is a continuous variable measured in months, and the vital status indicates whether the patient was dead or alive when the study concluded. With this information, we perform a Cox regression on each gene with respect to the survival outcome, and compute the univariate coefficient of each gene. A concordance index is computed between the coefficient obtained from the imputed data by each method and the coefficients obtained from the ground truth. A higher concordance index indicates better resemblance to the true data.

## Results

### RMSE of imputation on RNA sequencing data

**Figure 1:**
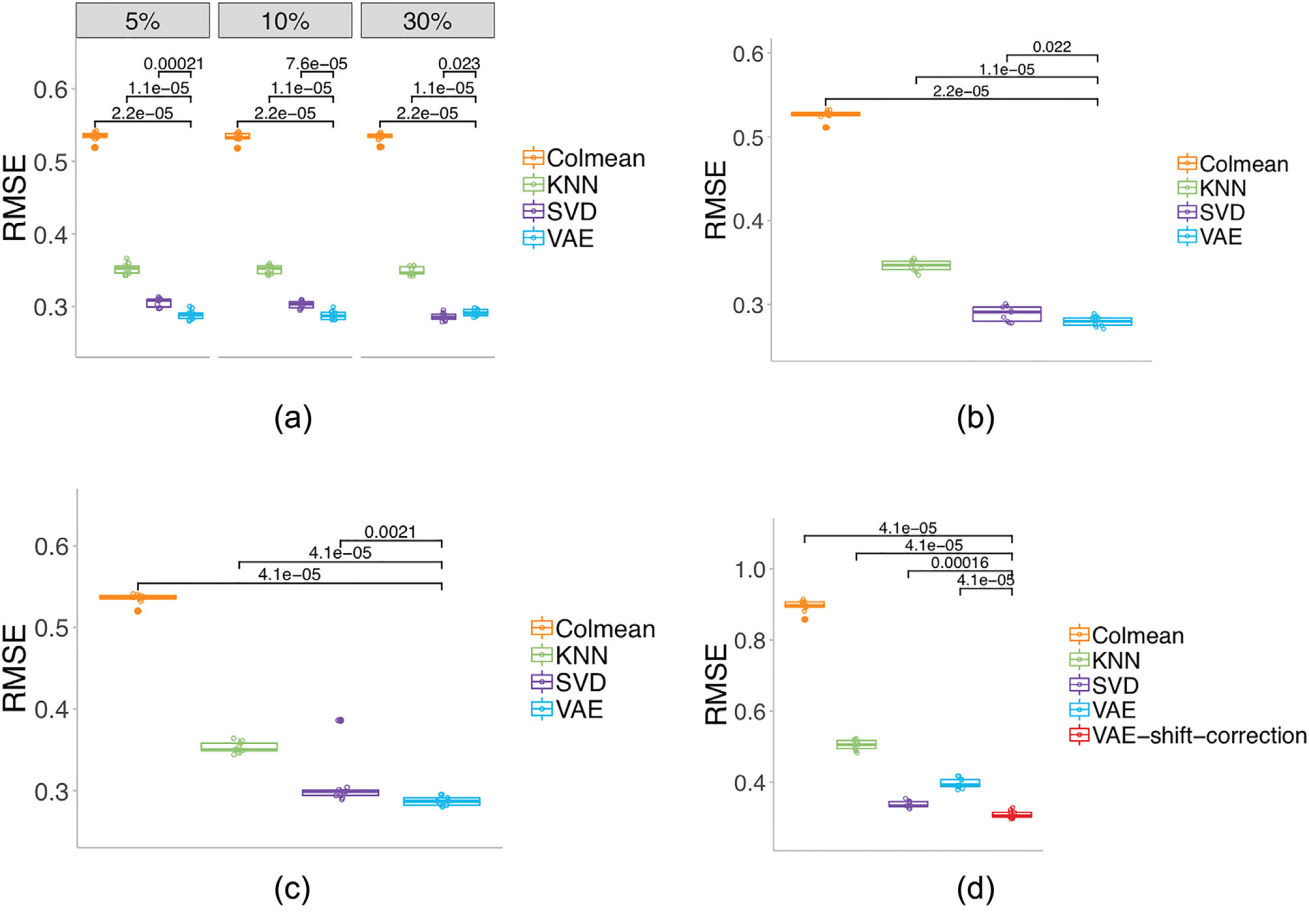
Imputation RMSE on the gene expression data for (**a**) MCAR cases of 5%, 10%, and 30%; (**b**) half of the highest 10% of GC content genes missing a case; (**c**) 5% of genes entirely missing a case; and (**d**) half of the lowest 10% of values missing a case. The numbers above bars show the Wilcoxon test significant scores between VAE or VAE with shift correction and other methods.

We inspect the RMSEs in different simulated missing scenarios by different imputation methods. The significant scores are calculated using the Wilcoxon test with the “ggsignif” package in R. First, we evaluate the MCAR cases at varying percentages: 5%, 10%, and 30% random elements in the testing data were masked, and models were compared on the reconstruction RMSE. VAE achieves better RMSEs than KNN in all tested missing scenarios, and reaches similar or better performances than SVD in most scenarios (Fig. 1a).

In the first MNAR simulation case, the masked values are confined to certain genes that have the highest 10% of GC content. Genes whose GC content is in the top 10% contain 50% random missing values in the testing data. VAE shows better reconstruction RMSE than KNN, and also achieves a slight advantage over SVD (Fig. 1b). In the second case, 5% of genes are masked entirely in the testing data. VAE again shows the lowest mean RMSE among competing methods (Fig. 1c). Each method may have different performance on different genes. Supplementary Table 1 provides insight on the imputation results for individual genes, showing the RMSEs obtained from each method for each individual gene from 1 experimental trial.

The final MNAR case is based on the gene expression values. The extreme values at the lowest 10% quantile are masked 50% randomly in the testing data. As a result, the observed values in the testing data shift its distribution from the training data, and result in a decreased performance of imputation. However, with shift-correction implementation, VAE again achieves similar or better imputation accuracy than other methods (Fig. 1d).

### The shift correction is robust to a range of low percentage-missing scenarios

**Figure 2:**
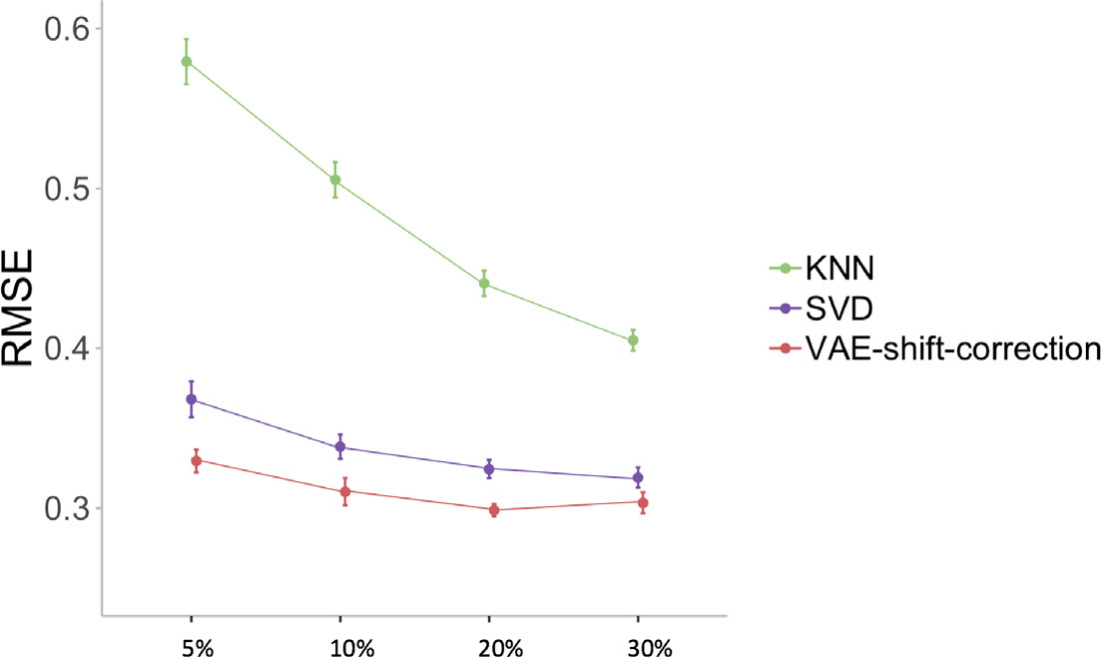
RMSE with 95% confidence intervals for simulations where half of the lowest 5%, 10%, 20%, and 30% of values are missing. VAE shift-correction results are achieved using a single $\lambda $, which is selected based on the lowest 10% of missing cases.

We further investigate the robustness of the shift correction parameter against a range of missing percentages on the lowest values. The shift correction parameter is selected based on a 10% lowest-value-missing scenario simulated on the validation data. We use the same selected parameter to test on a range of missing scenarios, where half of the lowest 5%, 10%, 20%, and 30% of values are missing. All methods show worse prediction errors for smaller thresholds of missing values, because smaller thresholds indicate that the missing values are concentrated to smaller values, leading to larger shifts in data distribution. We show that in these tested scenarios the shift-correction VAE consistently achieves better results than KNN and SVD with the same $\lambda $ (Fig. 2). Therefore, $\lambda $ selection does not need to exactly match the actual missing percentage, which is an advantage in real-world implementations.

### RMSE of imputation on DNA methylation data

**Figure 3:**
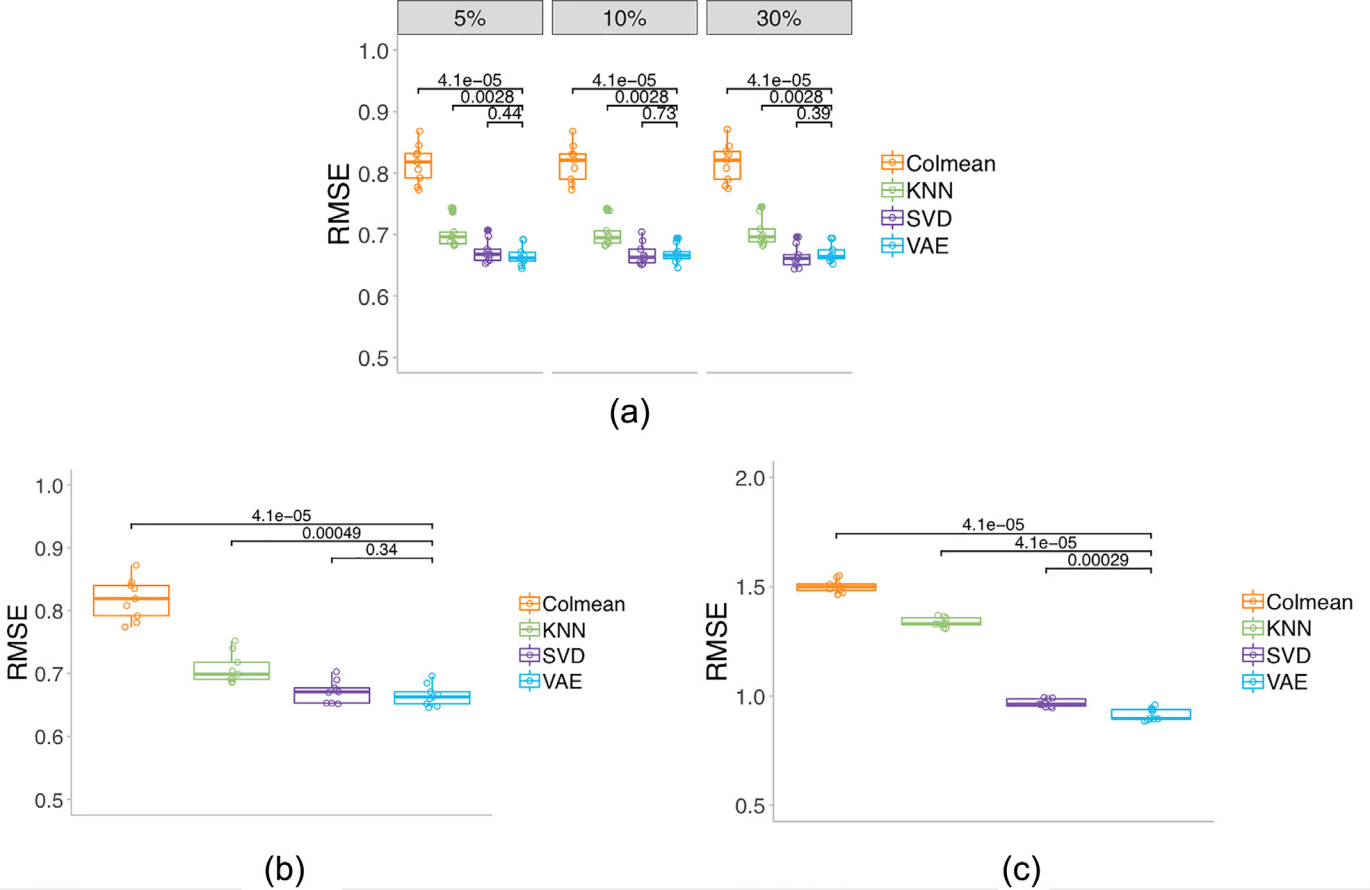
Imputation RMSE on the DNA methylation data for (**a**) MCAR cases of 5%, 10%, and 30%; (**b**) 5% of genes entirely missing; and (**c**) half of the coverage <6 CpG sites missing. The numbers above bars show the Wilcoxon test significant scores between VAE and other methods.

For the imputation on DNA methylation data, the KNN, SVD, and VAE methods show similar performance when compared to the gene expression data. These 3 methods also show better performance than imputing with a column mean. For MCAR and block missing cases, VAE has similar performance as SVD, followed by KNN (Fig. 3a, b). For the low-coverage missing case, VAE achieves a better RMSE than SVD and KNN (Fig. 3c).

### Correlation with clinical phenotypes

We investigate how closely the imputed data resembles the true data in terms of univariate correlation with respect to clinical variables. A higher concordance index between the correlation coefficients obtained from the imputed data and the coefficients obtained from the ground truth likely indicates the imputation method is better at preserving the original data's univariate properties.

The ground truth of univariate Spearman correlations to histologic grade ranges from -1 to 1, with 46% of the genes having an absolute correlation value of 0.3 or greater. The majority of ground truth Cox regression coefficients with respect to survival outcomes is in the range of -5 and 5, with 72% of the genes having an absolute coefficient value of 0.3 or greater.

**Figure 4:**
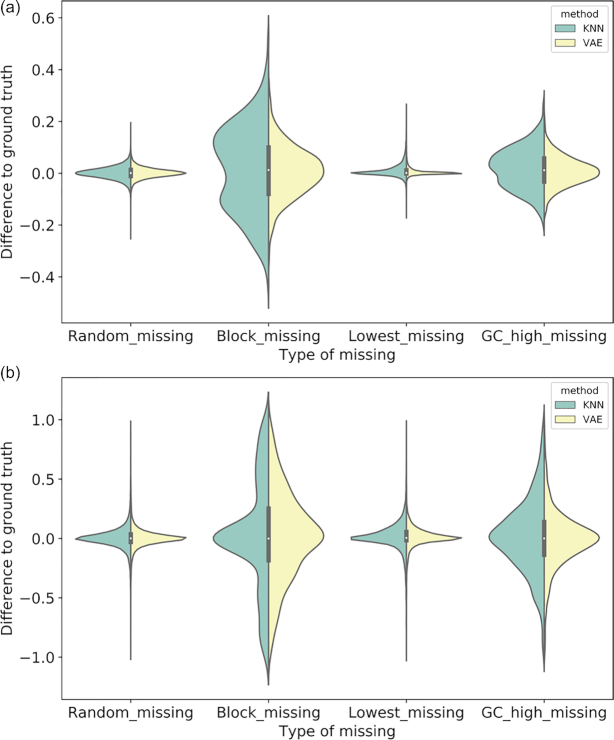
Pairwise difference between the coefficients obtained from the ground truth and the coefficients obtained from the imputed data by KNN and VAE: (**a**) Spearman correlation coefficients with histologic grade; and (**b**) regression coefficients with survival outcome.

Table 2 contains the concordance indices from 3 imputation methods, as well as a random imputation baseline. Random imputation is performed by filling the missing values by random sampling the training data distribution. It shows that VAE and SVD are similar, and VAE and SVD achieve better concordance indices than KNN for both grade and survival outcome correlations. This suggests that VAE and SVD imputed data likely have better resemblances to true data in the context of a biomedical analysis for molecular biologists interested in specific genes in the presence of missing values. Fig. 4 illustrates a pairwise difference between the coefficients obtained from the ground truth and the coefficients obtained from the imputed data by KNN and VAE, respectively, and shows sharper peaks around 0 for VAE in all cases for histology and in most cases for survival. The pairwise differences are mostly distributed around 0, and a smaller variance around the 0 indicates that the pairwise differences are smaller overall. In each missing scenario VAE has a smaller variance than KNN across 10 trials (all *P* values < 0.005 in 2-sample *t*-tests).

**Table 2:** Correlation with clinical phenotypes

	KNN	VAE	SVD	Random
Spearman correlation coefficient with tumor histologic grade				
10% random missing	0.980 $\pm $ 0.001	0.982 $\pm $ 0.001	0.982 $\pm $ 0.001	0.950 $\pm $ 0.001
Highest GC content missing	0.949 $\pm $ $0.002$	0.958 $\pm $ 0.001	0.958 $\pm $ 0.001	0.816 $\pm $ 0.006
Entire genes missing	0.918 $\pm $ 0.005	0.932 $\pm $ 0.004	0.939 $\pm $ 0.005	0.500 $\pm $ $0.004$
Lowest value missing	0.977 $\pm $ $0.001$	0.983 $\pm $ $0.001$	0.986 $\pm $ 0.000	0.906 $\pm $ $0.007$
Cox regression coefficient with survival outcome				
10% random missing	0.969 $\pm $ 0.002	0.974 $\pm $ 0.001	0.972 $\pm $ 0.002	0.873 $\pm $ 0.050
Highest GC content missing	0.917 $\pm $ $0.006$	0.931 $\pm $ 0.004	0.933 $\pm $ 0.006	0.717 $\pm $ 0.016
Entire genes missing	0.851 $\pm $ 0.004	0.881 $\pm $ 0.005	0.906 $\pm $ 0.006	0.508 $\pm $ $0.010$
Lowest value missing	0.963 $\pm $ $0.002$	0.971 $\pm $ $0.002$	0.976 $\pm $ 0.002	0.842 $\pm $ $0.013$

### Imputation time for new samples

The computation time for SVD or KNN to impute a single sample scales linearly with the dimension of the entire data matrix; in comparison, a VAE model can be pre-trained and applied directly to any given new sample to impute missing values. Once a VAE model is trained, the time to impute a new sample is almost negligible. VAE thus has the benefit of reducing the computational cost, especially at evaluation time.

Benchmark experiments are done on a 20 core cluster with Intel Xeon 2.40 GHz CPUs, where the 3 methods are used to impute 100 samples in a gene expression matrix that consists of 6,600 samples and 17,176 genes. It takes an average of 2,800 seconds to train the VAE network. In terms of evaluation time, the KNN method takes 8,400 seconds on average, while SVD takes 36,900 seconds and VAE takes only 60 seconds, showing that VAE is several orders of magnitude faster at evaluation time.

### *β*-VAE and deterministic auto-encoder

**Figure 5:**
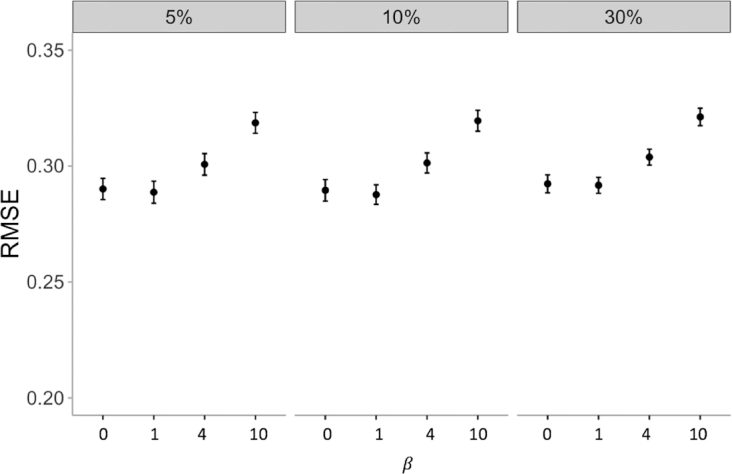
Imputation RMSE of $\beta $-VAE with 5%, 10%, and 30% random missing values of gene expression, with $\beta $ = 0, 1, 4, and 10, denoting the increasing strength of regularization.

We perform 3 random missing experiments with $\beta $-VAE and vary the hyperparameter $\beta $ between 0, 1, 4, and 10. Figure 5 shows that imputation results are similar for $\beta \ $= 0 and $\beta \ $= 1, while increasing $\beta $ to larger values worsens the prediction accuracies.

The fact that $\beta $ > 1 produces worse imputation errors leads us to the hypothesis that the total loss of VAE, shown on the right side of (3) and consisting of the reconstruction loss and regularization loss, may be considered a tradeoff between reconstruction quality and latent space coding efficiency. If a greater emphasis is put on latent space regularization, the reconstruction quality suffers. We conclude that stronger regularization does not help VAE's imputation performance.

Furthermore, when ${\rm{\beta \ }}$= 0, the imputation performance is similar to vanilla VAE (${\rm{\beta \ }} = {\rm{\ }}1)$. Therefore, for imputation, removing latent space regularization will not affect performance. From previous discussion in the $\beta $-VAE method section, the loss of $\beta $-VAE with $\beta \ $= 0 looks similar to that of a simple AE, but the key difference is that noise is injected to the latent space for $\beta $-VAE ($\beta \ $= 0). We find that with a simple AE, the imputation iterations cannot converge and the resulting RMSE is very large (not shown because of non-convergence). This suggests that the noise injection to the latent space enables the imputation ability of the VAE.

## Discussion

We have described a deep-learning imputation framework for transcriptome and methylome data using a VAE. We implement a shift-correction method to improve VAE imputation performance on a commonly encountered MNAR scenario. We demonstrate that the proposed framework is competitive with SVD, which is a time-inefficient method for real-world scenarios. We also show that VAE outperforms KNN in multiple scenarios, such as when using bulk transcriptome and methylome data. VAE thus can be an important tool to analyze the large amounts of publicly available data from thousands of studies, including RNA sequencing and microarray data that are publicly available in the Gene Expression omnibus [37].

We provide insights on the effect of latent space regularization on imputation performance. We show that increasing latent space regularization in the VAE implementation leads to larger errors, and thus should be avoided in the imputation tasks. In addition, the regularization of latent space can be removed without affecting VAE's performance in imputation.

We also found that noise addition to the latent space largely helps VAE's good imputation performance, compared to a regular deterministic AE. The method of noise injection during training is reminiscent of DAEs. However, the noise additions for VAE and DAE are different. First, the noise in VAE depends on the input, whereas the DAE noise is independent of the input. Second, although noise additions to intermediate layers have been proposed in stacked DAEs for the purpose of representation learning [29], in most data imputation applications noise has only been added to the input layer of DAE [27, 56]. In contrast, noise is added to the latent space layer in VAE. It is not in the scope of this paper to evaluate how different noise addition schemes impact imputation and to compare their performances. However, this may be worth exploring in future work.

Finally, in the context of imputing large data sets with high dimensional features, VAE has the potential benefit of reducing the computational cost at evaluation time, compared to SVD and KNN. This is because an AE model can be pre-trained and applied directly to new samples, while SVD and KNN require computing the entire matrix each time a new sample is given.

## Conclusion

In future work, it may be interesting to investigate VAE's application on single-cell RNA sequencing data, which has different missing scenarios than bulk RNA sequencing data. In addition, it may also be of interest to fully understand the effect of $\beta $ in $\beta $-VAE when $\beta $ is in the range from 0 to 1. Based on the hypothesis that there is a trade-off between reconstruction quality and desired latent space property regulated by $\beta $, it can be expected that removing the regularization term ($\beta \ $= 0) may even improve the vanilla VAE's ($\beta \ $= 1) imputation performance. It is worth noting that such phenomenon did not occur, which invites further study.

## Availability of source code and requirements

Project name: Genomic data imputation with variational auto-encoders

Project home page: https://github.com/gevaertlab/BetaVAEImputation.

Operating system(s): Platform independent

Programming language: Python

Other requirements: Python 3.6.6 or higher, Pytorch 0.4.1

License: BSD 3-Clause License

RRID:SCR_018730

BiotoolsID: betavaeimputation

## Availability of supporting data and materials

All data used in this manuscript are publicly available.

Gene expression data is version 2 of the adjusted pan-cancer gene expression data obtained from Synapse (synapse ID syn4976369) [57]. Clinical data of TCGA LGG/GBM can be found in Supplementary Table S1 in Ceccarelli et al. [55]. DNA methylation data is the WGBS data for BLUEPRINT methylomes (2016 release) obtained from rnbeads.org [58].

An archival copy of the code and supporting data is available via the *GigaScience* repository, GigaDB [59].

## Abbreviations

AE: auto-encoder; C: cytosine; CPU: central processing unit; DAE: denoising auto-encoder; G: guanine; GBM: glioblastoma; KNN: K-nearest neighbor; LGG: low-grade glioma; MCAR: missing completely at random; MNAR: missing not at random; RMSE: root mean squared error; SVD: singular value decomposition; TCGA: The Cancer Genome Atlas; VAE: variational auto-encoder.

## Competing interests

The authors declare that they have no competing interests.

## Funding

Research reported in this publication was supported by the Fund for Innovation in Cancer Informatics (www.the-ici-fund.org); the National Institute of Biomedical Imaging and Bioengineering of the National Institutes of Health (https://www.nibib.nih.gov/) grant numbers R01 EB020527 and R56 EB020527; and the National Cancer Institute (https://www.cancer.gov/) grant numbers U01 CA217851 and U01 CA199241, all to O.G. The content is solely the responsibility of the authors and does not necessarily represent the official views of the National Institutes of Health. The funders had no role in the study design, data collection and analysis, decision to publish, or preparation of the manuscript.

## Supplementary Material

giaa082_GIGA-D-20-00059_Original_Submission

giaa082_GIGA-D-20-00059_Revision_1

giaa082_GIGA-D-20-00059_Revision_2

giaa082_GIGA-D-20-00059_Revision_3

giaa082_Response_to_Reviewer_Comments_Original_Submission

giaa082_Response_to_Reviewer_Comments_Revision_1

giaa082_Response_to_Reviewer_Comments_Revision_2

giaa082_Reviewer_1_Report_Original_Submission Gregory Way -- 4/9/2020 Reviewed

giaa082_Reviewer_1_Report_Revision_1 Gregory Way -- 5/31/2020 Reviewed

giaa082_Reviewer_2_Report_Original_Submission Natalie Davidson -- 4/21/2020 Reviewed

giaa082_Reviewer_2_Report_Revision_1 Natalie Davidson -- 5/27/2020 Reviewed

giaa082_Reviewer_2_Report_Revision_2 Natalie Davidson -- 6/16/2020 Reviewed

giaa082_Supplemental_Figure_and_Table

## Supplementary Data

**Figure S1**. Model performances for 6 architectures: 3 hidden layers with latent sizes of 50, 200, and 400, and 5 hidden layers with latent sizes of 50, 200, and 400. Experiments are conducted on a simulated 10% random missing case.

**Table S1**. RMSEs from each method for each individual gene from 1 experimental trial in the second MNAR scenario for RNA sequencing data.
