# Supplementary material for: Genomic data imputation with variational auto-encoders
Source: Gigascience. 2020 Aug 6;9(8):giaa082. doi: 10.1093/gigascience/giaa082 (PMC7407276; doi:10.1093/gigascience/giaa082)

GigaScience
Genomic data imputation with variational autoencoders
--Manuscript Draft--

**Manuscript Number:**	GIGA-D-20-00059R1
**Full Title:**	Genomic data imputation with variational autoencoders
**Article Type:**	Technical Note
**Funding Information:**	National Institute of Biomedical Imaging and Bioengineering (R01 EB020527) — Mr. Olivier Gevaert<br>National Institute of Biomedical Imaging and Bioengineering (R56 EB020527) — Mr. Olivier Gevaert<br>National Cancer Institute (US) (U01 CA217851) — Mr. Olivier Gevaert<br>National Cancer Institute (U01 CA199241) — Mr. Olivier Gevaert
**Abstract:**	As missing values are frequently present in genomic data, practical methods to handle missing data are necessary for downstream analyses that require complete datasets. State-of-the-art imputation techniques including Singular Value Decomposition (SVD) and K-Nearest Neighbors (KNN) based methods can be computationally expensive for large datasets and it is difficult to modify these algorithms to handle certain missing-not-at-random cases. In this work, we use a deep learning framework based on the variational autoencoder (VAE) for genomic missing value imputation and demonstrate its effectiveness in transcriptome and methylome data analysis. We show that in the vast majority of our testing scenarios, VAE achieves similar or better performances than the most widely used imputation standards, while having computational advantage at evaluation time. When dealing with missing-not-at-random, e.g. low values are missing, we develop simple yet effective methodologies to leverage the prior knowledge about missing data. Furthermore, we investigate the effect of varying latent space regularization strength in VAE on the imputation performances, and in this context show why VAE has a better imputation capacity compared to a regular deterministic autoencoder (AE).
**Corresponding Author:**	Olivier Gevaert<br><br>UNITED STATES
**Corresponding Author Secondary Information:**	
**Corresponding Author's Institution:**	
**Corresponding Author's Secondary Institution:**	
**First Author:**	Yeping Lina Qiu
**First Author Secondary Information:**	
**Order of Authors:**	Yeping Lina Qiu<br>Hong Zheng<br>Olivier Gevaert
**Order of Authors Secondary Information:**	
**Response to Reviewers:**	Response to reviewers<br><br>We very much appreciate the reviewers' great efforts to scrutinize our study and raise important questions and suggestions. Their constructive comments are important and have helped us greatly to improve the quality of this manuscript. We have revised the manuscript following both reviewers' suggestions. In the following, we reply to the reviewers' comments.

Powered by Editorial Manager® and ProduXion Manager® from Aries Systems Corporation

Reviewer #1

Major Concerns:

1. What was the rationale for including a Beta-VAE in this study? The authors nicely explain "If a greater emphasis is put on latent space regularization, the reconstruction quality suffers". Why would a Beta-VAE be suitable for an imputation task then? Additional rationale about including the Beta-VAE model would be helpful.

We agree it is important to explain more clearly about the rationale for including Beta-VAE in our study. We made some modifications in the "Materials and Methods -> β-VAE" section, and reorganized the "Results -> β-VAE and deterministic autoencoder" section, so that it would hopefully be more clear why we thought it necessary to include the β-VAE analysis.

β-VAE ($\beta > 1$) has been shown to perform better than VAE in certain image generation tasks and has attracted increasing research interest. However, no prior work has investigated the effect of β on imputation. Since VAE can be considered as a special case of β-VAE, we extend our study to β-VAE with a varying β to further understand the effect of regularization on VAE imputation, and to investigate potential possibility to increase its performance.

β-VAE did not turn out producing better imputation results. However, the study gave us insights in the aspects below (which are also explained in the results section).

1) The effect of regularization in VAE is not clearly known in advance. The result that $\beta > 1$ produces worse imputation errors leads us to the hypothesis that the total loss of VAE may be considered as a tradeoff between reconstruction quality and latent space coding efficiency. If a greater emphasis is put on latent space regularization, the reconstruction quality suffers. We therefore conclude that stronger regularization does not help VAE's imputation performance.

2) Furthermore, when $\beta=0$, the imputation performance is similar to vanilla VAE ($\beta=1$). Therefore, for imputation, removing latent space regularization will not affect performance. From the discussion in the β-VAE method section, the loss of β-VAE with $\beta=0$ looks similar to that of a simple AE, but the key difference is that noise is injected to the latent space for of β-VAE ($\beta=0$). We find that with a simple AE, the imputation iterations cannot converge and the resulting RMSE is very large (not shown because non-convergence). This suggests that the noise injection to the latent space largely helps the imputation ability of the VAE.

Studying β-VAE helps us disentangle the different components in the loss function of VAE, and subsequently gain more insights on VAE's ability to impute values.

2. The simulations are well thought out and mostly described thoroughly (see minor concerns below). However, the simulations are missing important baselines and scenarios closer to real-world applications. For example, the VAE-shift-correction performance comparison is only shown in panel d in Figure 1. Is it true that in a real-world case, a user would default to using the VAE-shift-correction model? If so, the authors should add the VAE-shift-correction performance to the other simulation tasks. Also, the "colmeans" performance shown in the DNA methylation graph is informative. The authors should add a similar baseline in the gene expression evaluation.

Per the reviewer's suggestion, we added a brief method description for Colmeans in the "Evaluation methods" section, and added "Colmeans" results in all simulation scenarios for RNA sequencing data (Figure 1).

We made modifications in the "Variational autoencoder imputation with shift correction" and "Missing data simulations" sections to clarify the different missing simulations.

Powered by Editorial Manager® and ProduXion Manager® from Aries Systems Corporation

The VAE-shift-correction only applies to the particular cases with prior knowledge. Without prior knowledge, VAE should be used as default. Panels A-D in Figure 1 each simulates a different missing case motivated by real world scenarios under different conditions. Panels A-C all belong to the VAE application, while Panel D belongs to the VAE-shit-correction application.

When we have some prior knowledge about the sequencing technology and parameters, we may be able to make some assumptions about the missing scenarios. When certain experimental conditions (e.g., low RNA sequencing depth) allow us to make assumptions that the majority of missing values are low expression values, and that the missing values are shifted from the seen value distribution, we can then choose to use VAE-shift-correction. A shift-correction model is robust in the sense that once a model is trained to accommodate shifting, its performance is not dramatically worsened if the actual testing data has larger or smaller shifting than the data it is trained on, and therefore we do not need an exact prior knowledge how much shifting has occurred in the data in order to train a model. If we do not know anything about the data types or experimental conditions that possibly cause shifts, it is advised to use the VAE without shift-correction.

3. The correlation analyses require more detail in order to adequately interpret. The authors report concordance between real and imputed values in two tasks: 1) Pearson correlation to tumor grade and 2) Cox regression coefficient with survival outcome. What are the ground truth estimates? Only the differences (fig 4) and concordance (table 1) to ground truth are shown. I imagine that ground truth correlations could be quite low. A low ground truth correlation would make it easy for random imputations to have high concordance and low difference. In addition to reporting the ground truth correlations, the authors should also add a random imputation baseline. Also, the purpose of figure 4 is to highlight the "sharper peaks around zero". This is not immediately clear for all comparisons. Is there a statistical test to confirm this observation? Lastly, in Table 1, since there are 10 different iterations, shouldn't the values have ranges?

We addressed all the issues accordingly in “Correlation with clinical phenotypes”:

Ground truth correlations are described. They are not overwhelmingly low and the concordance indices comparisons are considered meaningful.

Random imputations results are added as a baseline in Table 2 (originally Table 1).

To confirm there are sharper peaks around zero for VAE than for KNN, in Figure 4, we compare the variances of the distributions across ten trials. A smaller variance indicates sharper peak. Student's t-test shows smaller variances for VAE than KNN in all cases with $p<0.005$.

In Table 2, we added 95% CI range for the values.

4. Model training details are absent from the methods. What is the VAE architecture (how many layers? How many latent dimensions?) What are the hyperparameters (learning rate, batch size, epochs, etc.)? Was cross validation performed? How did the authors select the size of the latent dimensions? Was there any attempt to improve model performance? These details are absolutely critical. It would also be informative to view imputation performance across rounds of decoding iterations.

We added the model training details in the “Model parameters and hyper-parameters tuning” section. We described how hyper-parameters (optimizer, learning rate, batch size, epochs, imputing iterations), and model parameters (layer, dimension) are chosen on the validation data. We also described how validation data is created in the “Missing data simulations”.

5. More description of the clinical data is required. Where was this data retrieved from? Were there any additional data processing required?

We added the source of clinical data in “Availability of Data and Materials”, and also included more details about the format and processing of clinical data in “Evaluation methods”.

Powered by Editorial Manager® and ProduXion Manager® from Aries Systems Corporation

Minor Concerns:

6. In general, there may be too much detail describing the VAE and Beta-VAE. It is actually somewhat distracting. Would a citation and brief description suffice? The innovation in the manuscript is the imputation application, not the models.

We hope to provide not only a useful application, but also some insights on why the model works well, which may possibly facilitate future effort to improve the model further. Since our hypothesis and conclusions are mostly based on the fundamentals of VAE/beta-VAE, we hope the readers do not have to search extensively for their details elsewhere in order to understand them. The details of VAE and Beta-VAE may be helpful for readers to appreciate our conclusions and results better, and we therefore respectfully think that they should be included.

7. The term "covariate shift" is not specific. Please use a different term or define more specifically.

We replaced the term with a more specific description: “a missing-not-at-random scenario where the missing data distribution is not the same as the seen data”.

8. What is deterministic about an autoencoder? This part confused me. What implementation is being used? Autoencoders are typically initialized randomly and then trained using gradient descent.

Autoencoders are indeed initialized randomly and trained with gradient descent. By “deterministic”, we meant that there is no probabilistic modeling of the latent space, which is fundamentally different from a variational autoencoder. To clarity this point, we added a more detailed description in the “Variational autoencoder” section: “While in a regular autoencoder the latent space is encoded and then decoded deterministically, i.e., there is no probabilistic modelling of the latent space, a variational autoencoder (VAE) learns a probability distribution in the latent space.”

9. I think the simulation tasks are sufficient, but the authors decided to remove genes with missing values in a preprocessing step. How many genes were removed? What is the state of overall missingness? Is there a way to assess the added benefit of applying the VAE imputation in a real-world scenario with real missing genes? This would make for a compelling argument that imputation should occur more regularly. Since the authors are using GBM and LGG exclusively, is it possible to design some sort of a subtype clustering experiment comparing existing subtype labels and measuring an adjusted Rand index with and without imputation? (comparing of course to randomized imputation) Perhaps this is beyond the scope, but would be quite compelling.

We added description of the missing status of the original gene expression data in the section “Datasets”. The raw RNA sequencing data has a feature dimension of 20531 genes but contains NA values in 15% of the genes. Within the 15% of the genes who have missing values, on average 8.5% of the values are missing. The NA values are introduced in the pre-processing pipeline produced by Synapse.

We agree that it would be very interesting to assess imputation on the raw data without ground truth and investigate the impact of imputation on real-world biological scenarios other than histological grades and survival outcome, however, we also think that it belongs to a wider scope that is not the focus of this paper.

10. In the random 5% of genes simulation, is this 5% of all 17,176 genes without sampling restriction? It is surprising to me that the RMSE in figure 1C is so low. Perhaps because what's being plotted is actually mean RMSE? If individual gene RMSE is plotted, what does the distribution look like? This result probably has better real-world implications since a portion of the target audience is interested in imputing

Powered by Editorial Manager® and ProduXion Manager® from Aries Systems Corporation

individual genes and might care more about the range of imputation than the average imputation.

Yes, there is no sampling restriction on 5% of the genes.

The plot is mean RMSE. Since there are 858 individual genes for each trial, we thought it is difficult to plot individual distributions and to compare across methods using individual distributions. Therefore, we consider the mean RMSE plot as a more practical overall graphic representation, which also allows us to compare across methods more easily. However, individual distributions for certain genes can be available upon request for target audience.

11. In the DNA methylation missing data simulations methods, the authors state: "We set the coverage threshold to six in our experiments". What units are being considered? 6 CpG sites per gene? What constitutes the gene region? Only CpG on gene bodies?

We clarified the definition of coverage threshold in the “Missing data simulations” section.

The coverage refers to the number of reads that can be mapped to a specific CpG site. Since we are using bisulfite sequencing data for DNA methylation, in the analysis, for each CpG site, we count how many reads supporting methylated status, and how many reads supporting unmethylated status. Some CpG sites may have very few reads mapped to them, which undermines the confidence in the measurement of methylation level. Thus, we choose an arbitrary threshold of six reads for the methylation status of a CpG site to be confidently determined. Methylation levels of CpGs with less than six reads mapped to them are treated as missing values in the analysis.

12. The simulation scenario is great! It is particularly nice to see 10 random trials being used for each comparison. A table describing simulation experiments could be very helpful.

A table is a great way to elucidate simulation experiments. We added Table 1 in the “Missing data simulations”.

13. In the imputation procedure during VAE training, the authors state: "Initially, the missing values are replaced with random values.". I don't think this is true. There are bounds placed on the random sampling, correct? What are these bounds? In this same paragraph, what is the iteration threshold?

Agreed. We clarified the initialization procedure and specified that the missing values are replaced with random values sampled from a standard Gaussian distribution.

The iteration threshold is also further clarified in the “Model parameters and hyper-parameters tuning” section. The number of iterations to perform the iterative imputation is also determined empirically. The imputed values are found to converge very quickly, and results remain mostly stable after 2 or 3 iterations. We use 3 as the iteration threshold.

14. The authors state: "the testing data is scaled by the training data mean and variance before the imputation iterations, and inverse scaled after imputation". This is not totally clear to me. For the testing data to be a true test set, it should not be influenced by the training set.

The mean and variance of training data can be considered as scaling parameters that are learnt from the training data. They can be used to scale any testing data for imputation. In this way, we are not tempering the testing data with any specific distribution of the testing data itself. This is a preprocessing step with a knowledge built in the model itself. We respectfully maintain that this is a fair operation.

Powered by Editorial Manager® and ProduXion Manager® from Aries Systems Corporation

15. The authors state: "Since the nature of the shift is relatively simple and known in advance, we leverage this knowledge to correct the shifting". The authors should elaborate on this point. In a real world, missing not at random case, how is the nature of the shift known in advance?

This point has been addressed in our response to the number two comment in “Major concerns”. Some conditions may lead to possible certain missing-not-at-random cases, for example, the sequencing depth in the RNA sequencing procedure may give us a measure of the degree of low expression level missing. We explained more about this in “Variational autoencoder imputation with shift correction”.

16. In the results section, the authors state "In all tested random missing scenarios VAE achieves better RMSE than KNN, and reaches similar or better performances [sic] than SVD. This is true in all cases except for 30% correct (Figure 1a)?

That is correct. In 30% random missing case and high GC content missing case, VAE and SVD are similar in performance ($p > 0.02$), and so we modified the statement to be clearer: “VAE achieves better RMSEs than KNN in all tested missing scenarios, and reaches similar or better performances than SVD in most scenarios”.

17. The deeper investigation into the shift correction approach is innovative and interesting! It is nice to see that the correction parameter is not very sensitive, and would indeed provide benefit in real world scenarios. The authors should add the shift correction VAE to panels A-C in figure 1 to further demonstrate its robustness.

This point has been addressed in our response to the reviewer’s second comments. The shift correction model is not intended for A-C scenarios.

18. There are a few instances of misspelled words and incorrect grammar throughout the manuscript. For example, the sentence "Random half of the genes whose GC content are in the top 10% miss their values in the testing data" is grammatically incorrect. Also, watch for spelling in "for missing-comeletely-at-random and block missing cases…". The authors should carefully reread and correct these errors.

We have proofread the manuscript carefully and corrected such errors.

19. Please provide which version of the EB++AdjustPANCAN data on synapse was used.

Version 2 is used, and this information is added in “Availability of Data and Materials”.

20. Please provide exactly which data in the rnbeads.org site was used.

DNA methylation data is the WGBS data for BLUEPRINT methylomes (2016 release). This is added in “Availability of Data and Materials”.

21. It is great that the authors have provided their source code (and an open source license!) in a github repository. If possible, additional information on how the analysis can be reproduced (including how the scripts should be executed) with would be helpful.

We added a README file in the repository that explains how the analysis can be carried out.

Reviewer #2:

Powered by Editorial Manager® and ProduXion Manager® from Aries Systems Corporation

Data Questions:

1. What are the NA values in TCGA data? Were the NAs genes that had a count of zero? Did the authors do any additional filtering, I'm a bit surprised the remaining number is 17K.

The raw RNA sequencing data has a feature dimension of 20531 genes but contains NA values in 15% of the genes. Within the 15% of the genes who have missing values, on average 8.5% of the values are missing. The NA values are introduced in the pre-processing pipeline produced by Synapse. We did not do any additional filtering. We added this information in the “Datasets” section. In our study, in order to have a ground truth to evaluate imputation accuracies, we removed the NA values and carried out analysis with complete data. The missing values in our study were artificially introduced.

2. The authors mention disease type is how the RNA-Seq data was separated, what granularity of disease type? Is this high-level Cancer Types or do the authors separate by any sub-types?

The RNA-Seq data is glioma samples consisting of LGG and GBM. It is stratified by glioma subtypes (i.e., LGG versus GBM). We clarified this point in the “Missing data simulations” section.

Clinical correlation Questions:

3. The authors run a correlation analysis between clinical phenotypes and the imputed values. Additional details would help clarify how to interpret the results. For example, was this analysis done within cancer types or across cancer types? How were different histological grades transformed into values? Also, you mention that the correlation was initially done using spearman, however later you mention Pearson in the figure legends. Which package did you use to run this? If I want to redo your analysis, I need more details. I wasn't able to find further results in your GitHub, either.
Also, it is a little unclear to me what your motivation is to look at the concordance index between the correlation coefficients obtained from the imputed data. Why not look at which of the imputation methods provides values that are most predictive of the clinical phenotypes? Is it also possible to get error bars on the values in table 1?

The reviewer’s questions help us make things clearer in this section. We addressed all the issues accordingly in “Correlation with clinical phenotypes”:

The analysis was done with the TCGA glioma cohort containing both LGG and GBM samples.
The tumor grade and survival information for each brain tumor patients are publicly available (added data source in the availability section). The histologic grade variable in the TCGA brain tumor data contains three levels: Grade II, III and IV, indicating increasing level of tumor malignancy. We directly use the grade value as an ordinal variable of three levels, and calculate the Spearman correlation coefficient between each gene and the grade variable. “Pearson” is a typo in the figure legends and we corrected the error. We used “spearmanr” package in python to do this analysis. The script to carry out this analysis is added in the GitHub repository.
The 95% CI range for the values are added in Table 2 (originally Table 1).
In our analysis, we would like to limit our evaluation to measuring how much the imputed data resembles the ground truth. Concordance indices are an alternative way to evaluate which methods produce the imputed data that may resemble the ground truth better clinically. Building good predictive models with the imputed data (either univariate or multivariate) is usually a next step in biomedical data analysis. However, that is beyond the scope of this manuscript.

Plotting Questions:

4. To be more convincing that the author's method performs better, all boxplots where

Powered by Editorial Manager® and ProduXion Manager® from Aries Systems Corporation

you compare against other methods require significance scores. (I think you are using ggplot, so it would be easy to add these using the ggsignif package).

We appreciate the reviewer's suggestion of the "ggsignif" package. We added significance scores (comparing VAE and other methods) in all the plots.

Training / Model Questions:

5. In regards to the shirt parameter, it is not clear to me how lambda was selected, the authors state "hyperparameter is selected on a validation data which simulates the lowest 10% missing case". What is the validation data in this case and how exactly is lambda learned? Is the validation data a completely held-out set that isn't used later? Did you do this shifted VAE for methylation data?

We clarified the definition of validation data in "Missing data simulations", and explained lambda selection in more details in "Variational autoencoder imputation with shift correction".

Each dataset is split into 80%-20% for training and hold-out testing in the imputation framework. The training dataset is further split into 80%-20%, where 20% is the validation dataset for hyper-parameter tuning. After hyper-parameters are selected, the entire training set is used for training.

To test the lowest 10% missing case, we simulate a 10% lowest value missing scenario on the validation dataset, and select the shift correction parameter value that produces the smallest validation error.

We did not use shift-correction VAE for methylation data. Each of the MNAR simulation is motivated by a different real-world condition specific to either gene expression data or methylation data. For RNA sequencing data, when the RNA sequencing depth is relatively low, genes that have low expression levels may not be detected because the few reads generated from this gene may not be captured. Therefore, we consider a possible scenario where lowly expressed genes are prone to be missing. For DNA methylation data, such scenario is not applicable. Instead, we simulate a different MNAR scenario where we mask CpG sites that have fewer coverage than a certain threshold. The coverage refers to the number of reads that can be mapped to a specific CpG site. Since we are using bisulfite sequencing data for DNA methylation, in the analysis, for each CpG site, we count how many reads supporting methylated status, and how many reads supporting unmethylated status. Some CpG sites may have very few reads mapped to them, which undermines the confidence in the measurement of methylation level. In such cases, the missing values themselves are not the lowest values as in the RNA sequencing data's case. Therefore, we did not apply the shift-correction to methylation data. We clarified the shift-correction model's use case in the "Variational autoencoder imputation with shift correction" and "Missing data simulations" sections.

6. What is the size of the encoder? How many hidden layers? What is the activation function?

We added the model training details in the "Model parameters and hyper-parameters tuning" section. We use a AE with five hidden layers and bottle neck size of 200. The activation function is ReLU. Other details including learning rate, batch size etc. are also included in this section.

General comments:

7. Reading this paper in the context of current genomics research, it may be useful to compare against a model in the wide array of single-cell data imputation models. This is an application where I can see the author's method being applied.

We agree that single-cell data imputation is an important application in genomics research. Single-cell data, however, has different missing case from bulk data, and

Powered by Editorial Manager® and ProduXion Manager® from Aries Systems Corporation

	applying the model on single-cell data will be a change of focus for this study.<br><br>A lot of historical bulk data are available for analysis through databases like the Gene Expression Omnibus (GEO) and the Short Read Archive (SRA). We also think that in the future, bulk data will still be important through the deconvolution of bulk gene expression. We therefore would like to have this manuscript focus on bulk data applications. However, we think that single-cell RNA sequencing data applications can be an interesting work in the future, and we included it when discussing future work in the conclusion section.<br><br>8. Also, I feel the statement "We show that noise addition to the latent space is the essential mechanism that enables VAE's good imputation performance, compared to a regular deterministic AE", is a bit strong. I think the authors have an experiment that may suggest this, but they did not show it was an essential mechanism.<br><br>We agree with the reviewer's comment, and modified the statement. The revised sentence is "We also found that noise addition to the latent space largely helps VAE's good imputation performance, compared to a regular deterministic AE."<br><br>9. There are also some spelling mistakes, "form" instead of from in "Variational autoencoder imputation with shift correction", and "missing-no-at-random" in "RMSE of imputation on RNA sequencing data"<br><br>We have proofread carefully and corrected such errors.
**Additional Information:**	
**Question**	**Response**
Are you submitting this manuscript to a special series or article collection?	No
**Experimental design and statistics**<br><br>Full details of the experimental design and statistical methods used should be given in the Methods section, as detailed in our Minimum Standards Reporting Checklist. Information essential to interpreting the data presented should be made available in the figure legends.<br><br>Have you included all the information requested in your manuscript?	Yes
**Resources**<br><br>A description of all resources used, including antibodies, cell lines, animals and software tools, with enough information to allow them to be uniquely identified, should be included in the Methods section. Authors are strongly encouraged to cite Research Resource Identifiers (RRIDs) for antibodies, model	Yes

organisms and tools, where possible.<br><br>Have you included the information requested as detailed in our Minimum Standards Reporting Checklist?	
**Availability of data and materials**<br><br>All datasets and code on which the conclusions of the paper rely must be either included in your submission or deposited in publicly available repositories (where available and ethically appropriate), referencing such data using a unique identifier in the references and in the “Availability of Data and Materials” section of your manuscript.<br><br>Have you have met the above requirement as detailed in our Minimum Standards Reporting Checklist?	Yes

Powered by Editorial Manager® and ProduXion Manager® from Aries Systems Corporation

Manuscript

Click here to access/download;Manuscript;manuscript revision.docx

Genomic data imputation with variational autoencoders

Yeping Lina Qiu[1,2], Hong Zheng[1], Olivier Gevaert[1,3,*]

[1] Stanford Center for Biomedical Informatics Research (BMIR), Department of Medicine, Stanford University, Stanford, CA USA

[2] Department of Electrical Engineering, Stanford University, Stanford, CA, USA

[3] Department of Biomedical Data Science, Stanford University, Stanford, CA, USA

* To whom correspondence should be addressed: ogevaert@stanford.edu

Abstract

As missing values are frequently present in genomic data, practical methods to handle missing data are necessary for downstream analyses that require complete datasets. State-of-the-art imputation techniques including Singular Value Decomposition (SVD) and K-Nearest Neighbors (KNN) based methods can be computationally expensive for large datasets and it is difficult to modify these algorithms to handle certain missing-not-at-random cases. In this work, we use a deep learning framework based on the variational autoencoder (VAE) for genomic missing value imputation and demonstrate its effectiveness in transcriptome and methylome data analysis. We show that in the vast majority of our testing scenarios, VAE achieves similar or better performances than the most widely used imputation standards, while having computational advantage at evaluation time. When dealing with missing-not-at-random, e.g. low values are missing, we develop simple yet effective methodologies to leverage the prior knowledge about missing data. Furthermore, we investigate the effect of varying latent space regularization strength in VAE on the imputation performances, and in this context show why VAE has a better imputation capacity compared to a regular deterministic autoencoder (AE).

Introduction

The massive and diverse datasets in genomics have provided researches a rich resource to study the molecular basis of diseases. The profiling of gene expression and DNA methylation have enabled the identification of cancer driver genes or biomarkers (Byron, et al., 2016; Gevaert, et al., 2015; Kulis and Esteller, 2010; Litovkin, et al., 2015; Tomczak, et al., 2015; Zheng, et al., 2019). Many such studies on cancer genomics require complete datasets (Champion, et al., 2018). However, missing values are frequently present in these data due to various reasons including low resolution, missing probes, and artifacts (Baghfalaki, et al., 2016; Libbrecht and Noble, 2015). Therefore, practical methods to handle missing data in genomic datasets are needed for effective downstream analyses.

One way to complete the data matrices is to ignore missing values by removing` the entire feature if any of the samples has a missing value in that feature, but this is usually not a good strategy as the feature may contain useful information for other samples. The most preferable way to handle missing data is to impute their values in the pre-processing step. Many approaches have been proposed for this purpose (Moorthy, et al., 2019), including replacement using average values, estimation using weighted K-nearest neighbor (KNN) method (Faisal and Tutz, 2017; Troyanskaya, et al., 2001), and estimation using singular value decomposition (SVD) based methods (Troyanskaya, et al., 2001). KNN and SVD are two techniques that have been commonly used as benchmarks against new developments (Smaragdis, et al., 2011; Yu, et al., 2010). KNN imputes missing value of a feature in a given sample with the weighted average of the feature values in a number of similar samples, as calculated by some distance measure. SVD attempts to estimate data structure from the entire input including the samples with missing

values, and fill in the missing values iteratively according to the global structure. For this reason, SVD is inefficient on large matrices in practice since new decompositions have to be estimated for each missing sample, which is a very time-consuming process. However, SVD serves as an important benchmarking method to determine how well other, faster methods perform compared to SVD.

In recent years, a branch of machine learning which emerged based on big data and deep artificial neural network architectures, usually referred to as deep learning, has advanced rapidly and shown great potential for applications in bioinformatics (Min, et al., 2017). Deep learning has been applied in areas including genomics studies (Arisdakessian, et al., 2019; Chen, et al., 2016; Leung, et al., 2014), biomedical imaging (Chen, et al., 2016), and biomedical signal processing (Wulsin, et al., 2011). Autoencoders (AE) are a deep learning based model which form the basis of various frameworks for missing value imputation, and they have shown promising results for genomic data, imaging data and industrial data applications (Beaulieu-Jones and Moore, 2017; Eraslan, et al., 2019; Jaques, et al., 2018; Mattei and Frellsen, 2019; McCoy, et al., 2018; Vincent, et al., 2008). However, a simple AE without regularization is rarely ranked among the competitors for data imputation (Costa, et al., 2018; Garciarena and Santana, 2017). When a simple AE only focuses on creating output close to the input without any constraints, the model may overfit on the training data instead of learning the latent structure, such as dependencies and regularities characteristic of the data distribution (Vincent, et al., 2008), which makes it unlikely to impute well given new samples. Denoising autoencoder (DAE) is a type of autoencoder that specifically uses noise corruption to the input to create robust latent features (Vincent, et al., 2008). DAE has been extensively used in the application of data imputation (Beaulieu-Jones and Moore, 2017; Costa, et al., 2018). The corrupting noise

introduced in the DAE can be in many different forms, such as masking noise, Gaussian noise, and salt-and-pepper noise (Vincent, et al., 2010).

Variational autoencoders (VAE) are a probabilistic autoencoder that has wide applications in image and text generation (Hu, et al., 2017; Kingma and Welling, 2013; Yeh, et al., 2017). VAE learns the distributions of latent space variables that make the model generate output similar to the input. VAE has primarily been used as a powerful generative tool, having the ability to produce realistic fake contents in images, sound signal or texts, that highly resemble the real life contents that they learn from. The generative power is made possible by regularizing the latent space (Kingma and Welling, 2013). Constraining the latent space distributions to be close to a standard Gaussian helps to achieve a smooth latent space where two close points in the latent space should lead to similar reconstructions, and any point sampled from the latent space should give a meaningful reconstruction (Ghosh, et al., 2019). VAE has been applied in genomic contexts such as latent space learning of gene expression data (Way and Greene, 2017). In addition, recent works have applied VAE on single cell RNA sequencing data for clustering, batch correction and differential expression analysis (Grønbech, et al., 2018; Lopez, et al., 2018). However, VAE has not been extensively studied for genomic data imputation for bulk RNA expression and DNA methylation data, while large amounts of retrospective genomic and epigenomic data are available through databases like the Gene Expression Omnibus (GEO) (Barrett, et al., 2012) and the Short Read Archive (SRA)(Wheeler, et al., 2006).

Here, we examine the VAE mechanism and its application to genomic missing value imputation with bulk transcriptome and methylome data. We show that for both missing-completely-at-random and missing-not-at-random cases in transcriptome data and methylome

data, VAE achieves similar or better performances than the de facto standards, and thus is a strong alternative to traditional methods for data imputation (Aghdam, et al., 2017). We demonstrate that in a missing-not-at-random scenario where the missing data distribution is not the same as the seen data, a shift correction method can be implemented to improve VAE's extrapolation performance. Furthermore, we investigate the effect of latent space regularization on imputation with a generalization of the variational autoencoder - β-VAE (Higgins, et al., 2017). In the context of β-VAE results, we provide insights on why VAE can achieve good imputation performance compared to a regular deterministic AE.

Materials and Methods

Datasets

We use two datasets to perform data imputation: pan-cancer RNA sequencing data from The Cancer Genome Atlas (TCGA) datasets (Malta, et al., 2018; Tomczak, et al., 2015), and DNA methylation data (Campbell, et al., 2018; Gevaert, et al., 2015; Stunnenberg, et al., 2016). Both datasets contain only numeric values. The RNA sequencing data is expressed in RPKM (Reads Per Kilobase of transcript, per Million mapped reads), which is a normalized unit of transcript expression. The DNA methylation data contains the numeric values of the methylation level at each CpG site. The RNA sequencing data has a feature dimension of 20531 genes. There are 15% of the genes containing more or less NA values, while the rest of the 85% of the genes are complete. Within the 15% of the genes who have missing values, on average 8.5% of the values are missing. The NA values are introduced in the pre-processing pipeline produced by Synapse. In order to have a ground truth to evaluate the missing value imputation frameworks, we remove the 15% genes with NA values in our pre-processing, which results in a feature

dimension of 17176 genes. We then normalize the data by log transformation and z-score transformation. We use 667 glioma patient samples, including glioblastoma (GBM) and low-grade-glioma (LGG), to train and test the missing value imputation framework. In pre-processing the DNA methylation data, we remove the NA values, and normalize the data by negative log transformation and z-score transformation. We use the smallest chromosome subset (Chromosome 22) so that the resulting data dimension is not prohibitive for benchmarking different computation methods. The resulting data has 21220 CpG sites and 206 samples.

Missing data simulations

Each dataset is split into 80%-20% for training and hold-out testing. The training dataset is further split 80%-20%, where 20% is the validation dataset for hyper-parameter tuning. After hyper-parameters are selected, the entire training set is used for training. The sample split for the RNA sequencing dataset is stratified by the glioma subtypes (LGG versus GBM), and the split is random for the DNA methylation data since the samples are homogenous. The training data is a complete dataset without missing values. Missing values are introduced to the testing data in two forms: missing-completely-at-random (MCAR) and missing-not-at-random (MNAR) (Little and Rubin, 2019) (Table 1).

In the MCAR cases, we randomly mask a number of elements in each row by replacing the original values with NAs. To test a range of missing severity, we make the number of masked elements amount to 5%, 10%, and 30% of the total number of elements respectively.

Each of the MNAR simulation is motivated by a different real world condition specific to either gene expression data or methylation data. For the gene expression data, we simulate three MNAR scenarios, each of which has 5% of the total data values missing. In the first scenario, the

masked values are concentrated at certain genes. Such genes are selected based on their GC content, which is the percentage of nitrogenous bases on a RNA fragment that are either guanine (G) or cytosine (C). Too high or too low GC content influences RNA sequencing coverage, and potentially results in missing values from these genes (Chen, et al., 2013). We select genes with GC-content at the highest 10% and randomly mask half of these values. In the second simulation case, certain genes are masked entirely. We randomly select 5% of the genes and mask all values from these genes in the testing data, and as a result, the corrupted data miss all values for specific genes. The third scenario is based on gene expression level. When the RNA sequencing depth is relatively low, it is relatively easy to miss genes that have low expression levels, because the reads, generated from those genes, are too few to be captured during sequencing (Conesa, et al., 2016). Therefore, we consider a possible scenario where lowly expressed genes are prone to be missing. In the testing data we first choose gene expression values at the lowest 10% quantile, and then randomly mask half of these values.

For the DNA methylation data, we simulate two MNAR scenarios. The first scenario is complete missing of certain CpG sites, which is similar to the second MNAR case in gene expression data, where we select 5% of the features and mask them entirely in the testing data. In the second case, we mask CpG sites that have fewer coverage than a certain threshold. Some CpG sites may have very few reads mapped to them, which undermines the confidence in the measurement of methylation level. Thus, we choose an arbitrary coverage threshold of six reads for the methylation status of a CpG site to be confidently determined. Methylation levels of CpGs with less than six reads mapped to them are treated as missing values in the analysis here.

For each simulation scenario described above, we create ten random trials to measure the average imputation performance. The uncorrupted testing data is used to compute the imputation RMSE.

Table 1. Simulation experiments on RNA sequencing data and DNA methylation data

**Data**	**Missing type**	**Missing scenario**
RNA sequencing data	MCAR	5% completely random missing
		10% completely random missing
		30% completely random missing
	MNAR	50% random missing in genes with the highest 10% GC content
		5% genes are entirely missing
		50% random missing in genes with the lowest 10% expression level
DNA methylation data	MCAR	5% completely random missing
		10% completely random missing
		30% completely random missing
	MNAR	5% CpG sites are entirely missing
		50% random missing in CpG sites with coverage lower than 6 reads

Variational autoencoder

An autoencoder is an unsupervised deep neural network that is trained to reconstruct an input X by learning a function $h_{w,b}(X) \approx X$. This is done by minimizing the loss function

between the input X and the network's output X': $L(X, X')$. The most common loss function is the root mean squared error:

$$L(X, X') = \sqrt{||X - X'||^2} \qquad (1)$$

An autoencoder consists of an encoder and a decoder. The encoder transforms the input to a latent representation, often such that the latent representation is in a much smaller dimension than the input (Ballard, 1987). The decoder then maps the latent embedding to the reconstruction of X. An autoencoder is often used as a dimensional reduction technique to learn useful representations of data (Sakurada and Yairi, 2014).

While in a regular autoencoder the latent space is encoded and then decoded deterministically, i.e., there is no probabilistic modelling of the latent space, a variational autoencoder (VAE) learns a probability distribution in the latent space. VAE is often used as a generative model by sampling from the learnt latent space distribution and generating new samples that are similar in nature as the original data (Kingma and Welling, 2013). The assumption of VAE is that the distribution of data X, $P(X)$ is related to the distribution of the latent variable z, $P(z)$ by

$$P_\theta(X) = \int P_\theta(X|z)P(z)dz \qquad (2)$$

Here $P_\theta(X)$, also known as the marginal likelihood, is the probability of each data point in X under the entire generative process, parametrized by θ. The model aims to maximize $P_\theta(X)$ by optimizing the parameter θ so as to approximate the true distribution of data. In practice, $P_\theta(X|z)$ will be nearly zero for most z, and it is therefore more practical to learn a distribution $Q_\phi(z|X)$ which gives rise to z that is likely to produce X and then compute $P(X)$ from

$E_{z\sim Q_\phi}P(X|z)$. P_θ (X) and $E_{z\sim Q_\phi}P(X|z)$ can be shown to have the following relationship (Kingma and Welling, 2013):

$$log\, P_\theta(X) - D[Q_\phi(z|X)||P_\theta(z|X)] = E_{z\sim Q_\phi}[logP_\theta(X|z)] - D[Q_\phi(z|X)||P(z)] \quad (3)$$

The left hand side of equation (3) is the quantity we want to maximize, $\log P_\theta(X)$, plus an error term, which is the Kullback-Liebler divergence between the approximated posterior distribution $Q_\phi(z|X)$ and the true posterior distribution $P_\theta(z|X)$. The KL divergence is a measure of how one distribution is different from another one, and is always non-negative. Thus, maximizing the log likelihood log $P(X)$ can be achieved by maximizing the evidence lower bound (ELBO):

$$ELBO = \log P_\theta(X) - \mathcal{D}[Q_\phi(z|X)||P_\theta(z|X)] \quad (4)$$

The right hand side of equation (3) is something we can optimize by a gradient descent algorithm. $P_\theta(X|z)$ is modeled by the decoder network of the VAE parametrized by θ, and $Q_\phi(z|X)$ is modeled by the encoder network parametrized by ϕ. For continuous value inputs, $P_\theta(X|z)$ and $Q_\phi(z|X)$ are most commonly assumed to be Gaussian distributions (Ghosh, et al., 2019). $P(z)$ is a fixed prior distribution and assumed to be a standard multivariate normal distribution $\mathcal{N}(0, I)$. The first term $E_{z\sim Q_\phi}[logP_\theta(X|z)]$ is the expectation of the log probability of X given the encoder's output. Maximizing this term is equivalent to minimizing the reconstruction error of the autoencoder. The second term $D[Q_\phi(z|X)||P(z)]$ is the divergence between the approximated posterior distribution $Q_\phi(z|X)$ and the prior $P(z)$, and minimizing this term can be considered as adding a regularization term to prevent overfitting.

VAE is trained with the training data which follows a standard Gaussian distribution after z-score transformation. We impute missing values in the testing data with a trained VAE by an

iterative process. Initially, the missing values are replaced with random values sampled from a standard Gaussian distribution. Then the following sequence of steps are repeated until an empirically determined iteration threshold is reached: compute the latent variable z distribution given input X with the encoder; take the mean of latent variable distribution as the input to the decoder, and compute the distribution of reconstructed data $\hat{X}$; take the mean of the reconstructed data distribution as the reconstructed values; replace the missing values with reconstructed values and leave non-missing values unchanged. The testing data is scaled by the training data mean and variance before the imputation iterations, and inverse scaled after imputation.

Variational autoencoder imputation with shift correction

Regular implementation of VAE has an underlying assumption that the training data follows the same distribution of testing data. Below, we will discuss how to modify this assumption to better impute missing-not-at-random scenarios.

Since the VAE learns the data distribution from the training data, the output of imputation also follows the learnt distribution, which is similar to the training data. When the missing values are drawn from a different distribution than the training data, the imputation performance will drop due to the distribution shift. In the missing-not-at-random simulations where half of the lowest 10% values are masked, the missing values are considered to be shifted from the original training data to a smaller mean.

The lowest value missing scenario is a common type of missing values in biomedical data. When certain experimental conditions (e.g., low RNA sequencing depth) allow us to make assumptions that the majority of missing values are low expression values, we essentially have a prior knowledge that the distribution of missing values is shifted to the end of lower values. We

can therefore use VAE with the shift-correction implementation. Recall that in Equation (3), the underlying assumption is that the training data follows a Gaussian distribution X ~ $\mathcal{N}(\mu, \sigma)$, where μ and σ are the outputs of the decoder network which represent the mean and variance of the observed training data, as well as the missing data. When the lowest values are missing, the learnt distribution has larger mean than the actual missing data, causing the reconstructed $\hat{X}$ to have larger values. To correct this, we modify the assumption of training data distribution to follow $\mathcal{N}(\mu + \lambda\sigma, \sigma)$, where μ and σ are the outputs of the decoder network which represent the mean and variance of the missing data, and λ is a hyperparameter. The mean of the observed training data is then shifted to $\mu + \lambda\sigma$.

To test the lowest 10% missing case, we simulate a 10% lowest value missing scenario on the validation dataset, and select the shift correction parameter value that produces the smallest validation error. In reality, we may not know the actual range and amount of low value missing in the testing data and thus cannot simulate the situation on the validation data precisely. For a range of the lowest value missing scenarios, where half of the lowest 5%, 10%, 20%, and 30% values are missing respectively, we impute with a single λ which is selected based on the lowest 10% missing case. We thereby determine if it is possible to select λ without precise knowledge of the missing scenario on the testing data.

$\boldsymbol{\beta}$-VAE

β-VAE is a generalization of the variational autoencoder with a focus to discover interpretable factorized latent factors (Higgins, et al., 2017). A hyperparameter beta is introduced to the VAE loss to balance the reconstruction loss term with the regularization loss term. The loss of β-VAE is defined as:

$$L_{\beta-\mathrm{VAE}} = -E_{z\sim Q_\phi}[logP_\theta(X|z)] + \beta\mathcal{D}[Q_\phi(z|X)||P(z)] \quad (5)$$

where β is a hyperparameter.

β-VAE (β >1) has been shown to perform better than VAE in certain image generation tasks and has attracted increasing research interest (Burgess, et al., 2018). However, no prior work has investigated the effect of β on imputation. Since VAE can be considered as a special case of β-VAE, we extend our study to β-VAE with a varying β to further understand the effect of regularization on VAE imputation, and investigate potential possibility to increase its performance.

When β is 1, it is the same as VAE. When β>1, a stronger regularization is enforced, and the resulting latent space is smoother and more disentangled, which is a preferred property in certain learning tasks because more disentangled latent space has greater encoding efficiency (Higgins, et al., 2017).

On the other hand, when β=0, the regularization term is effectively removed. With the regularization term removed, the loss function only consists of the reconstruction loss term:

$$L_{\mathrm{VAE}'} = -E_{z\sim Q_\phi}[ogP_\theta(X|z)] \quad (6)$$

which resembles the reconstruction loss function of a simple autoencoder (AE) without any regularization, that can usually be expressed in the mean squared error between the input X and the reconstruction X' (Kramer, 1991) :

$$L(X, X') = ||X - X'||_2^2 \quad (7)$$

However, the loss of VAE without the regularization term as shown in equation (6) has a key difference from the loss of a simple autoencoder shown in equation (7). If (6) is viewed from a deterministic perspective, it is easy to distinguish the difference.

With the assumption that P_θ and $Q_\phi$are Gaussian distributions,

$$P_\theta\ (X|z) \sim N\ \left(X \middle| \mu_\theta(z),\ diag\big(\sigma_\theta(z)\big)\right),$$

$$Q_\phi\ (z|X) \sim N\ (z|\mu_\phi(X),\ diag\left(\sigma_\phi(X)\right))$$

the loss in (6) can be computed as the mean squared error between inputs and their mean reconstructions output by the decoder (Ghosh, et al., 2019):

$$L_{\mathrm{VAE}'} = \|X - \mu_\theta(z)\|_2^2 \qquad (8)$$

Unlike the deterministic reconstruction X' in equation (7), z in equation (8) is stochastic. However, the stochasticity of z can be relegated to a random variable that does not depend on ϕ, so that we can view (8) from a deterministic perspective. Using the reparameterization trick (Kingma and Welling, 2013), z can be represented by:

$$z = \mu_\phi(X) + \sigma_\phi(X) \odot \varepsilon, \qquad \varepsilon \sim \mathcal{N}(0, I) \qquad (9)$$

where $\odot$ is the element-wise product. Therefore the input to the decoder can be considered as the output of encoder $\mu_\phi(X)$ corrupted by a random gaussian noise ε multiplied by $\sigma_\phi(X)$. Consequently, the loss in (8) can be considered as the loss of a deterministic autoencoder, which but has noise injected to the latent space. In contrast, noise is not present in the deterministic regular AE loss in (7).

We perform three random missing experiments (5%, 10%, 30% missing) with β-VAE and vary the hyperparameter β from 0, 1, 4, to 10, to evaluate how β affects imputation accuracies. This will help us understand the VAE mechanism and how to use it in imputation.

Model parameters and hyper-parameters tuning

Model parameters and hyper-parameters tuning are conducted on the validation dataset. The latent dimension is usually several magnitude smaller than the input dimension in autoencoder implementations, but there is no golden rule to determine its size. We test three latent dimension sizes: 50, 200, 400. Furthermore, we test two architectures with three or five hidden layers. The hidden layers adjacent to the bottleneck layer has a 10-fold size increase, and each adjacent layer outwards after that has a constant size increase factor. For example, for a five hidden layer VAE with latent size 50, the hidden layer dimensions are 3000, 500, 50, 500, 3000, with input and output dimensions of 17176; for a three hidden layer VAE with latent size 200, the hidden layer dimensions are 2000, 200, 2000. We found that five hidden layers show slightly better performance than three hidden layers, and that latent dimensions of 200 and 400 produce similar performances, both better than 50. We therefore use a VAE with five hidden layers of dimensions of 6000, 2000, 200, 2000, 6000 in our subsequent experiments. ReLU function is used as the activation function on the hidden layers.

We use the ADAM optimizer and search for optimal learning rates on a grid of 1e-5, 5e-5, 1e-4, 5e-4. A learning rate of 5e-5 is selected after grid search. We find that model performance is not very sensitive to batch size, and use a batch size of 250 and training epochs of 250. The number of iterations to perform the iterative imputation is also determined empirically.

The imputed values are found to converge very quickly, and results remain mostly stable after 2 or 3 iterations. We use 3 as the iteration threshold.

Evaluation methods

To evaluate the VAE imputation framework, we compare it to other most commonly used missing value estimation methods: a K-nearest neighbor (KNN) method, and an iterative singular value decomposition (SVD) based method. We also construct a baseline using the mean value imputation method. KNN selects K number of samples which are most similar to the target sample with a missing gene based on Euclidean distance, and which all have values present in that gene. Imputation is a weighted average of the values of that gene in those K samples. We chose K=10 in our evaluations based on a study which reported that K in the range of 10-25 gave the best imputation results (Troyanskaya, et al., 2001). Next, the SVD method decomposes the data matrix to a linear combination of eigengenes and corresponding coefficients. Genes are regressed against L most significant eigengenes, during which process the missing genes are not used (Hastie, et al., 1999). The obtained coefficients are linearly multiplied by eigengenes to get a reconstruction with missing genes filled. This process is repeated until the total change in the matrix reaches a certain threshold. The reconstruction performance of SVD depends on the number of eigengenes selected for regression. We test a range of values and determine that the optimal performance is reached by full rank reconstruction. Hence we use full rank SVD in our evaluations. The mean value imputation method fills in the missing elements of each feature with the mean value of that feature across all non-missing samples.

We evaluate the root mean square error (RMSE) of the imputed data and uncorrupted ground truth,

$$RMSE = \frac{\sum_{i=1}^{n_{missing}} \sqrt{(x_i - x_i')^2}}{n_{missing}}$$

Where x_i is the ground truth of the masked value, and x_i' is the reconstructed value for the masked value.

To further evaluate the imputation effect on biomedical analysis, we compare the univariate correlation to clinical variables on the RNA sequencing data imputed by different methods. We conduct this analysis with the TCGA glioma cohort containing both LGG and GBM samples, and use two clinical variables: tumor histologic grade and survival time. The tumor grade and survival information for each brain tumor patients are publicly available (Ceccarelli, et al., 2016). The histologic grade variable in the TCGA brain tumor data contains three levels: Grade II, III and IV, indicating increasing level of tumor malignancy. We directly use the grade value as an ordinal variable of three levels, and calculate the Spearman correlation coefficient between each gene and the grade variable. The survival time is a continuous variable measured in months, and the vital status indicates if the patient was dead or alive when the study concluded. With this information, we perform a cox regression on each gene with respect to the survival outcome, and compute the univariate coefficient of each gene. A concordance index is computed between the coefficient obtained from the imputed data by each method and the coefficients obtained from the ground truth. A higher concordance index indicates better resemblance to the true data.

Results

RMSE of imputation on RNA sequencing data

We inspect the RMSEs in different simulated missing scenarios by different imputation methods. The significant scores are calculated using the Wilcoxon test with the “ggsignif” package in R. First, we evaluate the missing-completely-at-random cases at varying percentage 5%, 10%, and 30% random elements in the testing data were masked respectively, and models were compared on the reconstruction RMSE. VAE achieves better RMSEs than KNN in all tested missing scenarios, and reaches similar or better performances than SVD in most scenarios (Figure 1a).

In the first missing-not-at-random simulation case, the masked values are confined to certain genes which have the highest 10% GC content. Genes whose GC content are in the top 10% contain 50% random missing values in the testing data. VAE shows better reconstruction RMSE than KNN, and also achieves a slight advantage over SVD (Figure 1b). In the second case, 5% genes are masked entirely in the testing data. VAE again shows the best performance among competing methods (Figure 1c).

The final missing-not-at-random case is based on the gene expression values. The extreme values at the lowest 10% quantile are masked 50% randomly in the testing data. As a result, the observed values in the testing data shifts its distribution from the training data, and results in a decreased performance of imputation. However, with shift-correction implementation, VAE again achieves similar or better imputation accuracy than other methods (Figure 1d).

The shift correction is robust to a range of low percentage missing scenarios

We further investigate the robustness of the shift correction parameter against a range of missing percentage on the lowest values. The shift correction parameter is selected based on a

10% lowest value missing scenario simulated on the validation data. We use the same selected parameter to test on a range of missing scenarios, where half of the lowest 5%, 10%, 20%, and 30% values are missing respectively. All methods show worse prediction errors for smaller thresholds of missing values, because smaller thresholds indicate that the missing values are concentrated to smaller values, leading to a larger shifts in data distribution. We show that in these tested scenarios the shift correction VAE consistently achieves better result than KNN and SVD with the same λ (Figure 2). Therefore, λ selection does not need to exactly match the actual missing percentage, which is an advantage in real world implementations.

RMSE of imputation on DNA methylation data

For the imputation on DNA methylation data, the comparative performance of the KNN, SVD and VAE methods shows similar performance compared to the gene expression data. These three methods also show better performance than imputing with column mean. For missing-completely-at-random and block missing cases, VAE has similar performance as SVD, followed by KNN (Figure 3a, 3b). For the low coverage missing case, VAE achieves better RMSE than SVD and KNN (Figure 3c).

Correlation with clinical phenotypes

We investigate how closely the imputed data resembles the true data in terms of univariate correlation with respect to clinical variables. A higher concordance index between the correlation coefficients obtained from the imputed data and the coefficients obtained from the ground truth likely indicates the imputation method is better at preserving original data's univariate properties.

The ground truth of univariate Spearman correlations to histologic grade ranges from -1 to 1, with 46% of the genes having an absolute correlation value of 0.3 or greater. The majority of ground truth cox regression coefficients with respect to survival outcome is in the range of -5 and 5, with 72% of the genes having an absolute coefficient value of 0.3 or greater.

Table 2 contains the concordance indices from three imputation methods as well as a random imputation baseline. Random imputation is performed by filling the missing values by random sampling the training data distribution. It shows that VAE and SVD are similar, and VAE and SVD achieve better concordance indices than KNN for both grade and survival outcome correlations. This suggests that VAE and SVD imputed data likely has a better resemblance to true data in the context of biomedical analysis for molecular biologists interested in specific genes in the presence of missing values. Figure 4 illustrates pairwise difference between the coefficients obtained from the ground truth and the coefficients obtained from the imputed data by KNN and VAE respectively, and shows sharper peaks around zero for VAE in all cases for histology and in most cases for survival. The pairwise differences are mostly distributed around zero, and a smaller variance around the zero indicates the pairwise differences are smaller overall. In each missing scenario VAE has a smaller variance than KNN across ten trials (all p values <0.005).

Table 2. Correlation with clinical phenotypes (95% CI)

**(a)**	**KNN**	**VAE**	**SVD**	**Random**
**10% Random missing**	0.980±0.001	**0.982±0.001**	**0.982±0.001**	0.950±0.001
**Highest GC content missing**	0.949±0.002	**0.958±0.001**	**0.958±0.001**	0.816±0.006
**Entire genes missing**	0.918±0.005	0.932±0.004	**0.939±0.005**	0.500±0.004
**Lowest value missing**	0.977±0.001	0.983±0.001	**0.986±0.000**	0.906±0.007

(b)	KNN	VAE	SVD	Random
**10% Random missing**	0.969±0.002	**0.974±0.001**	**0.972±0.002**	0.873±0.050
**Highest GC content missing**	0.917±0.006	**0.931±0.004**	**0.933±0.006**	0.717±0.016
**Entire genes missing**	0.851±0.004	0.881±0.005	**0.906±0.006**	0.508±0.010
**Lowest value missing**	0.963±0.002	0.971±0.002	**0.976±0.002**	0.842±0.013

(a) Spearman correlation coefficient with tumor histologic grade; (b) Cox regression coefficient with survival outcome

Imputation time for new samples

The computation time for SVD or KNN to impute a single sample scales linearly with the dimension of the entire data matrix, while on the other hand, a VAE model can be pre-trained and applied directly to any given new sample to impute missing values. Once a VAE model is trained, the time to impute a new sample is almost negligible. VAE thus has the benefit of reducing computational cost especially at evaluation time.

Benchmark experiments are done on a 20 core cluster with Intel Xeon 2.40GHz CPUs, where the three methods are used to impute 100 samples in a gene expression matrix that consists of 6600 samples and 17176 genes. It takes an average of 2800 seconds to train the VAE network. In terms of evaluation time, the KNN method takes on average 8400 seconds, while SVD takes 36900 seconds, and VAE takes only 60 seconds, showing that VAE is several orders of magnitude faster at evaluation time.

$\boldsymbol{\beta}$-VAE and deterministic autoencoder

We perform three random missing experiments with β-VAE and vary the hyperparameter β from 0, 1, 4, to 10. Figure 5 shows that imputation results are similar for β=0 and β=1, while increasing β to larger values worsens the prediction accuracies.

The fact that β >1 produces worse imputation errors leads us to the hypothesis that the total loss of VAE (right hand side of equation (3)), consisting of the reconstruction loss and regularization loss, may be considered a tradeoff between reconstruction quality and latent space coding efficiency. If a greater emphasis is put on latent space regularization, the reconstruction quality suffers. We conclude that stronger regularization does not help VAE's imputation performance.

Furthermore, when β=0, the imputation performance is similar to vanilla VAE ($\beta = 1$). Therefore, for imputation, removing latent space regularization will not affect performance. From previous discussion in the β-VAE method section, the loss of β-VAE with β=0 looks similar to that of a simple AE, but the key difference is that noise is injected to the latent space for of β-VAE (β=0). We find that with a simple AE, the imputation iterations cannot converge and the resulting RMSE is very large (not shown because non-convergence). This suggests that the noise injection to the latent space enables the imputation ability of the VAE.

Conclusion

We have described a deep learning imputation framework for transcriptome and methylome data using a variational autoencoder (VAE). We implement a shift correction method to improve VAE imputation performance on a commonly encountered missing-not-at-random scenario. We demonstrate that the proposed framework is competitive with SVD, which is a time-inefficient method for real world scenarios. We also show that VAE outperforms KNN in

multiple scenarios such as bulk transcriptome and methylome data. VAE thus can be an important tool to analyze the large amounts of publicly available data from 1000s of studies that are publicly available in the Gene Expression omnibus (Barrett, et al., 2012).

We provide insights on the effect of latent space regularization on imputation performance. We show that increasing latent space regularization in the VAE implementation leads to larger error, and thus should be avoided in the imputation tasks. In addition, the regularization of latent space can be removed without affecting VAE's performance in imputation.

We also found that noise addition to the latent space largely helps VAE's good imputation performance, compared to a regular deterministic AE. The method of noise injection during training is reminiscent of denoising autoencoders (DAE). However, the noise addition for VAE and DAE are different. First, the noise in VAE depends on the input, whereas the DAE noise is independent of the input. Second, although noise addition to intermediate layers has been proposed in stacked denoising autoencoders for the purpose of representation learning (Vincent, et al., 2010), in most data imputation applications noise has only been added to the input layer of DAE (Costa, et al., 2018; Gondara and Wang, 2017). In contrast, noise is added to the latent space layer in VAE. It is not in the scope of this paper to evaluate how different noise addition schemes impact imputation and compare their performances. However, this may be worth exploring in future work.

Finally, in the context of imputing large dataset with high dimensional features, VAE has the potential benefit of reducing computational cost at evaluation time compared to SVD and KNN. This is because an autoencoder model can be pre-trained and applied directly to new samples, while SVD and KNN require computing the entire matrix each time a new sample is given.

In future work, it may be interesting to investigate VAE's application on single-cell RNA sequencing data, which has different missing scenarios than bulk RNA sequencing data. In addition, it may also be of interest to fully understand the effect of β in β-VAE when β is in the range from 0 to 1. Based on the hypothesis that there is a tradeoff between reconstruction quality and desired latent space property regulated by β, it can be expected that removing the regularization term (β=0) may even improve the vanilla VAE's (β=1) imputation performance. It is worth noting that such phenomenon did not occur, which invites further study.

Availability of Data and Materials

All data used in this manuscript are publicly available.

Gene expression data is version 2 of the adjusted pan-cancer gene expression data obtained from Synapse: https://www.synapse.org/#!Synapse:syn4976369.2. Clinical data of TCGA LGG/GBM can be found in the supplementary Table S1 in (Ceccarelli, et al., 2016). DNA methylation data is the WGBS data for BLUEPRINT methylomes (2016 release) obtained from rnbeads.org: https://www.rnbeads.org/methylomes.html.

Code is available at https://github.com/gevaertlab/BetaVAEImputation.

Declarations

Funding

Research reported in this publication was supported by the Fund for Innovation in Cancer Informatics (www.the-ici-fund.org), by the National Institute of Biomedical Imaging and Bioengineering of the National Institutes of Health (NIBIB https://www.nibib.nih.gov/), R01

EB020527 and R56 EB020527, and the National Cancer Institute

(NCI https://www.cancer.gov/), U01 CA217851 and U01 CA199241, all to OG. The content is solely the responsibility of the authors and does not necessarily represent the official views of the National Institutes of Health. The funders had no role in study design, data collection and analysis, decision to publish, or preparation of the manuscript.

Competing interests

The authors declare that they have no competing interests.

Figures

Figure 1. Imputation RMSE on the gene expression data for (a) missing-completely-at-random cases of 5%, 10% and 30%; (b) half of the highest 10% GC content genes missing case; (c) 5% genes entirely missing case; (d) half of the lowest 10% values missing case. The numbers above bars show the Wilcoxon test significant scores between VAE or VAE with shift correction and other methods.

Figure 2. RMSE with 95% confidence interval for simulations where half of the lowest 5%, 10%, 20%, and 30% values are missing respectively. VAE-shift-correction results are achieved using a single λ which is selected based on the lowest 10% missing case.

Figure 3. Imputation RMSE on the DNA methylation data for (a) missing-completely-at-random cases of 5%, 10% and 30%; (b) 5% genes entirely missing; (c) half of the coverage <6 CpG sites missing. The numbers above bars show the Wilcoxon test significant scores between VAE and other methods.

Figure 4. Pairwise difference between the coefficients obtained from the ground truth and the coefficients obtained from the imputed data by KNN and VAE: (a) Spearman correlation coefficients with histologic grade; (b) Regression coefficients with survival outcome.

Figure 5. Imputation RMSE error of β-VAE on 5%, 10% and 30% random missing of gene expression, with β = 0, 1, 4, and 10, denoting increasing strength of regularization.

References

Aghdam, R., *et al.* The Ability of Different Imputation Methods to Preserve the Significant Genes and Pathways in Cancer. *Genomics, Proteomics & Bioinformatics* 2017;15(6):396-404.

Arisdakessian, C., *et al.* DeepImpute: an accurate, fast, and scalable deep neural network method to impute single-cell RNA-seq data. *Genome biology* 2019;20(1):1-14.

Baghfalaki, T., Ganjali, M. and Berridge, D. Missing Value Imputation for RNA-Sequencing Data Using Statistical Models:

A Comparative Study. *Journal of Statistical Theory and Applications* 2016;15.

Ballard, D.H. Modular learning in neural networks. In, *Proceedings of the sixth National conference on Artificial intelligence - Volume 1*. Seattle, Washington: AAAI Press; 1987. p. 279-284.

Barrett, T., *et al.* NCBI GEO: archive for functional genomics data sets—update. *Nucleic acids research* 2012;41(D1):D991-D995.

Beaulieu-Jones, B.K. and Moore, J.H. Missing Data Imputation in the Electronic Health Record Using Deeply Learned Autoencoders. *Pac Symp Biocomput* 2017;22:207-218.

Burgess, C.P., *et al.* Understanding disentangling in $\beta $-VAE. *arXiv preprint arXiv:1804.03599* 2018.

Byron, S.A., *et al.* Translating RNA sequencing into clinical diagnostics: opportunities and challenges. *Nat Rev Genet* 2016;17(5):257-271.

Campbell, J.D., *et al.* Genomic, pathway network, and immunologic features distinguishing squamous carcinomas. *Cell reports* 2018;23(1):194-212. e196.

Ceccarelli, M., *et al.* Molecular profiling reveals biologically discrete subsets and pathways of progression in diffuse glioma. *Cell* 2016;164(3):550-563.

Champion, M., *et al.* Module Analysis Captures Pancancer Genetically and Epigenetically Deregulated Cancer Driver Genes for Smoking and Antiviral Response. *EBioMedicine* 2018;27:156-166.

Chen, C.L., *et al.* Deep Learning in Label-free Cell Classification. *Scientific Reports* 2016;6:21471.

Chen, Y., *et al.* Gene expression inference with deep learning. *Bioinformatics* 2016;32(12):1832-1839.

Chen, Y.C., *et al.* Effects of GC bias in next-generation-sequencing data on de novo genome assembly. *PLoS One* 2013;8(4):e62856.

Conesa, A., *et al.* A survey of best practices for RNA-seq data analysis. *Genome Biol* 2016;17:13.

Costa, A.F., *et al.* Missing data imputation via denoising autoencoders: the untold story. In, *International Symposium on Intelligent Data Analysis*. Springer; 2018. p. 87-98.

Eraslan, G., *et al.* Single-cell RNA-seq denoising using a deep count autoencoder. *Nature communications* 2019;10(1):390.

Faisal, S. and Tutz, G. Missing value imputation for gene expression data by tailored nearest neighbors. *Stat Appl Genet Mol Biol* 2017;16(2):95-106.

Garciarena, U. and Santana, R. An extensive analysis of the interaction between missing data types, imputation methods, and supervised classifiers. *Expert Systems with Applications* 2017;89:52-65.

Gevaert, O., Tibshirani, R. and Plevritis, S.K. Pancancer analysis of DNA methylation-driven genes using MethylMix. *Genome biology* 2015;16(1):17.

Gevaert, O., Tibshirani, R. and Plevritis, S.K. Pancancer analysis of DNA methylation-driven genes using MethylMix. *Genome Biol* 2015;16:17.

Ghosh, P.*, et al.* From variational to deterministic autoencoders. *arXiv preprint arXiv:1903.12436* 2019.

Gondara, L. and Wang, K. Multiple imputation using deep denoising autoencoders. *arXiv preprint arXiv:1705.02737* 2017.

Grønbech, C.H.*, et al.* scVAE: Variational auto-encoders for single-cell gene expression data. *bioRxiv* 2018:318295.

Hastie, T.*, et al.* Imputingmissing data for gene expression arrays. 1999.

Higgins, I.*, et al.* beta-VAE: Learning Basic Visual Concepts with a Constrained Variational Framework. *ICLR* 2017;2(5):6.

Hu, Z.*, et al.* Toward controlled generation of text. In, *Proceedings of the 34th International Conference on Machine Learning-Volume 70*. JMLR. org; 2017. p. 1587-1596.

Jaques, N.*, et al.* Multimodal autoencoder: A deep learning approach to filling in missing sensor data and enabling better mood prediction. 2018.

Kingma, D.P. and Welling, M. Auto-encoding variational bayes. *arXiv preprint arXiv:1312.6114* 2013.

Kramer, M.A. Nonlinear principal component analysis using autoassociative neural networks. *AIChE journal* 1991;37(2):233-243.

Kulis, M. and Esteller, M. DNA methylation and cancer. *Adv Genet* 2010;70:27-56.

Leung, M.K., *et al.* Deep learning of the tissue-regulated splicing code. *Bioinformatics* 2014;30(12):i121-129.

Libbrecht, M.W. and Noble, W.S. Machine learning applications in genetics and genomics. *Nat Rev Genet* 2015;16(6):321-332.

Litovkin, K., *et al.* DNA Methylation-Guided Prediction of Clinical Failure in High-Risk Prostate Cancer. *PLoS One* 2015;10(6):e0130651.

Little, R.J. and Rubin, D.B. Statistical analysis with missing data. John Wiley & Sons; 2019.

Lopez, R., *et al.* Deep generative modeling for single-cell transcriptomics. *Nature methods* 2018;15(12):1053.

Malta, T.M., *et al.* Machine learning identifies stemness features associated with oncogenic dedifferentiation. *Cell* 2018;173(2):338-354. e315.

Mattei, P.-A. and Frellsen, J. MIWAE: Deep Generative Modelling and Imputation of Incomplete Data Sets. In, *International Conference on Machine Learning*. 2019. p. 4413-4423.

McCoy, J.T., Kroon, S. and Auret, L. Variational Autoencoders for Missing Data Imputation with Application to a Simulated Milling Circuit. *IFAC-PapersOnLine* 2018;51(21):141-146.

Min, S., Lee, B. and Yoon, S. Deep learning in bioinformatics. *Brief Bioinform* 2017;18(5):851-869.

Moorthy, K., *et al.* Missing-values imputation algorithms for microarray gene expression data. In, *Microarray Bioinformatics*. Springer; 2019. p. 255-266.

Sakurada, M. and Yairi, T. Anomaly Detection Using Autoencoders with Nonlinear Dimensionality Reduction. In, *Proceedings of the MLSDA 2014 2nd Workshop on Machine Learning for Sensory Data Analysis*. Gold Coast, Australia QLD, Australia: ACM; 2014. p. 4-11.

Smaragdis, P., Raj, B. and Shashanka, M. Missing Data Imputation for Time-Frequency Representations of Audio Signals. *Journal of Signal Processing Systems* 2011;65(3):361-370.

Stunnenberg, H.G., *et al.* The International Human Epigenome Consortium: a blueprint for scientific collaboration and discovery. *Cell* 2016;167(5):1145-1149.

Tomczak, K., Czerwinska, P. and Wiznerowicz, M. The Cancer Genome Atlas (TCGA): an immeasurable source of knowledge. *Contemp Oncol (Pozn)* 2015;19(1A):A68-77.

Troyanskaya, O., *et al.* Missing value estimation methods for DNA microarrays. *Bioinformatics* 2001;17(6):520-525.

Vincent, P., *et al.* Extracting and composing robust features with denoising autoencoders. In, *Proceedings of the 25th international conference on Machine learning*. Helsinki, Finland: ACM; 2008. p. 1096-1103.

Vincent, P., *et al.* Extracting and composing robust features with denoising autoencoders. In, *Proceedings of the 25th international conference on Machine learning*. ACM; 2008. p. 1096-1103.

Vincent, P., *et al.* Stacked Denoising Autoencoders: Learning Useful Representations in a Deep Network with a Local Denoising Criterion. *J. Mach. Learn. Res.* 2010;11:3371-3408.

Way, G.P. and Greene, C.S. Extracting a biologically relevant latent space from cancer transcriptomes with variational autoencoders. *BioRxiv* 2017:174474.

Wheeler, D.L., *et al.* Database resources of the national center for biotechnology information. *Nucleic acids research* 2006;35(suppl_1):D5-D12.

Wulsin, D.F., *et al.* Modeling electroencephalography waveforms with semi-supervised deep belief nets: fast classification and anomaly measurement. *J Neural Eng* 2011;8(3):036015.

Yeh, R.A., *et al.* Semantic image inpainting with deep generative models. In, *Proceedings of the IEEE Conference on Computer Vision and Pattern Recognition*. 2017. p. 5485-5493.

Yu, T., Peng, H. and Sun, W. Incorporating nonlinear relationships in microarray missing value imputation. *IEEE/ACM Transactions on Computational Biology and Bioinformatics* 2010;8(3):723-731.

Zheng, H., *et al.* Benchmark of long non-coding RNA quantification for RNA sequencing of cancer samples. *GigaScience* 2019;8(12):giz145.

Figure

Click here to access/download;Figure;Figure1.png

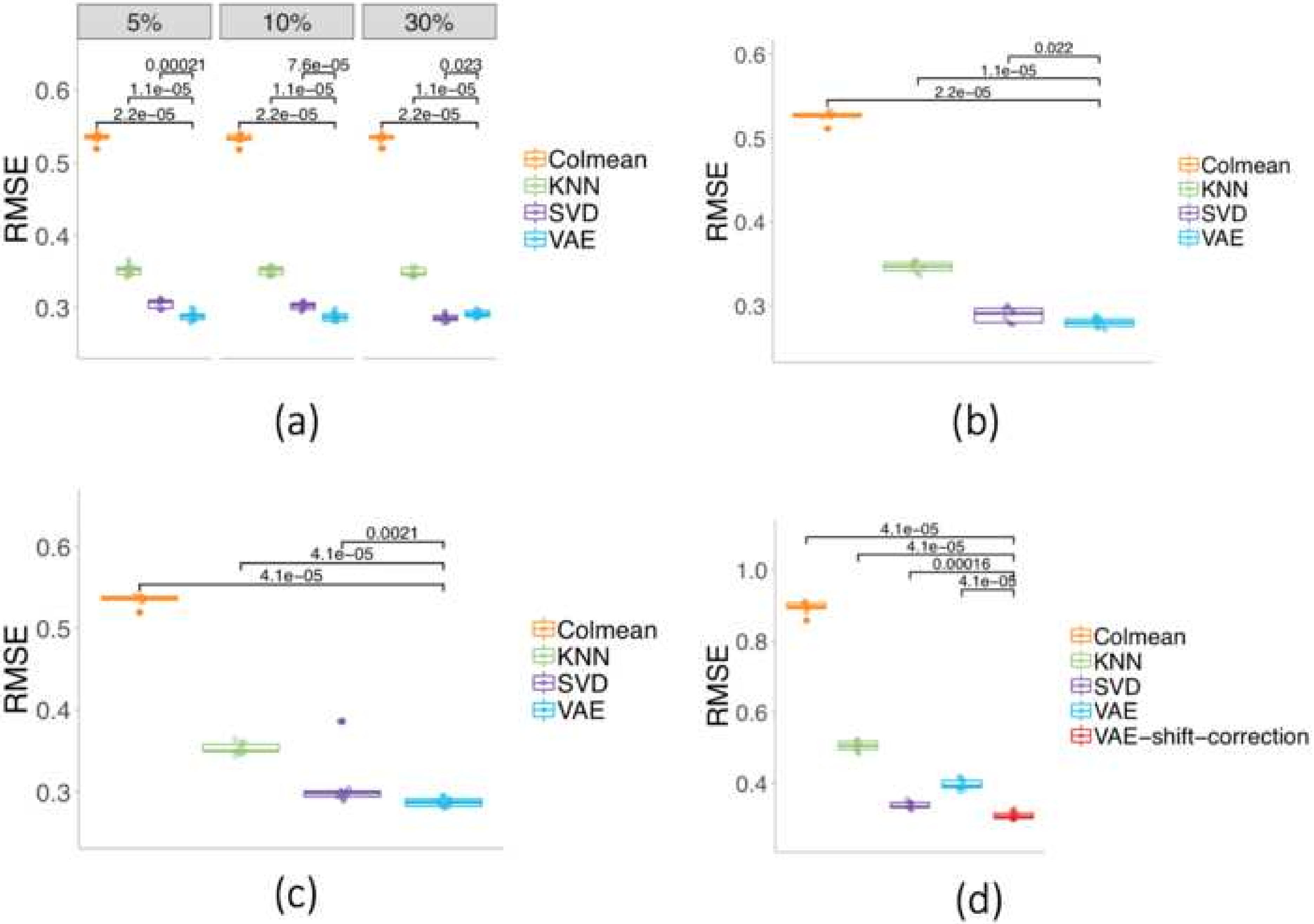

 Click here to access/download;Figure;Figure2.png

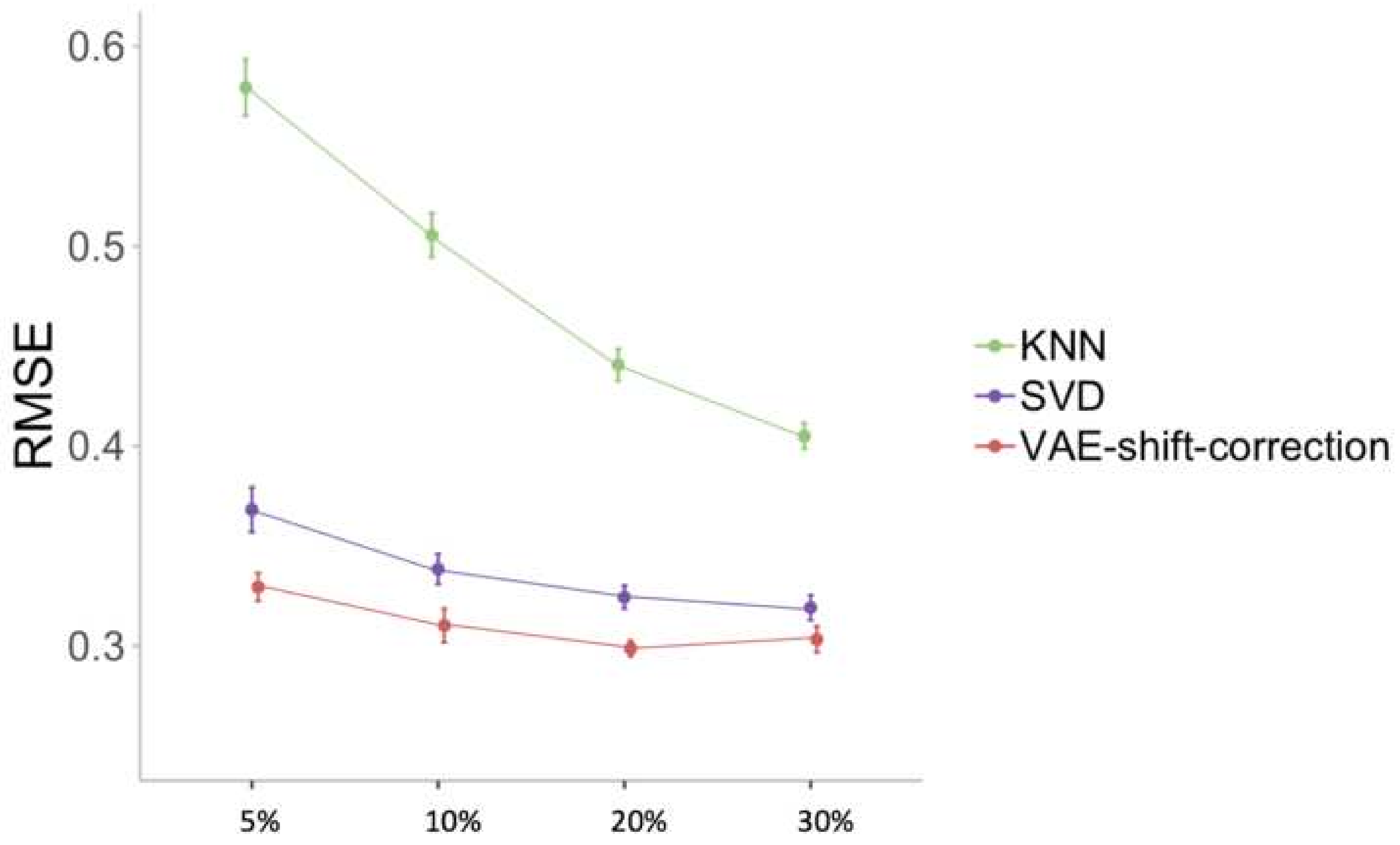

Click here to access/download;Figure;Figure3.png

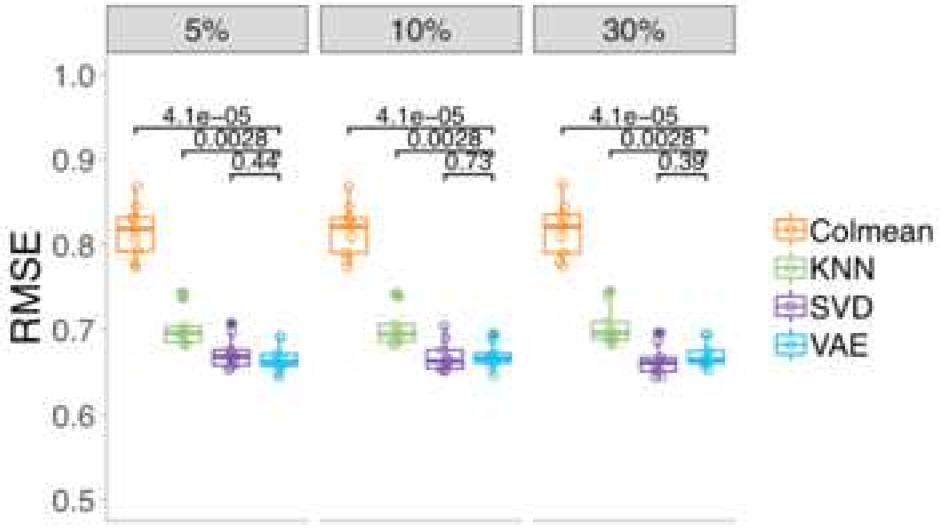

(a)

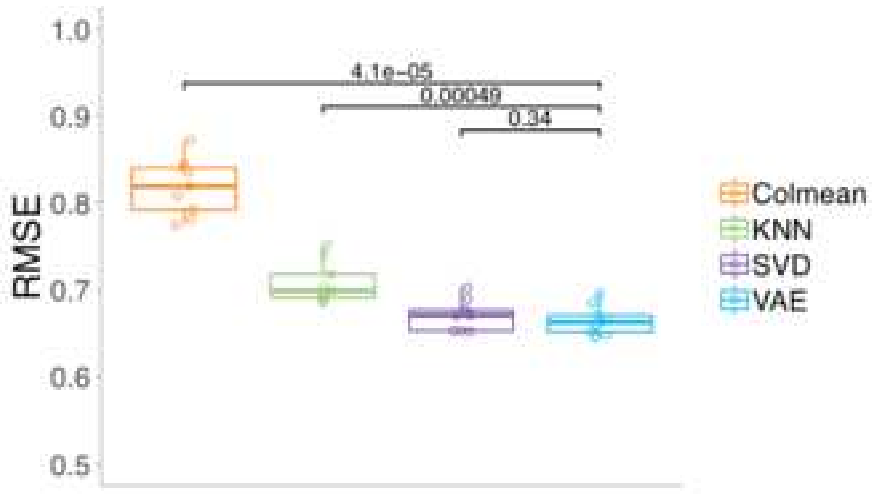

(b)

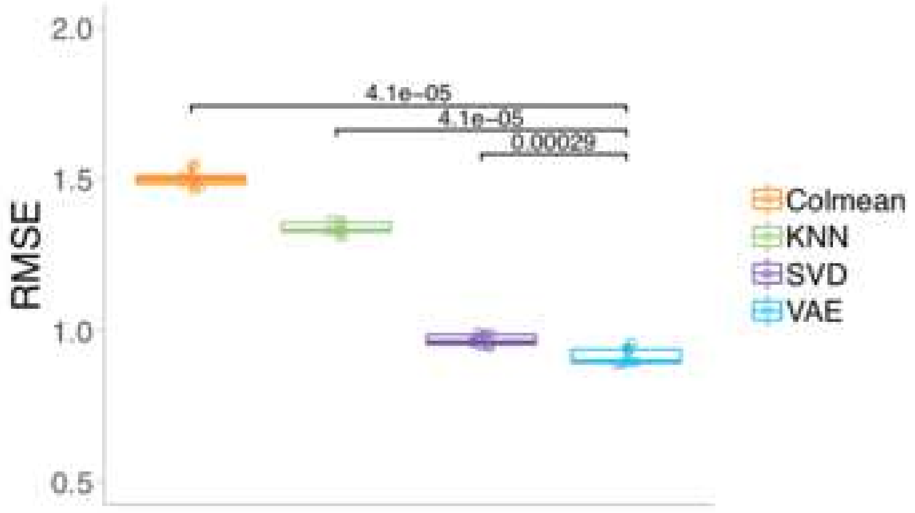

(c)

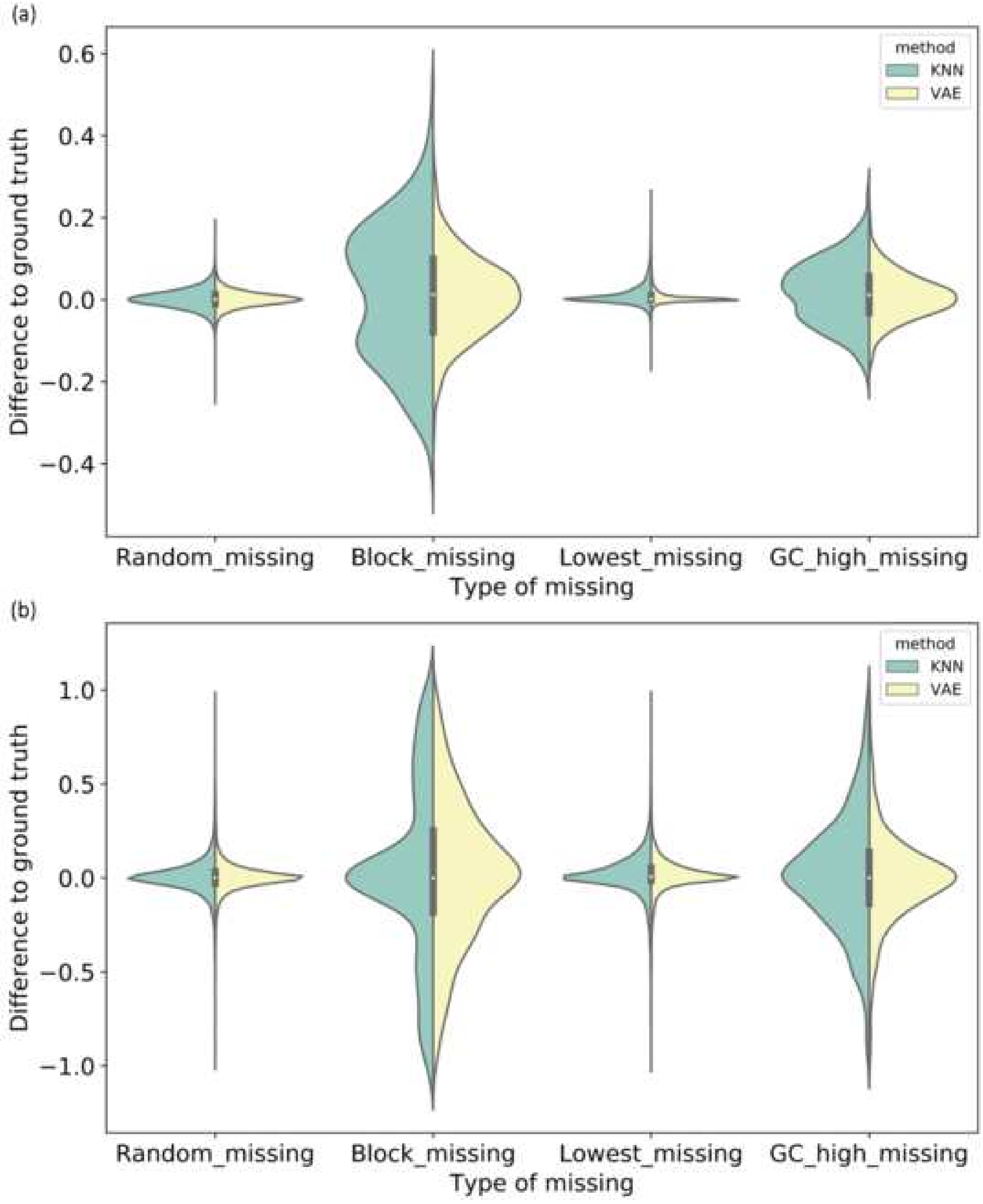
(a)
method
KNN
VAE
0.6
0.4
0.2
0.0
−0.2
−0.4
Difference to ground truth
Random_missing
Block_missing
Lowest_missing
GC_high_missing
Type of missing
(b)
method
KNN
VAE
1.0
0.5
0.0
−0.5
−1.0
Difference to ground truth
Random_missing
Block_missing
Lowest_missing
GC_high_missing
Type of missing

Figure

[Click here to access/download;Figure;Figure5.png]

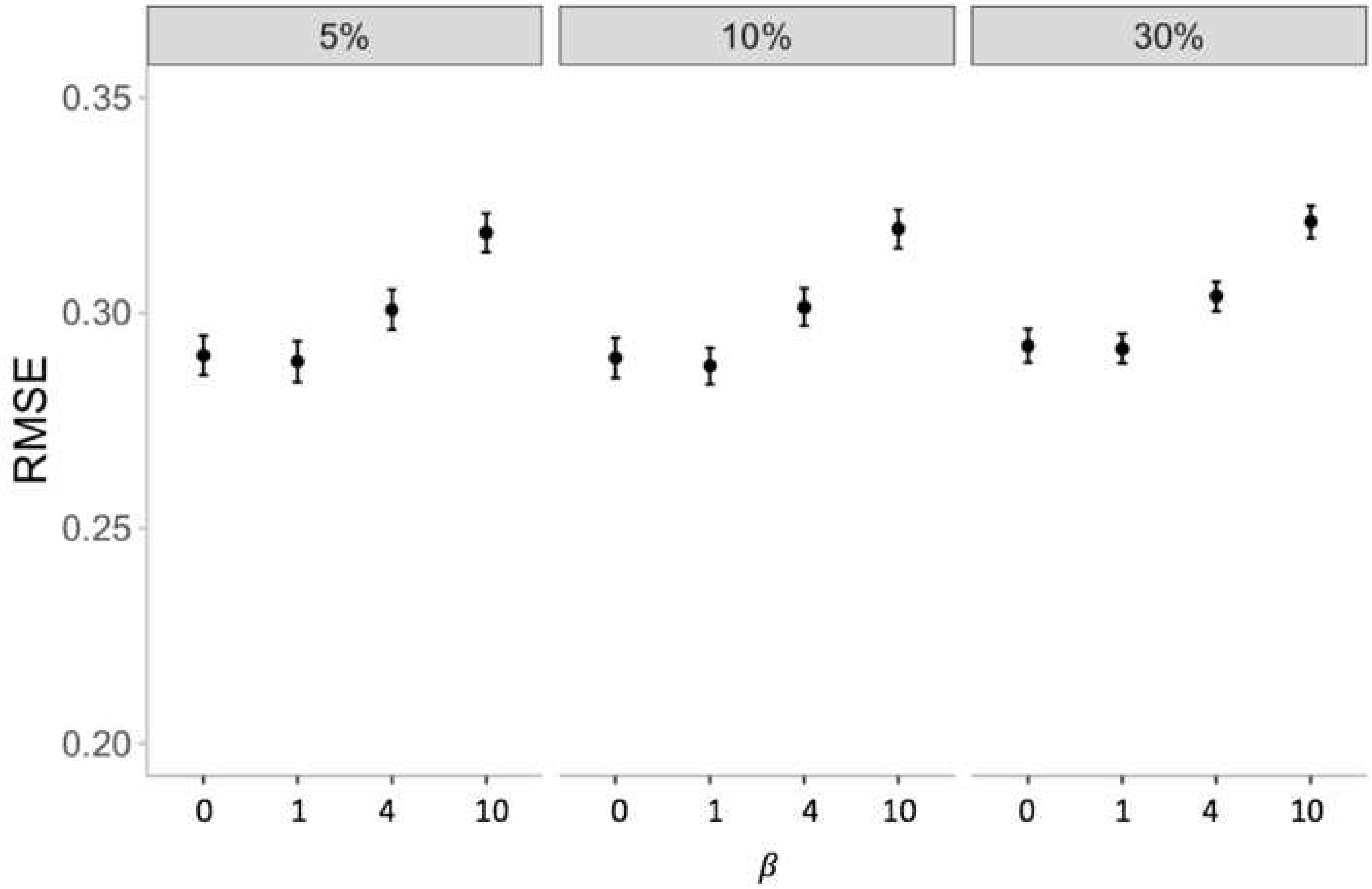

Supplement: giaa082_GIGA-D-20-00059_Revision_1 [file giaa082_giga-d-20-00059_revision_1.pdf]
