# Supplementary material for: Genomic data imputation with variational auto-encoders
Source: Gigascience. 2020 Aug 6;9(8):giaa082. doi: 10.1093/gigascience/giaa082 (PMC7407276; doi:10.1093/gigascience/giaa082)
Supplement: giaa082_GIGA-D-20-00059_Revision_3 [file giaa082_giga-d-20-00059_revision_3.pdf]

GigaScience
Genomic data imputation with variational autoencoders
--Manuscript Draft--

**Manuscript Number:**	GIGA-D-20-00059R3
**Full Title:**	Genomic data imputation with variational autoencoders
**Article Type:**	Technical Note
**Funding Information:**	National Institute of Biomedical Imaging and Bioengineering (R01 EB020527) — Mr. Olivier Gevaert<br>National Institute of Biomedical Imaging and Bioengineering (R56 EB020527) — Mr. Olivier Gevaert<br>National Cancer Institute (US) (U01 CA217851) — Mr. Olivier Gevaert<br>National Cancer Institute (U01 CA199241) — Mr. Olivier Gevaert
**Abstract:**	Background: As missing values are frequently present in genomic data, practical methods to handle missing data are necessary for downstream analyses that require complete datasets. State-of-the-art imputation techniques including Singular Value Decomposition (SVD) and K-Nearest Neighbors (KNN) based methods can be computationally expensive for large datasets and it is difficult to modify these algorithms to handle certain missing-not-at-random cases.<br>Findings: In this work, we use a deep learning framework based on the variational autoencoder (VAE) for genomic missing value imputation and demonstrate its effectiveness in transcriptome and methylome data analysis. We show that in the vast majority of our testing scenarios, VAE achieves similar or better performances than the most widely used imputation standards, while having computational advantage at evaluation time. When dealing with missing-not-at-random, e.g. low values are missing, we develop simple yet effective methodologies to leverage the prior knowledge about missing data. Furthermore, we investigate the effect of varying latent space regularization strength in VAE on the imputation performances, and in this context show why VAE has a better imputation capacity compared to a regular deterministic autoencoder (AE).<br>Conclusions: We describe a deep learning imputation framework for transcriptome and methylome data using a variational autoencoder and show that it can be a preferable alternative to traditional methods for data imputation, especially in the setting of large-scale data and certain missing-not-at-random scenarios.
**Corresponding Author:**	Olivier Gevaert<br><br>UNITED STATES
**Corresponding Author Secondary Information:**	
**Corresponding Author's Institution:**	
**Corresponding Author's Secondary Institution:**	
**First Author:**	Yeping Lina Qiu
**First Author Secondary Information:**	
**Order of Authors:**	Yeping Lina Qiu<br>Hong Zheng<br>Olivier Gevaert
**Order of Authors Secondary Information:**	
**Response to Reviewers:**	We have addressed the reviewer's final comment and the editorial comments in this revision. We thank the editor and reviewers' great effort in helping to improve the

Powered by Editorial Manager® and ProduXion Manager® from Aries Systems Corporation

	quality of this manuscript.
Additional Information:	
Question	Response
Are you submitting this manuscript to a special series or article collection?	No
**Experimental design and statistics**<br><br>Full details of the experimental design and statistical methods used should be given in the Methods section, as detailed in our Minimum Standards Reporting Checklist. Information essential to interpreting the data presented should be made available in the figure legends.<br><br>Have you included all the information requested in your manuscript?	Yes
**Resources**<br><br>A description of all resources used, including antibodies, cell lines, animals and software tools, with enough information to allow them to be uniquely identified, should be included in the Methods section. Authors are strongly encouraged to cite Research Resource Identifiers (RRIDs) for antibodies, model organisms and tools, where possible.<br><br>Have you included the information requested as detailed in our Minimum Standards Reporting Checklist?	Yes
**Availability of data and materials**<br><br>All datasets and code on which the conclusions of the paper rely must be either included in your submission or deposited in publicly available repositories (where available and ethically appropriate), referencing such data using a unique identifier in the references and in the "Availability of Data and Materials" section of your manuscript.	Yes

Have you have met the above requirement as detailed in our Minimum Standards Reporting Checklist?	

Manuscript

Click here to access/download;Manuscript;Gevaert_manuscript_for_productio

Genomic data imputation with variational autoencoders

Yeping Lina Qiu[1,2], Hong Zheng[1], Olivier Gevaert[1,3,*]

[1] Stanford Center for Biomedical Informatics Research (BMIR), Department of Medicine, Stanford University, Stanford, CA USA

[2] Department of Electrical Engineering, Stanford University, Stanford, CA, USA

[3] Department of Biomedical Data Science, Stanford University, Stanford, CA, USA

* To whom correspondence should be addressed: ogevaert@stanford.edu, ORCID: https://orcid.org/0000-0002-9965-5466

Abstract

Background: As missing values are frequently present in genomic data, practical methods to handle missing data are necessary for downstream analyses that require complete datasets. State-of-the-art imputation techniques including Singular Value Decomposition (SVD) and K-Nearest Neighbors (KNN) based methods can be computationally expensive for large datasets and it is difficult to modify these algorithms to handle certain missing-not-at-random cases.

Findings: In this work, we use a deep learning framework based on the variational autoencoder (VAE) for genomic missing value imputation and demonstrate its effectiveness in transcriptome and methylome data analysis. We show that in the vast majority of our testing scenarios, VAE achieves similar or better performances than the most widely used imputation standards, while having computational advantage at evaluation time. When dealing with missing-not-at-random, e.g. low values are missing, we develop simple yet effective methodologies to leverage the prior knowledge about missing data. Furthermore, we investigate the effect of varying latent space regularization strength in VAE on the imputation performances, and in this context show why VAE has a better imputation capacity compared to a regular deterministic autoencoder (AE).

Conclusions: We describe a deep learning imputation framework for transcriptome and methylome data using a variational autoencoder and show that it can be a preferable alternative to traditional methods for data imputation, especially in the setting of large-scale data and certain missing-not-at-random scenarios.

Keywords: imputation, variational autoencoder, deep learning

Introduction

The massive and diverse datasets in genomics have provided researchers a rich resource to study the molecular basis of diseases. The profiling of gene expression and DNA methylation have enabled the identification of cancer driver genes or biomarkers [1-6]. Many such studies on cancer genomics require complete datasets [7]. However, missing values are frequently present in these data due to various reasons including low resolution, missing probes, and artifacts [8, 9]. Therefore, practical methods to handle missing data in genomic datasets are needed for effective downstream analyses.

One way to complete the data matrices is to ignore missing values by removing` the entire feature if any of the samples has a missing value in that feature, but this is usually not a good strategy as the feature may contain useful information for other samples. The most preferable way to handle missing data is to impute their values in the pre-processing step. Many approaches have been proposed for this purpose [10], including replacement using average values, estimation using weighted K-nearest neighbor (KNN) method [11, 12], and estimation using singular value decomposition (SVD) based methods [11]. KNN and SVD are two techniques that have been commonly used as benchmarks against new developments [13, 14]. KNN imputes missing value of a feature in a given sample with the weighted average of the feature values in a number of similar samples, as calculated by some distance measure. SVD attempts to estimate data structure from the entire input including the samples with missing values, and fill in the missing values iteratively according to the global structure. For this reason, SVD is inefficient on large matrices in practice since new decompositions have to be estimated for each missing sample, which is a very time-consuming process. However, SVD serves as an

important benchmarking method to determine how well other, faster methods perform compared to SVD.

In recent years, a branch of machine learning which emerged based on big data and deep artificial neural network architectures, usually referred to as deep learning, has advanced rapidly and shown great potential for applications in bioinformatics [15]. Deep learning has been applied in areas including genomics studies [16-18], biomedical imaging [19], and biomedical signal processing [20]. Autoencoders (AE) are a deep learning based model which form the basis of various frameworks for missing value imputation, and they have shown promising results for genomic data, imaging data and industrial data applications [21-26]. However, a simple AE without regularization is rarely ranked among the competitors for data imputation [27, 28]. When a simple AE only focuses on creating output close to the input without any constraints, the model may overfit on the training data instead of learning the latent structure, such as dependencies and regularities characteristic of the data distribution [29], which makes it unlikely to impute well given new samples. Denoising autoencoder (DAE) is a type of autoencoder that specifically uses noise corruption to the input to create robust latent features [29]. DAE has been extensively used in the application of data imputation [23, 27]. The corrupting noise introduced in the DAE can be in many different forms, such as masking noise, Gaussian noise, and salt-and-pepper noise [30].

Variational autoencoders (VAE) are a probabilistic autoencoder that has wide applications in image and text generation [31-33]. VAE learns the distributions of latent space variables that make the model generate output similar to the input. VAE has primarily been used as a powerful generative tool, having the ability to produce realistic fake contents in images, sound signal or texts, that highly resemble the real life contents that they learn from. The generative power is made possible by regularizing the latent space [33]. Constraining the latent

space distributions to be close to a standard Gaussian helps to achieve a smooth latent space where two close points in the latent space should lead to similar reconstructions, and any point sampled from the latent space should give a meaningful reconstruction [34]. VAE has been applied in genomic contexts such as latent space learning of gene expression data [35]. In addition, recent works have applied VAE on single cell RNA sequencing data for clustering, batch correction and differential expression analysis [36, 37]. However, VAE has not been extensively studied for genomic data imputation for bulk RNA expression and DNA methylation data, while large amounts of retrospective genomic and epigenomic data are available through databases like the Gene Expression Omnibus (GEO) [38] and the Short Read Archive (SRA)[39].

Here, we examine the VAE mechanism and its application to genomic missing value imputation with bulk transcriptome and methylome data. We show that for both missing-completely-at-random and missing-not-at-random cases in transcriptome data and methylome data, VAE achieves similar or better performances than the de facto standards, and thus is a strong alternative to traditional methods for data imputation [40]. We demonstrate that in a missing-not-at-random scenario where the missing data distribution is not the same as the seen data, a shift correction method can be implemented to improve VAE's extrapolation performance. Furthermore, we investigate the effect of latent space regularization on imputation with a generalization of the variational autoencoder - β-VAE [41]. In the context of β-VAE results, we provide insights on why VAE can achieve good imputation performance compared to a regular deterministic AE.

Materials and Methods

Datasets

We use two datasets to perform data imputation: pan-cancer RNA sequencing data from The Cancer Genome Atlas (TCGA) datasets [2, 42, 43], and DNA methylation data [44-47]. Both datasets contain only numeric values. The RNA sequencing data is expressed in RPKM (Reads Per Kilobase of transcript, per Million mapped reads), which is a normalized unit of transcript expression. The DNA methylation data is obtained from bisulfite sequencing, and it contains the numeric values of the methylation level at each CpG site. The RNA sequencing data has a feature dimension of 20531 genes. There are 15% of the genes containing more or less NA values, while the rest of the 85% of the genes are complete. Within the 15% of the genes who have missing values, on average 8.5% of the values are missing. The NA values are introduced in the Synapse pre-processing pipeline, where genes with mostly zero reads or with residual batch effects after batch correction were removed from the adjusted samples and replaced with NAs. In order to have a ground truth to evaluate the missing value imputation frameworks, we remove the 15% genes with NA values in our pre-processing, which results in a feature dimension of 17176 genes. We then normalize the data by log transformation and z-score transformation. We use 667 glioma patient samples, including glioblastoma (GBM) and low-grade-glioma (LGG), to train and test the missing value imputation framework. In pre-processing the DNA methylation data, we remove the NA values, and normalize the data by negative log transformation and z-score transformation. We use the smallest chromosome subset (Chromosome 22) so that the resulting data dimension is not prohibitive for benchmarking different computation methods. The resulting data has 21220 CpG sites and 206 samples.

Missing data simulations

Each dataset is split into 80%-20% for training and hold-out testing. The training dataset is further split 80%-20%, where 20% is the validation dataset for hyper-parameter tuning. After hyper-parameters are selected, the entire training set is used for training. The sample split for the RNA sequencing dataset is stratified by the glioma subtypes (LGG versus GBM), and the split is random for the DNA methylation data since the samples are homogenous. The training data is a complete dataset without missing values. Missing values are introduced to the testing data in two forms: missing-completely-at-random (MCAR) and missing-not-at-random (MNAR) [48] (Table 1).

In the MCAR cases, we randomly mask a number of elements in each row by replacing the original values with NAs. To test a range of missing severity, we make the number of masked elements amount to 5%, 10%, and 30% of the total number of elements respectively.

Each of the MNAR simulation is motivated by a different real world condition specific to either gene expression data or methylation data. For the gene expression data, we simulate three MNAR scenarios, each of which has 5% of the total data values missing. In the first scenario, the masked values are concentrated at certain genes. Such genes are selected based on their GC content, which is the percentage of nitrogenous bases on a RNA fragment that are either guanine (G) or cytosine (C). Too high or too low GC content influences RNA sequencing coverage, and potentially results in missing values from these genes [49]. We select genes with GC-content at the highest 10% and randomly mask half of these values. In the second simulation case, certain genes are masked entirely. In some pre-processing pipelines of RNA sequencing data, genes with residual batch effects after batch correction are replaced with NAs in the adjusted samples. Such pre-processing may give rise to the MNAR case where some genes are entirely missing in some samples. We randomly select 5% of the genes and mask all values from these genes in the testing

data, and as a result, the corrupted data miss all values for specific genes. The third scenario is based on gene expression level. When the RNA sequencing depth is relatively low, it is relatively easy to miss genes that have low expression levels, because the reads, generated from those genes, are too few to be captured during sequencing [50]. Therefore, we consider a possible scenario where lowly expressed genes are prone to be missing. In the testing data we first choose gene expression values at the lowest 10% quantile, and then randomly mask half of these values.

For the DNA methylation data, we simulate two MNAR scenarios. The first scenario is complete missing of certain CpG sites, which is similar to the second MNAR case in gene expression data, where we select 5% of the features and mask them entirely in the testing data. In the second case, we mask CpG sites that have fewer coverage than a certain threshold. Some CpG sites may have very few reads mapped to them, which undermines the confidence in the measurement of methylation level. Thus, we choose an arbitrary coverage threshold of six reads for the methylation status of a CpG site to be confidently determined. Methylation levels of CpGs with less than six reads mapped to them are treated as missing values in the analysis here.

For each simulation scenario described above, we create ten random trials to measure the average imputation performance. The uncorrupted testing data is used to compute the imputation RMSE.

Table 1. Simulation experiments on RNA sequencing data and DNA methylation data

**Data**	**Missing type**	**Missing scenario**
RNA sequencing data	MCAR	5% completely random missing
		10% completely random missing

		30% completely random missing
	MNAR	50% random missing in genes with the highest 10% GC content
		5% genes are entirely missing
		50% random missing in genes with the lowest 10% expression level
DNA methylation data	MCAR	5% completely random missing
		10% completely random missing
		30% completely random missing
	MNAR	5% CpG sites are entirely missing
		50% random missing in CpG sites with coverage lower than 6 reads

Variational autoencoder

An autoencoder is an unsupervised deep neural network that is trained to reconstruct an input X by learning a function $h_{w,b}(X) \approx X$. This is done by minimizing the loss function between the input X and the network's output X': $L(X, X')$. The most common loss function is the root mean squared error:

$$L(X, X') = \sqrt{||X - X'||^2} \qquad (1)$$

An autoencoder consists of an encoder and a decoder. The encoder transforms the input to a latent representation, often such that the latent representation is in a much smaller dimension than the input [51]. The decoder then maps the latent embedding to the reconstruction of X. An autoencoder is often used as a dimensional reduction technique to learn useful representations of data [52].

While in a regular autoencoder the latent space is encoded and then decoded deterministically, i.e., there is no probabilistic modelling of the latent space, a variational autoencoder (VAE) learns a probability distribution in the latent space. VAE is often used as a generative model by sampling from the learnt latent space distribution and generating new samples that are similar in nature as the original data [33]. The assumption of VAE is that the distribution of data X, $P(X)$ is related to the distribution of the latent variable z, $P(z)$ by

$$P_\theta(X) = \int P_\theta(X|z)P(z)dz \qquad (2)$$

Here $P_\theta(X)$, also known as the marginal likelihood, is the probability of each data point in X under the entire generative process, parametrized by θ. The model aims to maximize $P_\theta(X)$ by optimizing the parameter θ so as to approximate the true distribution of data. In practice, $P_\theta(X|z)$ will be nearly zero for most z, and it is therefore more practical to learn a distribution $Q_\phi(z|X)$ which gives rise to z that is likely to produce X and then compute $P(X)$ from $E_{z\sim Q_\phi}P(X|z)$. P_θ (X) and $E_{z\sim Q_\phi}P(X|z)$ can be shown to have the following relationship [33]:

$$log\, P_\theta(X) - D[Q_\phi(z|X)||P_\theta(z|X)] = E_{z\sim Q_\phi}[logP_\theta(X|z)] - D[Q_\phi(z|X)||P(z)] \qquad (3)$$

The left hand side of equation (3) is the quantity we want to maximize, $\log P_\theta(X)$, plus an error term, which is the Kullback-Liebler divergence between the approximated posterior distribution $Q_\phi(z|X)$ and the true posterior distribution $P_\theta(z|X)$. The KL divergence is a measure of how one distribution is different from another one, and is always non-negative. Thus, maximizing the log likelihood log $P(X)$ can be achieved by maximizing the evidence lower bound (ELBO):

$$ELBO = \log P_\theta(X) - \mathcal{D}[Q_\phi(z|X)||P_\theta(z|X)] \qquad (4)$$

The right hand side of equation (3) is something we can optimize by a gradient descent algorithm. $P_\theta(X|z)$ is modeled by the decoder network of the VAE parametrized by θ, and $Q_\phi(z|X)$ is modeled by the encoder network parametrized by ϕ. For continuous value inputs, $P_\theta(X|z)$ and $Q_\phi(z|X)$ are most commonly assumed to be Gaussian distributions [34]. $P(z)$ is a fixed prior distribution and assumed to be a standard multivariate normal distribution $\mathcal{N}(0, I)$. The first term $E_{z\sim Q_\phi}[logP_\theta(X|z)]$ is the expectation of the log probability of X given the encoder's output. Maximizing this term is equivalent to minimizing the reconstruction error of the autoencoder. The second term $D[Q_\phi(z|X)||P(z)]$ is the divergence between the approximated posterior distribution $Q_\phi(z|X)$ and the prior $P(z)$, and minimizing this term can be considered as adding a regularization term to prevent overfitting.

VAE is trained with the training data which follows a standard Gaussian distribution after z-score transformation. We impute missing values in the testing data with a trained VAE by an iterative process. Initially, the missing values are replaced with random values sampled from a standard Gaussian distribution. Then the following sequence of steps are repeated until an empirically determined iteration threshold is reached: compute the latent variable z distribution given input X with the encoder; take the mean of latent variable distribution as the input to the decoder, and compute the distribution of reconstructed data $\hat{X}$; take the mean of the reconstructed data distribution as the reconstructed values; replace the missing values with reconstructed values and leave non-missing values unchanged. The testing data should be scaled by the model's training data mean and variance before the imputation iterations, and inverse scaled after imputation.

Variational autoencoder imputation with shift correction

Regular implementation of VAE has an underlying assumption that the training data follows the same distribution of testing data. Below, we will discuss how to modify this assumption to better impute missing-not-at-random scenarios.

Since the VAE learns the data distribution from the training data, the output of imputation also follows the learnt distribution, which is similar to the training data. When the missing values are drawn from a different distribution than the training data, the imputation performance will drop due to the distribution shift. In the missing-not-at-random simulations where half of the lowest 10% values are masked, the missing values are considered to be shifted from the original training data to a smaller mean.

The lowest value missing scenario is a common type of missing values in biomedical data. When certain experimental conditions (e.g., low RNA sequencing depth) allow us to make assumptions that the majority of missing values are low expression values, we essentially have a prior knowledge that the distribution of missing values is shifted to the end of lower values. We can therefore use VAE with the shift-correction implementation. Recall that in Equation (3), the underlying assumption is that the training data follows a Gaussian distribution X ~ $\mathcal{N}(\mu, \sigma)$, where μ and σ are the outputs of the decoder network which represent the mean and variance of the observed training data, as well as the missing data. When the lowest values are missing, the learnt distribution has larger mean than the actual missing data, causing the reconstructed $\hat{X}$ to have larger values. To correct this, we modify the assumption of training data distribution to follow $\mathcal{N}(\mu + \lambda\sigma, \sigma)$, where μ and σ are the outputs of the decoder network which represent the mean and variance of the missing data, and λ is a hyperparameter. The mean of the observed training data is then shifted to $\mu + \lambda\sigma$. VAE with shift-correction is recommended to be used when certain experimental conditions warrant the assumption that missing values are

concentrated on the lower end of the data distribution. However, when such assumptions are unknown, or if the data missing pattern is more likely to be random, it is recommended to use the standard VAE without shift correction.

To test the lowest 10% missing case, we simulate a 10% lowest value missing scenario on the validation dataset, and select the shift correction parameter value that produces the smallest validation error. In reality, we may not know the actual range and amount of low value missing in the testing data and thus cannot simulate the situation on the validation data precisely. For a range of the lowest value missing scenarios, where half of the lowest 5%, 10%, 20%, and 30% values are missing respectively, we impute with a single λ which is selected based on the lowest 10% missing case. We thereby determine if it is possible to select λ without precise knowledge of the missing scenario on the testing data.

β-VAE

β-VAE is a generalization of the variational autoencoder with a focus to discover interpretable factorized latent factors [41]. A hyperparameter beta is introduced to the VAE loss to balance the reconstruction loss term with the regularization loss term. The loss of β-VAE is defined as:

$$L_{\beta-\mathrm{VAE}} = -E_{z\sim Q_{\phi}}[logP_{\theta}(X|z)] + \beta\mathcal{D}[Q_{\phi}(z|X)||P(z)] \quad (5)$$

where β is a hyperparameter.

β-VAE (β >1) has been shown to perform better than VAE in certain image generation tasks and has attracted increasing research interest [53]. However, no prior work has investigated the effect of β on imputation. Since VAE can be considered as a special case of β-VAE, we

extend our study to β-VAE with a varying β to further understand the effect of regularization on VAE imputation, and investigate potential possibility to increase its performance.

When β is 1, it is the same as VAE. When β>1, a stronger regularization is enforced, and the resulting latent space is smoother and more disentangled, which is a preferred property in certain learning tasks because more disentangled latent space has greater encoding efficiency [41].

On the other hand, when β=0, the regularization term is effectively removed. With the regularization term removed, the loss function only consists of the reconstruction loss term:

$$L_{\mathrm{VAE}'} = -E_{z\sim Q_{\phi}}[ogP_{\theta}(X|z)] \qquad (6)$$

which resembles the reconstruction loss function of a simple autoencoder (AE) without any regularization, that can usually be expressed in the mean squared error between the input X and the reconstruction X' [54] :

$$L(X, X') = \|X - X'\|_2^2 \qquad (7)$$

However, the loss of VAE without the regularization term as shown in equation (6) has a key difference from the loss of a simple autoencoder shown in equation (7). If (6) is viewed from a deterministic perspective, it is easy to distinguish the difference.

With the assumption that P_{θ} and $Q_{\phi}$are Gaussian distributions,

$$P_{\theta}\ (X|z) \sim N\ \left(X\middle|\mu_{\theta}(z),\ diag\left(\sigma_{\theta}(z)\right)\right),$$

$$Q_{\phi}\ (z|X) \sim N\ (z|\mu_{\phi}(X),\ diag\left(\sigma_{\phi}(X)\right))$$

the loss in (6) can be computed as the mean squared error between inputs and their mean reconstructions output by the decoder [34]:

$$L_{\mathrm{VAE}'} = \|X - \mu_\theta(z)\|_2^2 \qquad (8)$$

Unlike the deterministic reconstruction X' in equation (7), z in equation (8) is stochastic. However, the stochasticity of z can be relegated to a random variable that does not depend on ϕ, so that we can view (8) from a deterministic perspective. Using the reparameterization trick [33], z can be represented by:

$$z = \mu_\phi(X) + \sigma_\phi(X) \odot \varepsilon, \qquad \varepsilon \sim \mathcal{N}(0, I) \qquad (9)$$

where $\odot$ is the element-wise product. Therefore the input to the decoder can be considered as the output of encoder $\mu_\phi(X)$ corrupted by a random gaussian noise ε multiplied by $\sigma_\phi(X)$. Consequently, the loss in (8) can be considered as the loss of a deterministic autoencoder, which but has noise injected to the latent space. In contrast, noise is not present in the deterministic regular AE loss in (7).

We perform three random missing experiments (5%, 10%, 30% missing) with β-VAE and vary the hyperparameter β from 0, 1, 4, to 10, to evaluate how β affects imputation accuracies. This will help us understand the VAE mechanism and how to use it in imputation.

Model parameters and hyper-parameters tuning

Model parameters and hyper-parameters tuning are conducted on the validation dataset. The latent dimension is usually several magnitude smaller than the input dimension in autoencoder implementations, but there is no golden rule to determine its size. We test three latent dimension sizes: 50, 200, 400. Furthermore, we test two architectures with three or five

hidden layers. The hidden layers adjacent to the bottleneck layer has a 10-fold size increase, and each adjacent layer outwards after that has a constant size increase factor. For example, for a five hidden layer VAE with latent size 50, the hidden layer dimensions are 3000, 500, 50, 500, 3000, with input and output dimensions of 17176; for a three hidden layer VAE with latent size 200, the hidden layer dimensions are 2000, 200, 2000. We found that five hidden layers show better performance than three hidden layers, and that latent dimensions of 200 and 400 produce similar performances, both better than 50. We therefore use a VAE with five hidden layers of dimensions of 6000, 2000, 200, 2000, 6000 in our subsequent experiments. Figure S1 shows the performances differences of the six different model architectures described above. ReLU function is used as the activation function on the hidden layers.

We use the ADAM optimizer and search for optimal learning rates on a grid of 1e-5, 5e-5, 1e-4, 5e-4. A learning rate of 5e-5 is selected after grid search. We find that model performance is not very sensitive to batch size, and use a batch size of 250 and training epochs of 250. The number of iterations to perform the iterative imputation is also determined empirically. The imputed values are found to converge very quickly, and results remain mostly stable after 2 or 3 iterations. We use 3 as the iteration threshold.

Evaluation methods

To evaluate the VAE imputation framework, we compare it to other most commonly used missing value estimation methods: a K-nearest neighbor (KNN) method, and an iterative singular value decomposition (SVD) based method. We also construct a baseline using the mean value imputation method. KNN selects K number of samples which are most similar to the target sample with a missing gene based on Euclidean distance, and which all have values present in

that gene. Imputation is a weighted average of the values of that gene in those K samples. We chose K=10 in our evaluations based on a study which reported that K in the range of 10-25 gave the best imputation results [11]. Next, the SVD method decomposes the data matrix to a linear combination of eigengenes and corresponding coefficients. Genes are regressed against L most significant eigengenes, during which process the missing genes are not used [55]. The obtained coefficients are linearly multiplied by eigengenes to get a reconstruction with missing genes filled. This process is repeated until the total change in the matrix reaches a certain threshold. The reconstruction performance of SVD depends on the number of eigengenes selected for regression. We test a range of values and determine that the optimal performance is reached by full rank reconstruction. Hence we use full rank SVD in our evaluations. The mean value imputation method fills in the missing elements of each feature with the mean value of that feature across all non-missing samples.

We evaluate the root mean square error (RMSE) of the imputed data and uncorrupted ground truth,

$$RMSE = \frac{\sum_{i=1}^{n_{missing}} \sqrt{(x_i - x_i')^2}}{n_{missing}}$$

Where x_i is the ground truth of the masked value, and x_i' is the reconstructed value for the masked value.

To further evaluate the imputation effect on biomedical analysis, we compare the univariate correlation to clinical variables on the RNA sequencing data imputed by different methods. We conduct this analysis with the TCGA glioma cohort containing both LGG and GBM samples, and use two clinical variables: tumor histologic grade and survival time. The

tumor grade and survival information for each brain tumor patients are publicly available [56]. The histologic grade variable in the TCGA brain tumor data contains three levels: Grade II, III and IV, indicating increasing level of tumor malignancy. We directly use the grade value as an ordinal variable of three levels, and calculate the Spearman correlation coefficient between each gene and the grade variable. The survival time is a continuous variable measured in months, and the vital status indicates if the patient was dead or alive when the study concluded. With this information, we perform a cox regression on each gene with respect to the survival outcome, and compute the univariate coefficient of each gene. A concordance index is computed between the coefficient obtained from the imputed data by each method and the coefficients obtained from the ground truth. A higher concordance index indicates better resemblance to the true data.

Results

RMSE of imputation on RNA sequencing data

We inspect the RMSEs in different simulated missing scenarios by different imputation methods. The significant scores are calculated using the Wilcoxon test with the “ggsignif” package in R. First, we evaluate the missing-completely-at-random cases at varying percentage 5%, 10%, and 30% random elements in the testing data were masked respectively, and models were compared on the reconstruction RMSE. VAE achieves better RMSEs than KNN in all tested missing scenarios, and reaches similar or better performances than SVD in most scenarios (Figure 1a).

In the first missing-not-at-random simulation case, the masked values are confined to certain genes which have the highest 10% GC content. Genes whose GC content are in the top 10% contain 50% random missing values in the testing data. VAE shows better reconstruction

RMSE than KNN, and also achieves a slight advantage over SVD (Figure 1b). In the second case, 5% genes are masked entirely in the testing data. VAE again shows the lowest mean RMSE among competing methods (Figure 1c). Each method may have different performances on different genes. Supplementary Table 1 provides insight on the imputation results for individual genes, showing the RMSEs obtained from each method for each individual gene from one experimental trial.

The final missing-not-at-random case is based on the gene expression values. The extreme values at the lowest 10% quantile are masked 50% randomly in the testing data. As a result, the observed values in the testing data shifts its distribution from the training data, and results in a decreased performance of imputation. However, with shift-correction implementation, VAE again achieves similar or better imputation accuracy than other methods (Figure 1d).

The shift correction is robust to a range of low percentage missing scenarios

We further investigate the robustness of the shift correction parameter against a range of missing percentage on the lowest values. The shift correction parameter is selected based on a 10% lowest value missing scenario simulated on the validation data. We use the same selected parameter to test on a range of missing scenarios, where half of the lowest 5%, 10%, 20%, and 30% values are missing respectively. All methods show worse prediction errors for smaller thresholds of missing values, because smaller thresholds indicate that the missing values are concentrated to smaller values, leading to a larger shifts in data distribution. We show that in these tested scenarios the shift correction VAE consistently achieves better result than KNN and

SVD with the same λ (Figure 2). Therefore, λ selection does not need to exactly match the actual missing percentage, which is an advantage in real world implementations.

RMSE of imputation on DNA methylation data

For the imputation on DNA methylation data, the comparative performance of the KNN, SVD and VAE methods shows similar performance compared to the gene expression data. These three methods also show better performance than imputing with column mean. For missing-completely-at-random and block missing cases, VAE has similar performance as SVD, followed by KNN (Figure 3a, 3b). For the low coverage missing case, VAE achieves better RMSE than SVD and KNN (Figure 3c).

Correlation with clinical phenotypes

We investigate how closely the imputed data resembles the true data in terms of univariate correlation with respect to clinical variables. A higher concordance index between the correlation coefficients obtained from the imputed data and the coefficients obtained from the ground truth likely indicates the imputation method is better at preserving original data's univariate properties.

The ground truth of univariate Spearman correlations to histologic grade ranges from -1 to 1, with 46% of the genes having an absolute correlation value of 0.3 or greater. The majority of ground truth cox regression coefficients with respect to survival outcome is in the range of -5 and 5, with 72% of the genes having an absolute coefficient value of 0.3 or greater.

Table 2 contains the concordance indices from three imputation methods as well as a random imputation baseline. Random imputation is performed by filling the missing values by

random sampling the training data distribution. It shows that VAE and SVD are similar, and VAE and SVD achieve better concordance indices than KNN for both grade and survival outcome correlations. This suggests that VAE and SVD imputed data likely has a better resemblance to true data in the context of biomedical analysis for molecular biologists interested in specific genes in the presence of missing values. Figure 4 illustrates pairwise difference between the coefficients obtained from the ground truth and the coefficients obtained from the imputed data by KNN and VAE respectively, and shows sharper peaks around zero for VAE in all cases for histology and in most cases for survival. The pairwise differences are mostly distributed around zero, and a smaller variance around the zero indicates the pairwise differences are smaller overall. In each missing scenario VAE has a smaller variance than KNN across ten trials (all p values <0.005 in two sample t-tests).

Table 2. Correlation with clinical phenotypes (95% CI)

**(a)**	**KNN**	**VAE**	**SVD**	**Random**
**10% Random missing**	0.980±0.001	**0.982±0.001**	**0.982±0.001**	0.950±0.001
**Highest GC content missing**	0.949±0.002	**0.958±0.001**	**0.958±0.001**	0.816±0.006
**Entire genes missing**	0.918±0.005	0.932±0.004	**0.939±0.005**	0.500±0.004
**Lowest value missing**	0.977±0.001	0.983±0.001	**0.986±0.000**	0.906±0.007

**(b)**	**KNN**	**VAE**	**SVD**	**Random**
**10% Random missing**	0.969±0.002	**0.974±0.001**	**0.972±0.002**	0.873±0.050
**Highest GC content missing**	0.917±0.006	**0.931±0.004**	**0.933±0.006**	0.717±0.016
**Entire genes missing**	0.851±0.004	0.881±0.005	**0.906±0.006**	0.508±0.010
**Lowest value missing**	0.963±0.002	0.971±0.002	**0.976±0.002**	0.842±0.013

(a) Spearman correlation coefficient with tumor histologic grade; (b) Cox regression coefficient with survival outcome

Imputation time for new samples

The computation time for SVD or KNN to impute a single sample scales linearly with the dimension of the entire data matrix, while on the other hand, a VAE model can be pre-trained and applied directly to any given new sample to impute missing values. Once a VAE model is trained, the time to impute a new sample is almost negligible. VAE thus has the benefit of reducing computational cost especially at evaluation time.

Benchmark experiments are done on a 20 core cluster with Intel Xeon 2.40GHz CPUs, where the three methods are used to impute 100 samples in a gene expression matrix that consists of 6600 samples and 17176 genes. It takes an average of 2800 seconds to train the VAE network. In terms of evaluation time, the KNN method takes on average 8400 seconds, while SVD takes 36900 seconds, and VAE takes only 60 seconds, showing that VAE is several orders of magnitude faster at evaluation time.

$\boldsymbol{\beta}$-VAE and deterministic autoencoder

We perform three random missing experiments with β-VAE and vary the hyperparameter β from 0, 1, 4, to 10. Figure 5 shows that imputation results are similar for β=0 and β=1, while increasing β to larger values worsens the prediction accuracies.

The fact that β >1 produces worse imputation errors leads us to the hypothesis that the total loss of VAE (right hand side of equation (3)), consisting of the reconstruction loss and regularization loss, may be considered a tradeoff between reconstruction quality and latent space coding efficiency. If a greater emphasis is put on latent space regularization, the reconstruction

quality suffers. We conclude that stronger regularization does not help VAE's imputation performance.

Furthermore, when β=0, the imputation performance is similar to vanilla VAE ($\beta = 1$). Therefore, for imputation, removing latent space regularization will not affect performance. From previous discussion in the β-VAE method section, the loss of β-VAE with β=0 looks similar to that of a simple AE, but the key difference is that noise is injected to the latent space for of β-VAE (β=0). We find that with a simple AE, the imputation iterations cannot converge and the resulting RMSE is very large (not shown because non-convergence). This suggests that the noise injection to the latent space enables the imputation ability of the VAE.

Conclusion

We have described a deep learning imputation framework for transcriptome and methylome data using a variational autoencoder (VAE). We implement a shift correction method to improve VAE imputation performance on a commonly encountered missing-not-at-random scenario. We demonstrate that the proposed framework is competitive with SVD, which is a time-inefficient method for real world scenarios. We also show that VAE outperforms KNN in multiple scenarios such as bulk transcriptome and methylome data. VAE thus can be an important tool to analyze the large amounts of publicly available data from 1000s of studies including RNA sequencing and microarray data that are publicly available in the Gene Expression omnibus [38].

We provide insights on the effect of latent space regularization on imputation performance. We show that increasing latent space regularization in the VAE implementation leads to larger

error, and thus should be avoided in the imputation tasks. In addition, the regularization of latent space can be removed without affecting VAE's performance in imputation.

We also found that noise addition to the latent space largely helps VAE's good imputation performance, compared to a regular deterministic AE. The method of noise injection during training is reminiscent of denoising autoencoders (DAE). However, the noise addition for VAE and DAE are different. First, the noise in VAE depends on the input, whereas the DAE noise is independent of the input. Second, although noise addition to intermediate layers has been proposed in stacked denoising autoencoders for the purpose of representation learning [30], in most data imputation applications noise has only been added to the input layer of DAE [27, 57]. In contrast, noise is added to the latent space layer in VAE. It is not in the scope of this paper to evaluate how different noise addition schemes impact imputation and compare their performances. However, this may be worth exploring in future work.

Finally, in the context of imputing large dataset with high dimensional features, VAE has the potential benefit of reducing computational cost at evaluation time compared to SVD and KNN. This is because an autoencoder model can be pre-trained and applied directly to new samples, while SVD and KNN require computing the entire matrix each time a new sample is given.

In future work, it may be interesting to investigate VAE's application on single-cell RNA sequencing data, which has different missing scenarios than bulk RNA sequencing data. In addition, it may also be of interest to fully understand the effect of β in β-VAE when β is in the range from 0 to 1. Based on the hypothesis that there is a tradeoff between reconstruction quality and desired latent space property regulated by β, it can be expected that removing the regularization term (β=0) may even improve the vanilla VAE's (β=1) imputation performance. It is worth noting that such phenomenon did not occur, which invites further study.

Availability of Data and Materials

All data used in this manuscript are publicly available.

Gene expression data is version 2 of the adjusted pan-cancer gene expression data obtained from Synapse (synapse ID syn4976369) [58]. Clinical data of TCGA LGG/GBM can be found in the supplementary Table S1 in [56]. DNA methylation data is the WGBS data for BLUEPRINT methylomes (2016 release) obtained from rnbeads.org [59].

An archival copy of the code and supporting data is available via the GigaScience repository, GigaDB [60].

Availability of supporting source code and requirements

Project name: Genomic data imputation with variational autoencoders

Project home page: https://github.com/gevaertlab/BetaVAEImputation.

Operating system(s): Platform independent

Programming language: Python

Other requirements: Python 3.6.6 or higher, Pytorch 0.4.1

License: BSD 3-Clause License

RRID: SCR_018730

BiotoolsID: betavaeimputation

Declarations

Funding

Research reported in this publication was supported by the Fund for Innovation in Cancer Informatics (www.the-ici-fund.org), by the National Institute of Biomedical Imaging and Bioengineering of the National Institutes of Health (NIBIB https://www.nibib.nih.gov/), R01 EB020527 and R56 EB020527, and the National Cancer Institute (NCI https://www.cancer.gov/), U01 CA217851 and U01 CA199241, all to OG. The content is solely the responsibility of the authors and does not necessarily represent the official views of the National Institutes of Health. The funders had no role in study design, data collection and analysis, decision to publish, or preparation of the manuscript.

Competing interests

The authors declare that they have no competing interests.

Figures

Figure 1. Imputation RMSE on the gene expression data for (a) missing-completely-at-random cases of 5%, 10% and 30%; (b) half of the highest 10% GC content genes missing case; (c) 5% genes entirely missing case; (d) half of the lowest 10% values missing case. The numbers above bars show the Wilcoxon test significant scores between VAE or VAE with shift correction and other methods.

Figure 2. RMSE with 95% confidence interval for simulations where half of the lowest 5%, 10%, 20%, and 30% values are missing respectively. VAE-shift-correction results are achieved using a single λ which is selected based on the lowest 10% missing case.

Figure 3. Imputation RMSE on the DNA methylation data for (a) missing-completely-at-random cases of 5%, 10% and 30%; (b) 5% genes entirely missing; (c) half of the coverage <6 CpG sites missing. The numbers above bars show the Wilcoxon test significant scores between VAE and other methods.

Figure 4. Pairwise difference between the coefficients obtained from the ground truth and the coefficients obtained from the imputed data by KNN and VAE: (a) Spearman correlation coefficients with histologic grade; (b) Regression coefficients with survival outcome.

Figure 5. Imputation RMSE error of β-VAE on 5%, 10% and 30% random missing of gene expression, with β = 0, 1, 4, and 10, denoting increasing strength of regularization.

Supplementary Information

Figure S1. Model performances for six architectures: 3 hidden layers with latent sizes of 50, 200, and 400, and 5 hidden layers with latent sizes of 50, 200 and 400 respectively. Experiments are conducted on a simulated 10% random missing case.

Table S1. RMSEs from each method for each individual gene from one experimental trial in the second MNAR scenario for RNA sequencing data.

References

1. Kulis, M. and M. Esteller, *DNA methylation and cancer.* Adv Genet, 2010. **70**: p. 27-56.
2. Tomczak, K., P. Czerwinska, and M. Wiznerowicz, *The Cancer Genome Atlas (TCGA): an immeasurable source of knowledge.* Contemp Oncol (Pozn), 2015. **19**(1A): p. A68-77.
3. Byron, S.A., et al., *Translating RNA sequencing into clinical diagnostics: opportunities and challenges.* Nat Rev Genet, 2016. **17**(5): p. 257-71.
4. Litovkin, K., et al., *DNA Methylation-Guided Prediction of Clinical Failure in High-Risk Prostate Cancer.* PLoS One, 2015. **10**(6): p. e0130651.
5. Gevaert, O., R. Tibshirani, and S.K. Plevritis, *Pancancer analysis of DNA methylation-driven genes using MethylMix.* Genome Biol, 2015. **16**: p. 17.
6. Zheng, H., et al., *Benchmark of long non-coding RNA quantification for RNA sequencing of cancer samples.* GigaScience, 2019. **8**(12): p. giz145.
7. Champion, M., et al., *Module Analysis Captures Pancancer Genetically and Epigenetically Deregulated Cancer Driver Genes for Smoking and Antiviral Response.* EBioMedicine, 2018. **27**: p. 156-166.
8. Libbrecht, M.W. and W.S. Noble, *Machine learning applications in genetics and genomics.* Nat Rev Genet, 2015. **16**(6): p. 321-32.
9. Baghfalaki, T., M. Ganjali, and D. Berridge, *Missing Value Imputation for RNA-Sequencing Data Using Statistical Models:*

A Comparative Study. Journal of Statistical Theory and Applications, 2016. **15**.

10. Moorthy, K., et al., *Missing-values imputation algorithms for microarray gene expression data*, in *Microarray Bioinformatics*. 2019, Springer. p. 255-266.
11. Troyanskaya, O., et al., *Missing value estimation methods for DNA microarrays.* Bioinformatics, 2001. **17**(6): p. 520-5.
12. Faisal, S. and G. Tutz, *Missing value imputation for gene expression data by tailored nearest neighbors.* Stat Appl Genet Mol Biol, 2017. **16**(2): p. 95-106.
13. Smaragdis, P., B. Raj, and M. Shashanka, *Missing Data Imputation for Time-Frequency Representations of Audio Signals.* Journal of Signal Processing Systems, 2011. **65**(3): p. 361-370.
14. Yu, T., H. Peng, and W. Sun, *Incorporating nonlinear relationships in microarray missing value imputation.* IEEE/ACM Transactions on Computational Biology and Bioinformatics, 2010. **8**(3): p. 723-731.
15. Min, S., B. Lee, and S. Yoon, *Deep learning in bioinformatics.* Brief Bioinform, 2017. **18**(5): p. 851-869.
16. Chen, Y., et al., *Gene expression inference with deep learning.* Bioinformatics, 2016. **32**(12): p. 1832-9.
17. Leung, M.K., et al., *Deep learning of the tissue-regulated splicing code.* Bioinformatics, 2014. **30**(12): p. i121-9.
18. Arisdakessian, C., et al., *DeepImpute: an accurate, fast, and scalable deep neural network method to impute single-cell RNA-seq data.* Genome biology, 2019. **20**(1): p. 1-14.
19. Chen, C.L., et al., *Deep Learning in Label-free Cell Classification.* Scientific Reports, 2016. **6**: p. 21471.

20. Wulsin, D.F., et al., *Modeling electroencephalography waveforms with semi-supervised deep belief nets: fast classification and anomaly measurement.* J Neural Eng, 2011. **8**(3): p. 036015.
21. Jaques, N., et al., *Multimodal autoencoder: A deep learning approach to filling in missing sensor data and enabling better mood prediction.* 2018.
22. Vincent, P., et al., *Extracting and composing robust features with denoising autoencoders*, in *Proceedings of the 25th international conference on Machine learning*. 2008, ACM: Helsinki, Finland. p. 1096-1103.
23. Beaulieu-Jones, B.K. and J.H. Moore, *Missing Data Imputation in the Electronic Health Record Using Deeply Learned Autoencoders.* Pac Symp Biocomput, 2017. **22**: p. 207-218.
24. McCoy, J.T., S. Kroon, and L. Auret, *Variational Autoencoders for Missing Data Imputation with Application to a Simulated Milling Circuit.* IFAC-PapersOnLine, 2018. **51**(21): p. 141-146.
25. Mattei, P.-A. and J. Frellsen. *MIWAE: Deep Generative Modelling and Imputation of Incomplete Data Sets*. in *International Conference on Machine Learning*. 2019.
26. Eraslan, G., et al., *Single-cell RNA-seq denoising using a deep count autoencoder.* Nature communications, 2019. **10**(1): p. 390.
27. Costa, A.F., et al. *Missing data imputation via denoising autoencoders: the untold story*. in *International Symposium on Intelligent Data Analysis*. 2018. Springer.
28. Garciarena, U. and R. Santana, *An extensive analysis of the interaction between missing data types, imputation methods, and supervised classifiers.* Expert Systems with Applications, 2017. **89**: p. 52-65.
29. Vincent, P., et al. *Extracting and composing robust features with denoising autoencoders*. in *Proceedings of the 25th international conference on Machine learning*. 2008. ACM.
30. Vincent, P., et al., *Stacked Denoising Autoencoders: Learning Useful Representations in a Deep Network with a Local Denoising Criterion.* J. Mach. Learn. Res., 2010. **11**: p. 3371-3408.
31. Yeh, R.A., et al. *Semantic image inpainting with deep generative models*. in *Proceedings of the IEEE Conference on Computer Vision and Pattern Recognition*. 2017.
32. Hu, Z., et al. *Toward controlled generation of text*. in *Proceedings of the 34th International Conference on Machine Learning-Volume 70*. 2017. JMLR. org.
33. Kingma, D.P. and M. Welling, *Auto-encoding variational bayes.* arXiv preprint arXiv:1312.6114, 2013.
34. Ghosh, P., et al., *From variational to deterministic autoencoders.* arXiv preprint arXiv:1903.12436, 2019.
35. Way, G.P. and C.S. Greene, *Extracting a biologically relevant latent space from cancer transcriptomes with variational autoencoders.* BioRxiv, 2017: p. 174474.
36. Grønbech, C.H., et al., *scVAE: Variational auto-encoders for single-cell gene expression data.* bioRxiv, 2018: p. 318295.
37. Lopez, R., et al., *Deep generative modeling for single-cell transcriptomics.* Nature methods, 2018. **15**(12): p. 1053.
38. Barrett, T., et al., *NCBI GEO: archive for functional genomics data sets—update.* Nucleic acids research, 2012. **41**(D1): p. D991-D995.
39. Wheeler, D.L., et al., *Database resources of the national center for biotechnology information.* Nucleic acids research, 2006. **35**(suppl_1): p. D5-D12.

40. Aghdam, R., et al., *The Ability of Different Imputation Methods to Preserve the Significant Genes and Pathways in Cancer.* Genomics, Proteomics & Bioinformatics, 2017. **15**(6): p. 396-404.
41. Higgins, I., et al., *beta-VAE: Learning Basic Visual Concepts with a Constrained Variational Framework.* ICLR, 2017. **2**(5): p. 6.
42. Malta, T.M., et al., *Machine learning identifies stemness features associated with oncogenic dedifferentiation.* Cell, 2018. **173**(2): p. 338-354. e15.
43. *Synapse.org, https://www.synapse.org/#!Synapse:syn4976369.2. Accessed 1 Sept, 2019.*
44. Stunnenberg, H.G., et al., *The International Human Epigenome Consortium: a blueprint for scientific collaboration and discovery.* Cell, 2016. **167**(5): p. 1145-1149.
45. Gevaert, O., R. Tibshirani, and S.K. Plevritis, *Pancancer analysis of DNA methylation-driven genes using MethylMix.* Genome biology, 2015. **16**(1): p. 17.
46. Campbell, J.D., et al., *Genomic, pathway network, and immunologic features distinguishing squamous carcinomas.* Cell reports, 2018. **23**(1): p. 194-212. e6.
47. *Rnbeads.org, https://www.rnbeads.org/methylomes.html. Accessed 15 Sept, 2019.*
48. Little, R.J. and D.B. Rubin, *Statistical analysis with missing data*. Vol. 793. 2019: John Wiley & Sons.
49. Chen, Y.C., et al., *Effects of GC bias in next-generation-sequencing data on de novo genome assembly.* PLoS One, 2013. **8**(4): p. e62856.
50. Conesa, A., et al., *A survey of best practices for RNA-seq data analysis.* Genome Biol, 2016. **17**: p. 13.
51. Ballard, D.H., *Modular learning in neural networks*, in *Proceedings of the sixth National conference on Artificial intelligence - Volume 1*. 1987, AAAI Press: Seattle, Washington. p. 279-284.
52. Sakurada, M. and T. Yairi, *Anomaly Detection Using Autoencoders with Nonlinear Dimensionality Reduction*, in *Proceedings of the MLSDA 2014 2nd Workshop on Machine Learning for Sensory Data Analysis*. 2014, ACM: Gold Coast, Australia QLD, Australia. p. 4-11.
53. Burgess, C.P., et al., *Understanding disentangling in $\beta $-VAE.* arXiv preprint arXiv:1804.03599, 2018.
54. Kramer, M.A., *Nonlinear principal component analysis using autoassociative neural networks.* AIChE journal, 1991. **37**(2): p. 233-243.
55. Hastie, T., et al., *Imputingmissing data for gene expression arrays.* 1999.
56. Ceccarelli, M., et al., *Molecular profiling reveals biologically discrete subsets and pathways of progression in diffuse glioma.* Cell, 2016. **164**(3): p. 550-563.
57. Gondara, L. and K. Wang, *Multiple imputation using deep denoising autoencoders.* arXiv preprint arXiv:1705.02737, 2017.
58. Synapse.org: TCGA PancanAtlas Data, ID syn4976369.2 https://www.synapse.org/#!Synapse:syn4976369.2 accessed: 1 July 2020
59. RnBeads:R package for comprehensive analysis of DNA methylation data https://www.rnbeads.org/methylomes.html accessed 1 July 2020
60. Qiu, Y.Q., Zheng, H., and Gevaert, O., Supporting data for "Genomic data imputation with variational autoencoders". *GigaScience* Database. 2020. http://dx.doi.org/10.5524/100769.

Figure Click here to access/download;Figure;Figure1.png

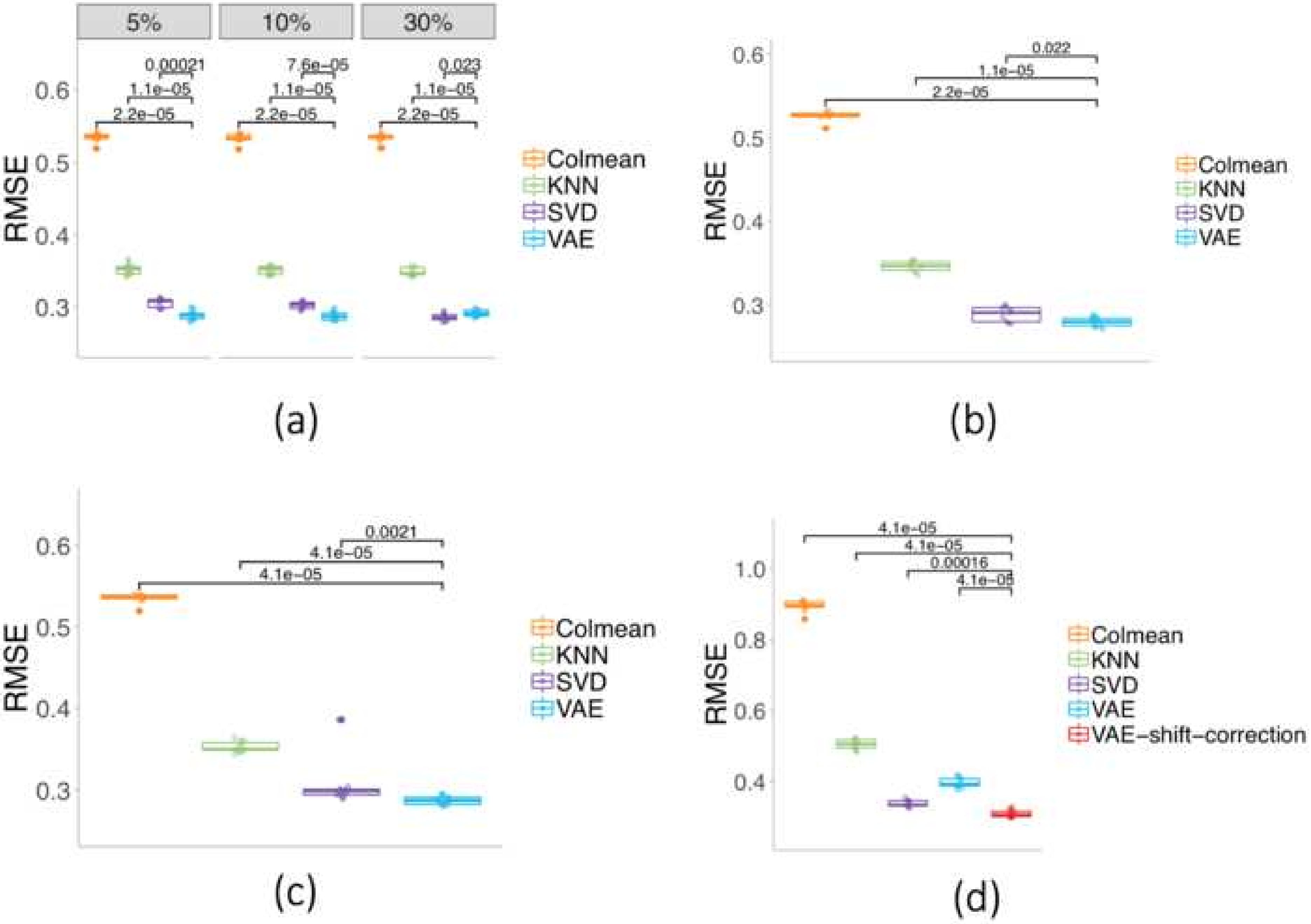

Figure

Click here to access/download;Figure;Figure2.png

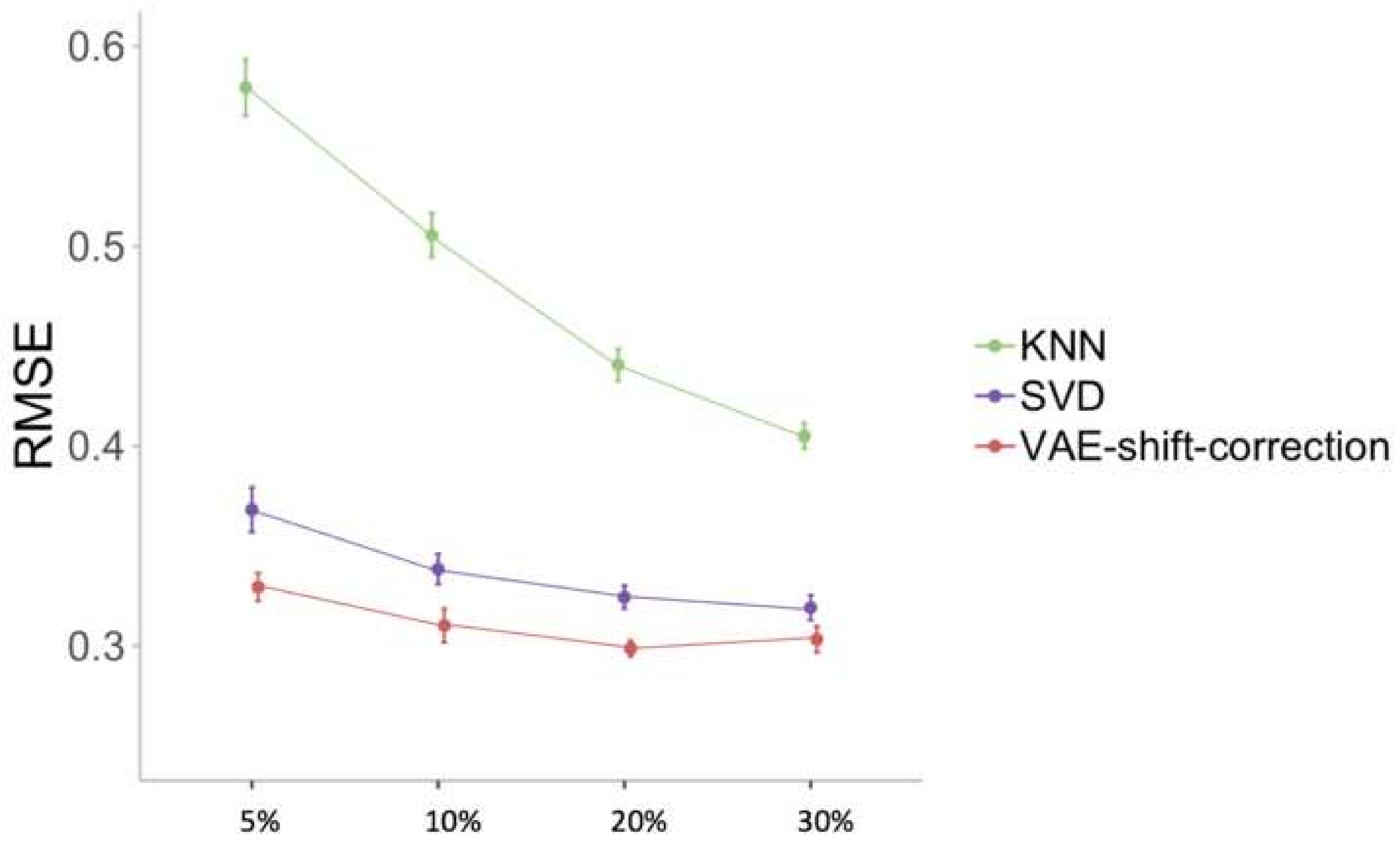

 Click here to access/download;Figure;Figure3.png

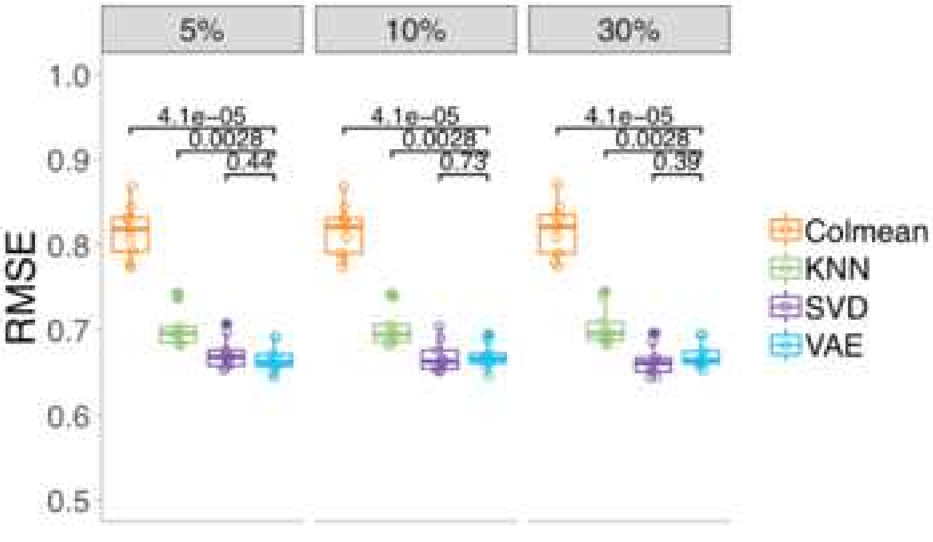

(a)

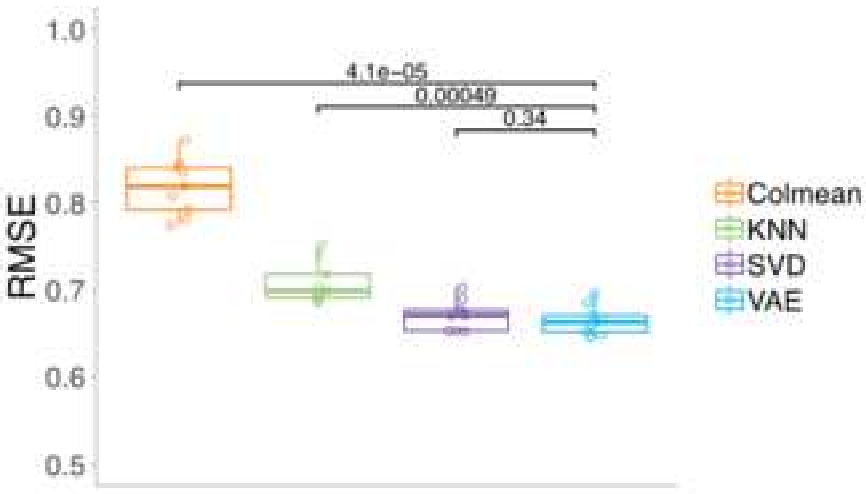

(b)

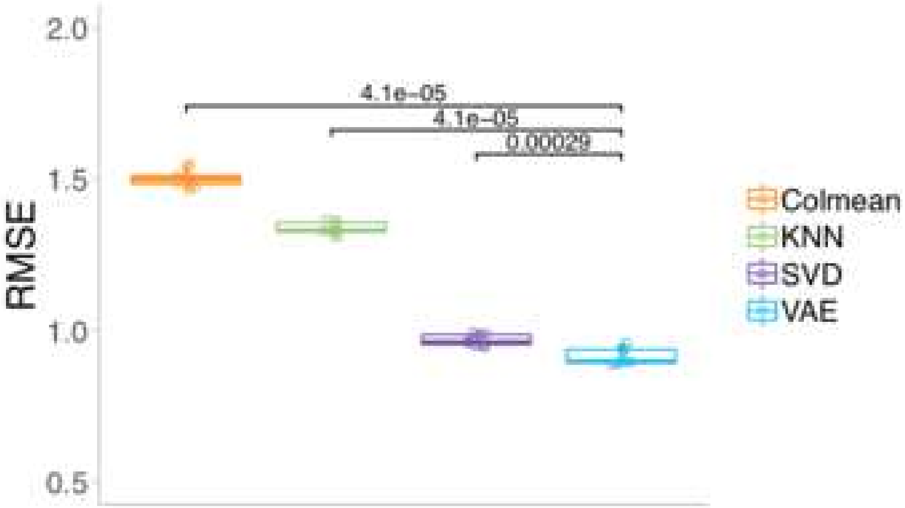

(c)

Figure

Click here to access/download;Figure;Figure4.png

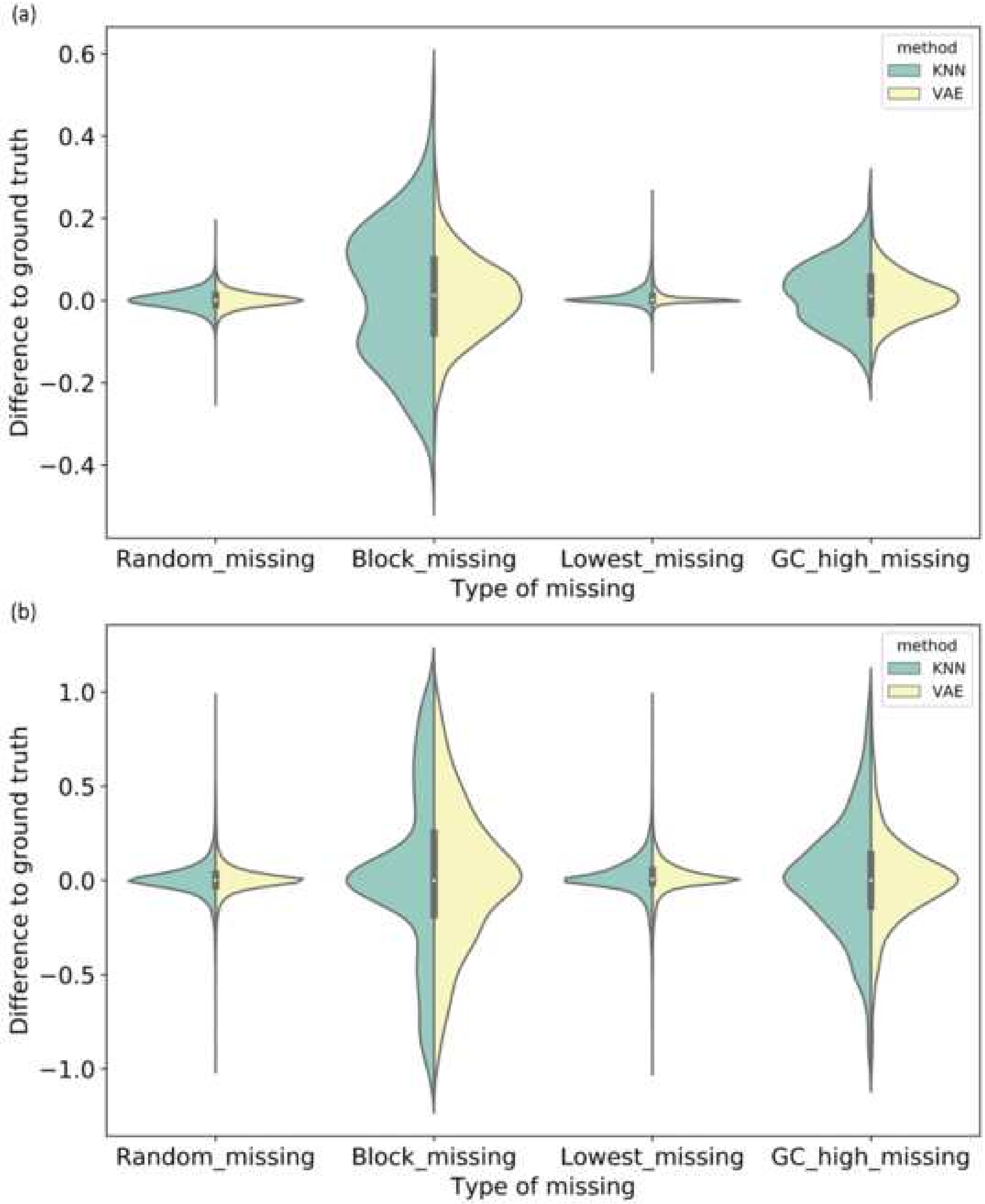

Figure

Click here to access/download;Figure;Figure5.png

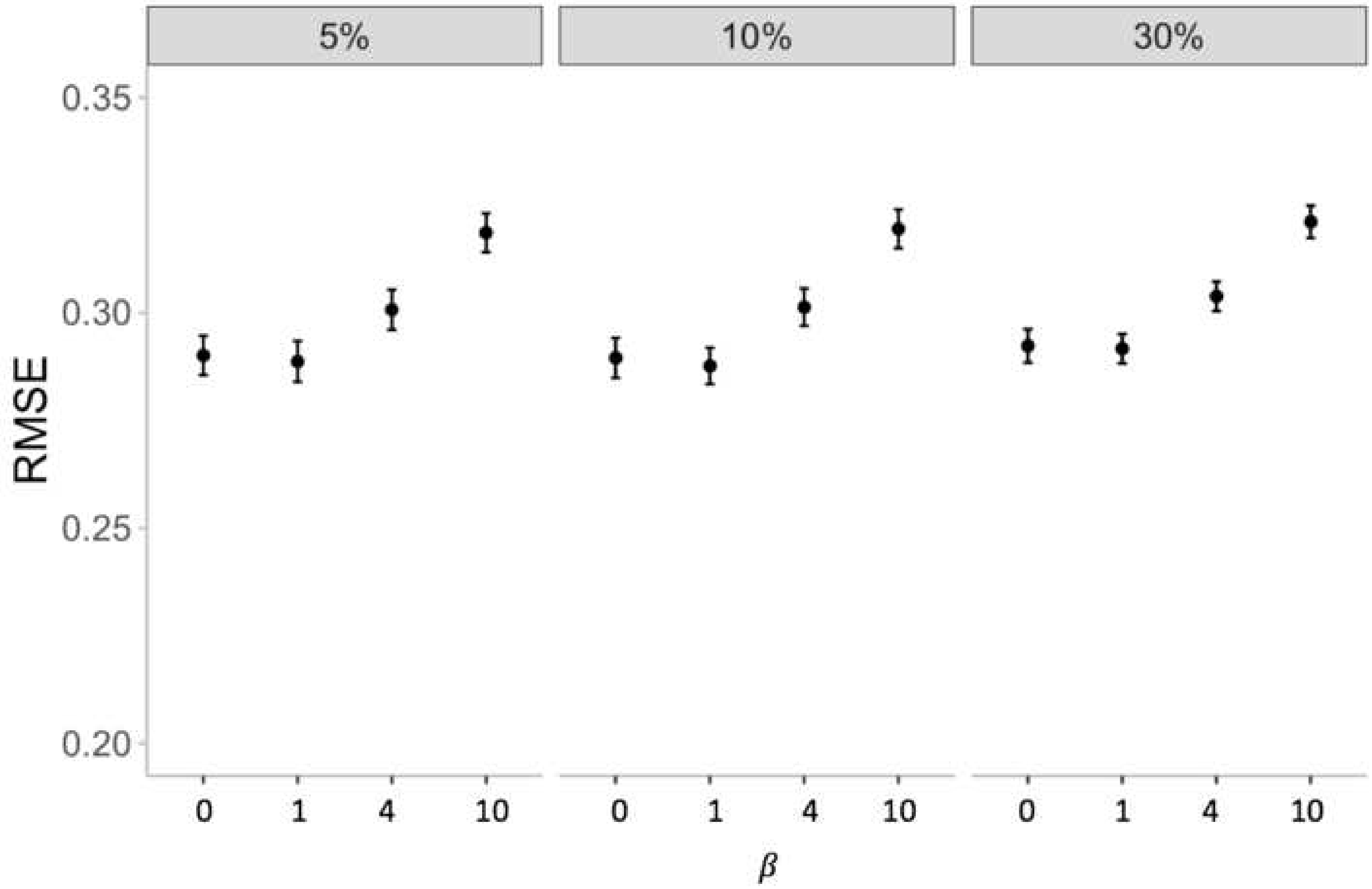

Click here to access/download
Supplementary Material
FigureS1.pdf

Click here to access/download
Supplementary Material
TableS1.csv
