# Supplementary figures and images for: Genomic data imputation with variational auto-encoders
Source: Gigascience. 2020 Aug 6;9(8):giaa082. doi: 10.1093/gigascience/giaa082 (PMC7407276; doi:10.1093/gigascience/giaa082)

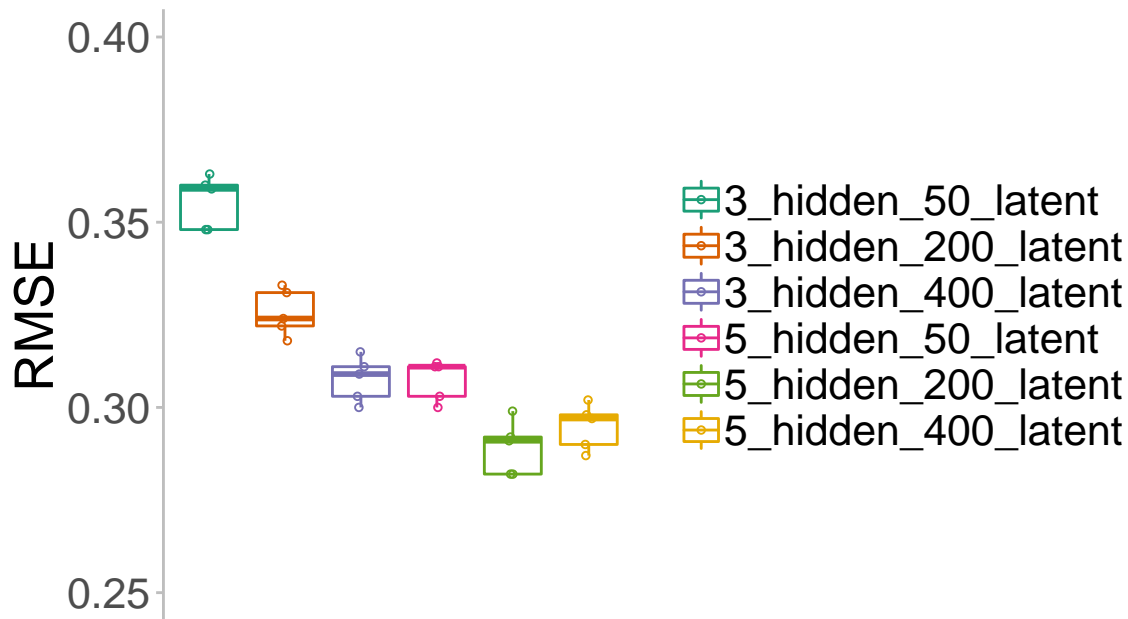

Supplement: giaa082_Supplemental_Figure_and_Table [file giaa082_supplemental_figure_and_table.zip › FigureS1.pdf]
