# Supplementary material for: Genomic data imputation with variational auto-encoders
Source: Gigascience. 2020 Aug 6;9(8):giaa082. doi: 10.1093/gigascience/giaa082 (PMC7407276; doi:10.1093/gigascience/giaa082)
Supplement: giaa082_Response_to_Reviewer_Comments_Original_Submission [file giaa082_response_to_reviewer_comments_original_submission.pdf]

Author's Response To Reviewer Comments

Close

Response to reviewers

We very much appreciate the reviewers' great efforts to scrutinize our study and raise important questions and suggestions. Their constructive comments are important and have helped us greatly to improve the quality of this manuscript. We have revised the manuscript following both reviewers' suggestions. In the following, we reply to the reviewers' comments.

Reviewer #1

Major Concerns:

1. What was the rationale for including a Beta-VAE in this study? The authors nicely explain "If a greater emphasis is put on latent space regularization, the reconstruction quality suffers". Why would a Beta-VAE be suitable for an imputation task then? Additional rationale about including the Beta-VAE model would be helpful.

We agree it is important to explain more clearly about the rationale for including Beta-VAE in our study. We made some modifications in the "Materials and Methods -> β-VAE" section, and reorganized the "Results -> β-VAE and deterministic autoencoder" section, so that it would hopefully be more clear why we thought it necessary to include the β-VAE analysis.

β-VAE (β >1) has been shown to perform better than VAE in certain image generation tasks and has attracted increasing research interest. However, no prior work has investigated the effect of β on imputation. Since VAE can be considered as a special case of β-VAE, we extend our study to β-VAE with a varying β to further understand the effect of regularization on VAE imputation, and to investigate potential possibility to increase its performance.

β-VAE did not turn out producing better imputation results. However, the study gave us insights in the aspects below (which are also explained in the results section).

1) The effect of regularization in VAE is not clearly known in advance. The result that β >1 produces worse imputation errors leads us to the hypothesis that the total loss of VAE may be considered as a tradeoff between reconstruction quality and latent space coding efficiency. If a greater emphasis is put on latent space regularization, the reconstruction quality suffers. We therefore conclude that stronger regularization does not help VAE's imputation performance.

2) Furthermore, when β=0, the imputation performance is similar to vanilla VAE (β=1). Therefore, for imputation, removing latent space regularization will not affect performance. From the discussion in the β-VAE method section, the loss of β-VAE with β=0 looks similar to that of a simple AE, but the key difference is that noise is injected to the latent space for of β-VAE (β=0). We find that with a simple AE, the imputation iterations cannot converge and the resulting RMSE is very large (not shown because non-convergence). This suggests that the noise injection to the latent space largely helps the imputation ability of the VAE.

Studying β-VAE helps us disentangle the different components in the loss function of VAE, and subsequently gain more insights on VAE's ability to impute values.

2. The simulations are well thought out and mostly described thoroughly (see minor concerns below). However, the simulations are missing important baselines and scenarios closer to real-world applications. For example, the VAE-shift-correction performance comparison is only shown in panel d in Figure 1. Is it true that in a real-world case, a user would default to using the VAE-shift-correction

model? If so, the authors should add the VAE-shift-correction performance to the other simulation tasks. Also, the "colmeans" performance shown in the DNA methylation graph is informative. The authors should add a similar baseline in the gene expression evaluation.

Per the reviewer's suggestion, we added a brief method description for Colmeans in the "Evaluation methods" section, and added "Colmeans" results in all simulation scenarios for RNA sequencing data (Figure 1).

We made modifications in the "Variational autoencoder imputation with shift correction" and "Missing data simulations" sections to clarify the different missing simulations.

The VAE-shift-correction only applies to the particular cases with prior knowledge. Without prior knowledge, VAE should be used as default. Panels A-D in Figure 1 each simulates a different missing case motivated by real world scenarios under different conditions. Panels A-C all belong to the VAE application, while Panel D belongs to the VAE-shit-correction application.

When we have some prior knowledge about the sequencing technology and parameters, we may be able to make some assumptions about the missing scenarios. When certain experimental conditions (e.g., low RNA sequencing depth) allow us to make assumptions that the majority of missing values are low expression values, and that the missing values are shifted from the seen value distribution, we can then choose to use VAE-shift-correction. A shift-correction model is robust in the sense that once a model is trained to accommodate shifting, its performance is not dramatically worsened if the actual testing data has larger or smaller shifting than the data it is trained on, and therefore we do not need an exact prior knowledge how much shifting has occurred in the data in order to train a model. If we do not know anything about the data types or experimental conditions that possibly cause shifts, it is advised to use the VAE without shift-correction.

3. The correlation analyses require more detail in order to adequately interpret. The authors report concordance between real and imputed values in two tasks: 1) Pearson correlation to tumor grade and 2) Cox regression coefficient with survival outcome. What are the ground truth estimates? Only the differences (fig 4) and concordance (table 1) to ground truth are shown. I imagine that ground truth correlations could be quite low. A low ground truth correlation would make it easy for random imputations to have high concordance and low difference. In addition to reporting the ground truth correlations, the authors should also add a random imputation baseline. Also, the purpose of figure 4 is to highlight the "sharper peaks around zero". This is not immediately clear for all comparisons. Is there a statistical test to confirm this observation? Lastly, in Table 1, since there are 10 different iterations, shouldn't the values have ranges?

We addressed all the issues accordingly in "Correlation with clinical phenotypes":
Ground truth correlations are described. They are not overwhelmingly low and the concordance indices comparisons are considered meaningful.
Random imputations results are added as a baseline in Table 2 (originally Table 1).
To confirm there are sharper peaks around zero for VAE than for KNN, in Figure 4, we compare the variances of the distributions across ten trials. A smaller variance indicates sharper peak. Student's t-test shows smaller variances for VAE than KNN in all cases with $p<0.005$.
In Table 2, we added 95% CI range for the values.

4. Model training details are absent from the methods. What is the VAE architecture (how many layers? How many latent dimensions?) What are the hyperparameters (learning rate, batch size, epochs, etc.)? Was cross validation performed? How did the authors select the size of the latent dimensions? Was there any attempt to improve model performance? These details are absolutely critical. It would also be informative to view imputation performance across rounds of decoding iterations.

We added the model training details in the "Model parameters and hyper-parameters tuning" section. We described how hyper-parameters (optimizer, learning rate, batch size, epochs, imputing iterations), and model parameters (layer, dimension) are chosen on the validation data. We also described how validation data is created in the "Missing data simulations".

5. More description of the clinical data is required. Where was this data retrieved from? Were there any additional data processing required?

We added the source of clinical data in “Availability of Data and Materials”, and also included more details about the format and processing of clinical data in “Evaluation methods”.

Minor Concerns:

6. In general, there may be too much detail describing the VAE and Beta-VAE. It is actually somewhat distracting. Would a citation and brief description suffice? The innovation in the manuscript is the imputation application, not the models.

We hope to provide not only a useful application, but also some insights on why the model works well, which may possibly facilitate future effort to improve the model further. Since our hypothesis and conclusions are mostly based on the fundamentals of VAE/beta-VAE, we hope the readers do not have to search extensively for their details elsewhere in order to understand them. The details of VAE and Beta-VAE may be helpful for readers to appreciate our conclusions and results better, and we therefore respectfully think that they should be included.

7. The term "covariate shift" is not specific. Please use a different term or define more specifically.

We replaced the term with a more specific description: “a missing-not-at-random scenario where the missing data distribution is not the same as the seen data”.

8. What is deterministic about an autoencoder? This part confused me. What implementation is being used? Autoencoders are typically initialized randomly and then trained using gradient descent.

Autoencoders are indeed initialized randomly and trained with gradient descent. By “deterministic”, we meant that there is no probabilistic modeling of the latent space, which is fundamentally different from a variational autoencoder. To clarity this point, we added a more detailed description in the “Variational autoencoder” section: “While in a regular autoencoder the latent space is encoded and then decoded deterministically, i.e., there is no probabilistic modelling of the latent space, a variational autoencoder (VAE) learns a probability distribution in the latent space.”

9. I think the simulation tasks are sufficient, but the authors decided to remove genes with missing values in a preprocessing step. How many genes were removed? What is the state of overall missingness? Is there a way to assess the added benefit of applying the VAE imputation in a real-world scenario with real missing genes? This would make for a compelling argument that imputation should occur more regularly. Since the authors are using GBM and LGG exclusively, is it possible to design some sort of a subtype clustering experiment comparing existing subtype labels and measuring an adjusted Rand index with and without imputation? (comparing of course to randomized imputation) Perhaps this is beyond the scope, but would be quite compelling.

We added description of the missing status of the original gene expression data in the section “Datasets”. The raw RNA sequencing data has a feature dimension of 20531 genes but contains NA values in 15% of the genes. Within the 15% of the genes who have missing values, on average 8.5% of the values are missing. The NA values are introduced in the pre-processing pipeline produced by Synapse.

We agree that it would be very interesting to assess imputation on the raw data without ground truth and investigate the impact of imputation on real-world biological scenarios other than histological grades and survival outcome, however, we also think that it belongs to a wider scope that is not the focus of this paper.

10. In the random 5% of genes simulation, is this 5% of all 17,176 genes without sampling restriction? It is surprising to me that the RMSE in figure 1C is so low. Perhaps because what's being plotted is actually mean RMSE? If individual gene RMSE is plotted, what does the distribution look like? This result probably has better real-world implications since a portion of the target audience is interested in imputing individual genes and might care more about the range of imputation than the average imputation.

Yes, there is no sampling restriction on 5% of the genes.

The plot is mean RMSE. Since there are 858 individual genes for each trial, we thought it is difficult to plot individual distributions and to compare across methods using individual distributions. Therefore, we consider the mean RMSE plot as a more practical overall graphic representation, which also allows us to compare across methods more easily. However, individual distributions for certain genes can be available upon request for target audience.

11. In the DNA methylation missing data simulations methods, the authors state: "We set the coverage threshold to six in our experiments". What units are being considered? 6 CpG sites per gene? What constitutes the gene region? Only CpG on gene bodies?

We clarified the definition of coverage threshold in the “Missing data simulations” section.

The coverage refers to the number of reads that can be mapped to a specific CpG site. Since we are using bisulfite sequencing data for DNA methylation, in the analysis, for each CpG site, we count how many reads supporting methylated status, and how many reads supporting unmethylated status. Some CpG sites may have very few reads mapped to them, which undermines the confidence in the measurement of methylation level. Thus, we choose an arbitrary threshold of six reads for the methylation status of a CpG site to be confidently determined. Methylation levels of CpGs with less than six reads mapped to them are treated as missing values in the analysis.

12. The simulation scenario is great! It is particularly nice to see 10 random trials being used for each comparison. A table describing simulation experiments could be very helpful.

A table is a great way to elucidate simulation experiments. We added Table 1 in the “Missing data simulations”.

13. In the imputation procedure during VAE training, the authors state: "Initially, the missing values are replaced with random values.". I don't think this is true. There are bounds placed on the random sampling, correct? What are these bounds? In this same paragraph, what is the iteration threshold?

Agreed. We clarified the initialization procedure and specified that the missing values are replaced with random values sampled from a standard Gaussian distribution.

The iteration threshold is also further clarified in the “Model parameters and hyper-parameters tuning” section. The number of iterations to perform the iterative imputation is also determined empirically. The imputed values are found to converge very quickly, and results remain mostly stable after 2 or 3 iterations. We use 3 as the iteration threshold.

14. The authors state: "the testing data is scaled by the training data mean and variance before the imputation iterations, and inverse scaled after imputation". This is not totally clear to me. For the testing data to be a true test set, it should not be influenced by the training set.

The mean and variance of training data can be considered as scaling parameters that are learnt from the training data. They can be used to scale any testing data for imputation. In this way, we are not tempering the testing data with any specific distribution of the testing data itself. This is a preprocessing step with a knowledge built in the model itself. We respectfully maintain that this is a fair operation.

15. The authors state: "Since the nature of the shift is relatively simple and known in advance, we leverage this knowledge to correct the shifting". The authors should elaborate on this point. In a real world, missing not at random case, how is the nature of the shift known in advance?

This point has been addressed in our response to the number two comment in “Major concerns”. Some conditions may lead to possible certain missing-not-at-random cases, for example, the sequencing depth in the RNA sequencing procedure may give us a measure of the degree of low expression level missing. We explained more about this in “Variational autoencoder imputation with shift correction”.

16. In the results section, the authors state "In all tested random missing scenarios VAE achieves better RMSE than KNN, and reaches similar or better performances [sic] than SVD. This is true in all cases except for 30% correct (Figure 1a)?

That is correct. In 30% random missing case and high GC content missing case, VAE and SVD are similar in performance (p >0.02), and so we modified the statement to be clearer: "VAE achieves better RMSEs than KNN in all tested missing scenarios, and reaches similar or better performances than SVD in most scenarios".

17. The deeper investigation into the shift correction approach is innovative and interesting! It is nice to see that the correction parameter is not very sensitive, and would indeed provide benefit in real world scenarios. The authors should add the shift correction VAE to panels A-C in figure 1 to further demonstrate its robustness.

This point has been addressed in our response to the reviewer's second comments. The shift correction model is not intended for A-C scenarios.

18. There are a few instances of misspelled words and incorrect grammar throughout the manuscript. For example, the sentence "Random half of the genes whose GC content are in the top 10% miss their values in the testing data" is grammatically incorrect. Also, watch for spelling in "for missing-comeletely-at-random and block missing cases…". The authors should carefully reread and correct these errors.

We have proofread the manuscript carefully and corrected such errors.

19. Please provide which version of the EB++AdjustPANCAN data on synapse was used.

Version 2 is used, and this information is added in "Availability of Data and Materials".

20. Please provide exactly which data in the rnbeads.org site was used.

DNA methylation data is the WGBS data for BLUEPRINT methylomes (2016 release). This is added in "Availability of Data and Materials".

21. It is great that the authors have provided their source code (and an open source license!) in a github repository. If possible, additional information on how the analysis can be reproduced (including how the scripts should be executed) with would be helpful.

We added a README file in the repository that explains how the analysis can be carried out.

Reviewer #2:

Data Questions:

1. What are the NA values in TCGA data? Were the NAs genes that had a count of zero? Did the authors do any additional filtering, I'm a bit surprised the remaining number is 17K.

The raw RNA sequencing data has a feature dimension of 20531 genes but contains NA values in 15% of the genes. Within the 15% of the genes who have missing values, on average 8.5% of the values are missing. The NA values are introduced in the pre-processing pipeline produced by Synapse. We did not do any additional filtering. We added this information in the "Datasets" section. In our study, in order to have a ground truth to evaluate imputation accuracies, we removed the NA values and carried out analysis with complete data. The missing values in our study were artificially introduced.

2. The authors mention disease type is how the RNA-Seq data was separated, what granularity of disease type? Is this high-level Cancer Types or do the authors separate by any sub-types?

The RNA-Seq data is glioma samples consisting of LGG and GBM. It is stratified by glioma subtypes (i.e., LGG versus GBM). We clarified this point in the "Missing data simulations" section.

Clinical correlation Questions:

3. The authors run a correlation analysis between clinical phenotypes and the imputed values. Additional details would help clarify how to interpret the results. For example, was this analysis done within cancer types or across cancer types? How were different histological grades transformed into values? Also, you mention that the correlation was initially done using spearman, however later you mention Pearson in the figure legends. Which package did you use to run this? If I want to redo your analysis, I need more details. I wasn't able to find further results in your GitHub, either.
Also, it is a little unclear to me what your motivation is to look at the concordance index between the correlation coefficients obtained from the imputed data. Why not look at which of the imputation methods provides values that are most predictive of the clinical phenotypes? Is it also possible to get error bars on the values in table 1?

The reviewer's questions help us make things clearer in this section. We addressed all the issues accordingly in "Correlation with clinical phenotypes":

The analysis was done with the TCGA glioma cohort containing both LGG and GBM samples.
The tumor grade and survival information for each brain tumor patients are publicly available (added data source in the availability section). The histologic grade variable in the TCGA brain tumor data contains three levels: Grade II, III and IV, indicating increasing level of tumor malignancy. We directly use the grade value as an ordinal variable of three levels, and calculate the Spearman correlation coefficient between each gene and the grade variable. "Pearson" is a typo in the figure legends and we corrected the error. We used "spearmanr" package in python to do this analysis. The script to carry out this analysis is added in the GitHub repository.
The 95% CI range for the values are added in Table 2 (originally Table 1).
In our analysis, we would like to limit our evaluation to measuring how much the imputed data resembles the ground truth. Concordance indices are an alternative way to evaluate which methods produce the imputed data that may resemble the ground truth better clinically. Building good predictive models with the imputed data (either univariate or multivariate) is usually a next step in biomedical data analysis. However, that is beyond the scope of this manuscript.

Plotting Questions:

4. To be more convincing that the author's method performs better, all boxplots where you compare against other methods require significance scores. (I think you are using ggplot, so it would be easy to add these using the ggsignif package).

We appreciate the reviewer's suggestion of the "ggsignif" package. We added significance scores (comparing VAE and other methods) in all the plots.

Training / Model Questions:

5. In regards to the shirt parameter, it is not clear to me how lambda was selected, the authors state "hyperparameter is selected on a validation data which simulates the lowest 10% missing case". What is the validation data in this case and how exactly is lambda learned? Is the validation data a completely held-out set that isn't used later? Did you do this shifted VAE for methylation data?

We clarified the definition of validation data in "Missing data simulations", and explained lambda selection in more details in "Variational autoencoder imputation with shift correction".

Each dataset is split into 80%-20% for training and hold-out testing in the imputation framework. The training dataset is further split into 80%-20%, where 20% is the validation dataset for hyper-parameter tuning. After hyper-parameters are selected, the entire training set is used for training.

To test the lowest 10% missing case, we simulate a 10% lowest value missing scenario on the validation dataset, and select the shift correction parameter value that produces the smallest validation error.

We did not use shift-correction VAE for methylation data. Each of the MNAR simulation is motivated by a different real-world condition specific to either gene expression data or methylation data. For RNA sequencing data, when the RNA sequencing depth is relatively low, genes that have low expression levels may not be detected because the few reads generated from this gene may not be captured. Therefore, we consider a possible scenario where lowly expressed genes are prone to be missing. For DNA methylation data, such scenario is not applicable. Instead, we simulate a different MNAR scenario where we mask CpG sites that have fewer coverage than a certain threshold. The coverage refers to the number of reads that can be mapped to a specific CpG site. Since we are using bisulfite sequencing data for DNA methylation, in the analysis, for each CpG site, we count how many reads supporting methylated status, and how many reads supporting unmethylated status. Some CpG sites may have very few reads mapped to them, which undermines the confidence in the measurement of methylation level. In such cases, the missing values themselves are not the lowest values as in the RNA sequencing data’s case. Therefore, we did not apply the shift-correction to methylation data. We clarified the shift-correction model’s use case in the “Variational autoencoder imputation with shift correction” and “Missing data simulations” sections.

6. What is the size of the encoder? How many hidden layers? What is the activation function?

We added the model training details in the “Model parameters and hyper-parameters tuning” section. We use a AE with five hidden layers and bottle neck size of 200. The activation function is ReLU. Other details including learning rate, batch size etc. are also included in this section.

General comments:

7. Reading this paper in the context of current genomics research, it may be useful to compare against a model in the wide array of single-cell data imputation models. This is an application where I can see the author's method being applied.

We agree that single-cell data imputation is an important application in genomics research. Single-cell data, however, has different missing case from bulk data, and applying the model on single-cell data will be a change of focus for this study.

A lot of historical bulk data are available for analysis through databases like the Gene Expression Omnibus (GEO) and the Short Read Archive (SRA). We also think that in the future, bulk data will still be important through the deconvolution of bulk gene expression. We therefore would like to have this manuscript focus on bulk data applications. However, we think that single-cell RNA sequencing data applications can be an interesting work in the future, and we included it when discussing future work in the conclusion section.

8. Also, I feel the statement "We show that noise addition to the latent space is the essential mechanism that enables VAE's good imputation performance, compared to a regular deterministic AE", is a bit strong. I think the authors have an experiment that may suggest this, but they did not show it was an essential mechanism.

We agree with the reviewer’s comment, and modified the statement. The revised sentence is “We also found that noise addition to the latent space largely helps VAE’s good imputation performance, compared to a regular deterministic AE.”

9. There are also some spelling mistakes, "form" instead of from in "Variational autoencoder imputation with shift correction", and "missing-no-at-random" in "RMSE of imputation on RNA sequencing data"

We have proofread carefully and corrected such errors.

Close
