# Supplementary material for: Genomic data imputation with variational auto-encoders
Source: Gigascience. 2020 Aug 6;9(8):giaa082. doi: 10.1093/gigascience/giaa082 (PMC7407276; doi:10.1093/gigascience/giaa082)
Supplement: giaa082_Response_to_Reviewer_Comments_Revision_1 [file giaa082_response_to_reviewer_comments_revision_1.pdf]

Author's Response To Reviewer Comments

Close

We greatly appreciate the reviewers' follow-up comments and suggestions. In addressing these comments, we were able to bring more clarity to the manuscript, for which we are sincerely thankful. Below, we provide a point-by-point response to the reviewers' comments.

1) Performance with Beta = 0 is quite similar to Beta = 1 is a very interesting result and an important observation! Thank you for highlighting this result in the revision. (this is not a suggestion, just a comment)

Thank you.

2) The added description of the parameter selection is top notch. It is very interesting that the five versus three hidden layers did not perform much differently. If possible, I recommend that the authors include a supplementary figure describing these experiments and performance differences.

Per reviewer's suggestion, we added a supplementary figure describing the performance differences from different model architectures. The selection of number of layers and latent size is indeed an important part of hyper-parameter selection. We agree that a figure on the experiment results may be a good reference to readers who need to determine the optimal model architectures for their data.

3) I now understand the VAE-shift-correction purpose and application. I also appreciate the authors updated recommendation. However, the authors should also make a recommendation about when NOT to use VAE-shift-correction.

We added a recommendation about when not to use VAE-shift-correction in the section "Variational autoencoder imputation with shift correction".

4) I agree with the decision to keep the mean gene RMSE in figure 1C. I do not think that plotting 858 individual genes would be super difficult, but it is definitely not necessary. Instead, it would be great if the authors could provide these estimates as a supplementary file (or even include it in their github repository).

We added a supplementary table on the error estimates for individual genes, obtained from each method.

5) The authors should explicitly state that they are using bisulfite sequencing. There are other ways of measuring DNA methylation. I had not realized this in my original read and it is not stated anywhere. At very minimum, this point should be at least mentioned once. This point is my strongest recommendation.

We agree. This point has been added in the data description section.

6) In response to the following point made by the authors: "The mean and variance of training data can be considered as scaling parameters that are learnt from the training data. They can be used to scale any testing data for imputation. In this way, we are not tempering the testing data with any specific distribution of the testing data itself. This is a preprocessing step with a knowledge built in the model itself. We respectfully maintain that this is a fair operation." I agree that this is standard practice, but I recommend that this is made more explicit. Unless the mean and variance of the training data are to be shipped with future software packages to perform the imputation, then this impact (if any, it might be extremely minor) has the potential to inflate test set performance.

We made it explicit in the code of the testing pipeline that the testing data should be scaled by the

model’s training data mean and variance. In the manuscript we also rephrased to emphasize this is a standard procedure anytime a model is trained or tested.

7) I certainly appreciate the updated and improved documentation in the github repository, but I could not reproduce the results. The authors should include the data used to train, or at least notes on how to access the data, in order for the code to be sufficiently reproducible.

We added notes in the repository’s README file on how to access the raw data, and further added the scripts on processing the raw data and creating training data (in different missing scenarios) to make the code reproducible.

Reviewer #2: The authors have thoroughly responded to all of my concerns, there are a few minor details remaining.

1) Regarding point 1, "What are the NA values in TCGA data?" I am still not clear how this happened. Is it caused by different reference annotations being used or are these zero values that have been replaced with an NA? After alignment, if they used the same annotation, there should be no NA's, but only counts. It is useful to know exactly where this came from in the pre-processing pipeline.

According to the data processing steps for the TCGA RNASeq data outlined on the website https://www.synapse.org/#!Synapse:syn4976363, after batch correction genes with mostly zero reads or with residual batch effects were removed from the adjusted samples and replaced with NAs. We added this detail in the “Datasets” section.

2) Regarding point 7 "Reading this paper in the context of current genomics research, it may be useful to compare against a model in the wide array of single-cell data imputation models. This is an application where I can see the author's method being applied.":

I still don't fully understand the practical application of your method for RNA-Seq data. When is data missing for bulk RNA-seq data? For microarray I am able to understand it, however in my experience, missing values are not typically seen in bulk RNA-Seq data. In RNA-Seq, one would see counts that are lower than expected in a specific sample due to GC-content biases, or a count of zero when the true count is very low. In practical terms, when I run a bulk RNA-Seq experiment, how would I use your method? Would it be to 1) replace genes with 0 counts with an NA? 2) to replace genes with a lower than expected count with an NA? 3) to be used in panel based sequencing similar to the LINCS L1000? If 1 or 2, how would be be able to distinguish between an abnormal count and a "true" count? If 3, then you would need to show imputation for a larger amount of missingness
I think if this was further elaborated it would really strengthen the paper as well as give more credence to the percentages missing in your simulation.

The use of VAE is indeed not limited to RNA sequencing data, as we have shown with DNA methylation data as well. We agree with the reviewer’s comment that missing values may be more typically seen in the microarray data (especially random missing cases). One potential practical application for this method, as stated in the manuscript “Conclusion” section, is to analyze the large amounts of publicly available data in the Gene Expression omnibus, including a lot of microarray data.

For RNA sequencing data, missingness may usually result from data processing procedures. Related to the reviewer’s first point, in the practical case of Synapse processing for example, after batch correction genes with residual batch effects in the adjusted samples were replaced as NA. In such cases, we can use VAE to impute the missing values in the RNA sequencing data. This case may resemble the simulated MNAR case where some genes are entirely missing in some samples. We added some motivation of the second of MNAR simulation in the “Missing data simulations” section.

There may also be cases where NA are not inherently present, but certain values may be considered NA. For example, the reviewer mentioned the case where genes have lower than expected counts due to GC-content biases. In such case, researchers may either choose to proceed without any processing, or to consider genes with lower than expected counts as NA and use methods to fill them in with possibly more accurate values. We expect that good imputation methods will respect the true values, and produce close to zero values for the genes that actually have low or zero values.

3) The authors state "In each missing scenario VAE has a smaller variance than KNN across ten trials (all p values <0.005)." What test was performed?

Two sample t-tests were performed. This is added in the sentence.

4) Introduction, first sentence "researches" should be "researchers"

Thank you for catching the typo.

Close
