# Supplementary material for: Genomic data imputation with variational auto-encoders
Source: Gigascience. 2020 Aug 6;9(8):giaa082. doi: 10.1093/gigascience/giaa082 (PMC7407276; doi:10.1093/gigascience/giaa082)
Supplement: giaa082_Response_to_Reviewer_Comments_Revision_2 [file giaa082_response_to_reviewer_comments_revision_2.pdf]

Author's Response To Reviewer Comments

Close

We have addressed the reviewer's final comment and the editorial comments in this revision. We thank the editor and reviewers' great effort in helping to improve the quality of this manuscript.

Close
