# Supplementary material for: Genomic data imputation with variational auto-encoders
Source: Gigascience. 2020 Aug 6;9(8):giaa082. doi: 10.1093/gigascience/giaa082 (PMC7407276; doi:10.1093/gigascience/giaa082)
Supplement: giaa082_Reviewer_1_Report_Original_Submission — Gregory Way -- 4/9/2020 Reviewed [file giaa082_reviewer_1_report_original_submission.pdf]

Reviewer Report

Title: Genomic data imputation with variational autoencoders

Version: Original Submission **Date:** 4/9/2020

Reviewer name: Gregory Way

Reviewer Comments to Author:

Qiu et al. investigate the performance of imputing missing values in gene expression and DNA methylation data using a variational autoencoder (VAE). The authors compare their approach to two other well characterized approaches: K-Nearest Neighbors (KNN) and Singular Value Decomposition (SVD). The authors also test imputation performance with a Beta-VAE. The primary evaluation task is to compare average root mean squared error in a variety of simulation scenarios. The authors also compare imputations to ground truth in a correlation analysis. In nearly every case, the VAE outperforms the other methods. In general, the core message is clear and the results show a clear performance benefit of using VAE to impute missing values. However, the methods are not completely reported, several baselines are not adequately addressed, and additional rationale is required to increase confidence in the approach. Below, I describe a few major and several minor concerns:
Major Concerns:

* What was the rationale for including a Beta-VAE in this study? The authors nicely explain "If a greater emphasis is put on latent space regularization, the reconstruction quality suffers". Why would a Beta-VAE be suitable for an imputation task then? Additional rationale about including the Beta-VAE model would be helpful.
* The simulations are well thought out and mostly described thoroughly (see minor concerns below). However, the simulations are missing important baselines and scenarios closer to real-world applications. For example, the VAE-shift-correction performance comparison is only shown in panel d in Figure 1. Is it true that in a real-world case, a user would default to using the VAE-shift-correction model? If so, the authors should add the VAE-shift-correction performance to the other simulation tasks. Also, the "colmeans" performance shown in the DNA methylation graph is informative. The authors should add a similar baseline in the gene expression evaluation.
* The correlation analyses require more detail in order to adequately interpret. The authors report concordance between real and imputed values in two tasks: 1) Pearson correlation to tumor grade and 2) Cox regression coefficient with survival outcome. What are the ground truth estimates? Only the differences (fig 4) and concordance (table 1) to ground truth are shown. I imagine that ground truth correlations could be quite low. A low ground truth correlation would make it easy for random imputations to have high concordance and low difference. In addition to reporting the ground truth correlations, the authors should also add a random imputation baseline. Also, the purpose of figure 4 is to highlight the "sharper peaks around zero". This is not immediately clear for all comparisons. Is there a statistical test to confirm this observation? Lastly, in Table 1, since there are 10 different iterations, shouldn't the values have ranges?
* Model training details are absent from the methods. What is the VAE architecture (how many

layers? How many latent dimensions?) What are the hyperparameters (learning rate, batch size, epochs, etc.)? Was cross validation performed? How did the authors select the size of the latent dimensions? Was there any attempt to improve model performance? These details are absolutely critical. It would also be informative to view imputation performance across rounds of decoding iterations.

* More description of the clinical data is required. Where was this data retrieved from? Were there any additional data processing required?

Minor Concerns:

* In general, there may be too much detail describing the VAE and Beta-VAE. It is actually somewhat distracting. Would a citation and brief description suffice? The innovation in the manuscript is the imputation application, not the models.
* The term "covariate shift" is not specific. Please use a different term or define more specifically.
* What is deterministic about an autoencoder? This part confused me. What implementation is being used? Autoencoders are typically initialized randomly and then trained using gradient descent.
* I think the simulation tasks are sufficient, but the authors decided to remove genes with missing values in a preprocessing step. How many genes were removed? What is the state of overall missingness? Is there a way to assess the added benefit of applying the VAE imputation in a real-world scenario with real missing genes? This would make for a compelling argument that imputation should occur more regularly. Since the authors are using GBM and LGG exclusively, is it possible to design some sort of a subtype clustering experiment comparing existing subtype labels and measuring an adjusted Rand index with and without imputation? (comparing of course to randomized imputation) Perhaps this is beyond the scope, but would be quite compelling.
* In the random 5% of genes simulation, is this 5% of all 17,176 genes without sampling restriction? It is surprising to me that the RMSE in figure 1C is so low. Perhaps because what's being plotted is actually mean RMSE? If individual gene RMSE is plotted, what does the distribution look like? This result probably has better real-world implications since a portion of the target audience is interested in imputing individual genes and might care more about the range of imputation than the average imputation.
* In the DNA methylation missing data simulations methods, the authors state: "We set the coverage threshold to six in our experiments". What units are being considered? 6 CpG sites per gene? What constitutes the gene region? Only CpG on gene bodies?
* The simulation scenario is great! It is particularly nice to see 10 random trials being used for each comparison. A table describing simulation experiments could be very helpful.
* In the imputation procedure during VAE training, the authors state: "Initially, the missing values are replaced with random values.". I don't think this is true. There are bounds placed on the random sampling, correct? What are these bounds? In this same paragraph, what is the iteration threshold?
* The authors state: "the testing data is scaled by the training data mean and variance before the imputation iterations, and inverse scaled after imputation". This is not totally clear to me. For the testing data to be a true test set, it should not be influenced by the training set.
* The authors state: "Since the nature of the shift is relatively simple and known in advance, we leverage this knowledge to correct the shifting". The authors should elaborate on this point. In a real world, missing not at random case, how is the nature of the shift known in advance?
* In the results section, the authors state "In all tested random missing scenarios VAE achieves better

RMSE than KNN, and reaches similar or better performances [sic] than SVD. This is true in all cases except for 30% correct (Figure 1a)?

* The deeper investigation into the shift correction approach is innovative and interesting! It is nice to see that the correction parameter is not very sensitive, and would indeed provide benefit in real world scenarios. The authors should add the shift correction VAE to panels A-C in figure 1 to further demonstrate its robustness.

* There are a few instances of misspelled words and incorrect grammar throughout the manuscript. For example, the sentence "Random half of the genes whose GC content are in the top 10% miss their values in the testing data" is grammatically incorrect. Also, watch for spelling in "for missing-comeletely-at-random and block missing cases…". The authors should carefully reread and correct these errors.

* Please provide which version of the EB++AdjustPANCAN data on synapse was used.

* Please provide exactly which data in the rnbeads.org site was used.

* It is great that the authors have provided their source code (and an open source license!) in a github repository. If possible, additional information on how the analysis can be reproduced (including how the scripts should be executed) with would be helpful.

Level of Interest

Please indicate how interesting you found the manuscript: Choose an item.

Quality of Written English

Please indicate the quality of language in the manuscript: Choose an item.

Declaration of Competing Interests

Please complete a declaration of competing interests, considering the following questions:

- Have you in the past five years received reimbursements, fees, funding, or salary from an organisation that may in any way gain or lose financially from the publication of this manuscript, either now or in the future?
- Do you hold any stocks or shares in an organisation that may in any way gain or lose financially from the publication of this manuscript, either now or in the future?
- Do you hold or are you currently applying for any patents relating to the content of the manuscript?
- Have you received reimbursements, fees, funding, or salary from an organization that holds or has applied for patents relating to the content of the manuscript?
- Do you have any other financial competing interests?
- Do you have any non-financial competing interests in relation to this paper?

If you can answer no to all of the above, write 'I declare that I have no competing interests' below. If your reply is yes to any, please give details below.

I declare that I have no competing interests

I agree to the open peer review policy of the journal. I understand that my name will be included on my report to the authors and, if the manuscript is accepted for publication, my named report including any attachments I upload will be posted on the website along with the authors' responses. I agree for my report to be made available under an Open Access Creative Commons CC-BY license (http://creativecommons.org/licenses/by/4.0/). I understand that any comments which I do not wish to be included in my named report can be included as confidential comments to the editors, which will not be published.

Choose an item.

To further support our reviewers, we have joined with Publons, where you can gain additional credit to further highlight your hard work (see: https://publons.com/journal/530/gigascience). On publication of this paper, your review will be automatically added to Publons, you can then choose whether or not to claim your Publons credit. I understand this statement.

Yes Choose an item.
