# Supplementary material for: Genomic data imputation with variational auto-encoders
Source: Gigascience. 2020 Aug 6;9(8):giaa082. doi: 10.1093/gigascience/giaa082 (PMC7407276; doi:10.1093/gigascience/giaa082)
Supplement: giaa082_Reviewer_1_Report_Revision_1 — Gregory Way -- 5/31/2020 Reviewed [file giaa082_reviewer_1_report_revision_1.pdf]

Reviewer Report

Title: Genomic data imputation with variational autoencoders

Version: Revision 1 **Date:** 5/31/2020

Reviewer name: Gregory Way

Reviewer Comments to Author:

The authors have very nicely responded to all of my comments and have made appropriate modifications in the text. I think the paper, as presented in revision 1, represents a valuable contribution as is. Therefore, all subsequent comments should be considered suggestions, but I do strongly recommend that they are completed.

1) Performance with Beta = 0 is quite similar to Beta = 1 is a very interesting result and an important observation! Thank you for highlighting this result in the revision. (this is not a suggestion, just a comment)
2) The added description of the parameter selection is top notch. It is very interesting that the five versus three hidden layers did not perform much differently. If possible, I recommend that the authors include a supplementary figure describing these experiments and performance differences.
3) I now understand the VAE-shift-correction purpose and application. I also appreciate the authors updated recommendation. However, the authors should also make a recommendation about when NOT to use VAE-shift-correction.
4) I agree with the decision to keep the mean gene RMSE in figure 1C. I do not think that plotting 858 individual genes would be super difficult, but it is definitely not necessary. Instead, it would be great if the authors could provide these estimates as a supplementary file (or even include it in their github repository).
5) The authors should explicitly state that they are using bisulfite sequencing. There are other ways of measuring DNA methylation. I had not realized this in my original read and it is not stated anywhere. At very minimum, this point should be at least mentioned once. This point is my strongest recommendation.
6) In response to the following point made by the authors: "The mean and variance of training data can be considered as scaling parameters that are learnt from the training data. They can be used to scale any testing data for imputation. In this way, we are not tempering the testing data with any specific distribution of the testing data itself. This is a preprocessing step with a knowledge built in the model itself. We respectfully maintain that this is a fair operation." I agree that this is standard practice, but I recommend that this is made more explicit. Unless the mean and variance of the training data are to be shipped with future software packages to perform the imputation, then this impact (if any, it might be extremely minor) has the potential to inflate test set performance.
7) I certainly appreciate the updated and improved documentation in the github repository, but I could not reproduce the results. The authors should include the data used to train, or at least notes on how to access the data, in order for the code to be sufficiently reproducible.

Level of Interest

Please indicate how interesting you found the manuscript: Choose an item.

Quality of Written English

Please indicate the quality of language in the manuscript: Choose an item.

Declaration of Competing Interests

Please complete a declaration of competing interests, considering the following questions:

- Have you in the past five years received reimbursements, fees, funding, or salary from an organisation that may in any way gain or lose financially from the publication of this manuscript, either now or in the future?
- Do you hold any stocks or shares in an organisation that may in any way gain or lose financially from the publication of this manuscript, either now or in the future?
- Do you hold or are you currently applying for any patents relating to the content of the manuscript?
- Have you received reimbursements, fees, funding, or salary from an organization that holds or has applied for patents relating to the content of the manuscript?
- Do you have any other financial competing interests?
- Do you have any non-financial competing interests in relation to this paper?

If you can answer no to all of the above, write 'I declare that I have no competing interests' below. If your reply is yes to any, please give details below.

I declare that I have no competing interests

I agree to the open peer review policy of the journal. I understand that my name will be included on my report to the authors and, if the manuscript is accepted for publication, my named report including any attachments I upload will be posted on the website along with the authors' responses. I agree for my report to be made available under an Open Access Creative Commons CC-BY license (http://creativecommons.org/licenses/by/4.0/). I understand that any comments which I do not wish to be included in my named report can be included as confidential comments to the editors, which will not be published.

Choose an item.

To further support our reviewers, we have joined with Publons, where you can gain additional credit to further highlight your hard work (see: https://publons.com/journal/530/gigascience). On publication of

this paper, your review will be automatically added to Publons, you can then choose whether or not to claim your Publons credit. I understand this statement.

Yes Choose an item.
