# Supplementary material for: Genomic data imputation with variational auto-encoders
Source: Gigascience. 2020 Aug 6;9(8):giaa082. doi: 10.1093/gigascience/giaa082 (PMC7407276; doi:10.1093/gigascience/giaa082)
Supplement: giaa082_Reviewer_2_Report_Original_Submission — Natalie Davidson -- 4/21/2020 Reviewed [file giaa082_reviewer_2_report_original_submission.pdf]

Reviewer Report

Title: Genomic data imputation with variational autoencoders

Version: Original Submission **Date:** 4/21/2020

Reviewer name: Natalie Davidson

Reviewer Comments to Author:

The authors approached the missing-not-at-random problem for transcriptomic and methylation data. They compare against SVD and KNN, (also additionally B-VAE, plus VAE with a variance shift term) using the RMSE and correlation with clinical covariates as metrics. I think this is a nice idea and seems correct in implementations. However, I find some parts of the paper are not fully explained.
Data Questions:
What are the NA values in TCGA data? Were the NAs genes that had a count of zero? Did the authors do any additional filtering, I'm a bit surprised the remaining number is 17K.
The authors mention disease type is how the RNA-Seq data was separated, what granularity of disease type? Is this high-level Cancer Types or do the authors separate by any sub-types?
Clinical correlation Questions:
The authors run a correlation analysis between clinical phenotypes and the imputed values. Additional details would help clarify how to interpret the results. For example, was this analysis done within cancer types or across cancer types? How were different histological grades transformed into values? Also, you mention that the correlation was initially done using spearman, however later you mention Pearson in the figure legends. Which package did you use to run this? If I want to redo your analysis I need more details. I wasn't able to find further results in your github, either.
Also, it is a little unclear to me what your motivation is to look at the concordance index between the correlation coefficients obtained from the imputed data. Why not look at which of the imputation methods provides values that are most predictive of the clinical phenotypes? Is it also possible to get error bars on the values in table 1?
Plotting Questions:
To be more convincing that the author's method performs better, all boxplots where you compare against other methods require significance scores. (I think you are using ggplot, so it would be easy to add these using the ggsignif package).
Training / Model Questions:
In regards to the shirt parameter, it is not clear to me how lambda was selected, the authors state "hyperparameter ð□œ† is selected on a validation data which simulates the lowest 10% missing case". What is the validation data in this case and how exactly is lambda learned? Is the validation data a completely held-out set that isn't used later? Did you do this shifted VAE for methylation data?
What is the size of the encoder? How many hidden layers? What is the activation function?
General comments:
Reading this paper in the context of current genomics research, it may be useful to compare against a model in the wide array of single-cell data imputation models. This is an application where I can see the

author's method being applied.
Also, I feel like the statement "We show that noise addition to the latent space is the essential mechanism that enables VAE's good imputation performance, compared to a regular deterministic AE", is a bit strong. I think the authors have an experiment that may suggest this, but they did not show it was an essential mechanism.
There is also some spelling mistakes, "form" instead of from in "Variational autoencoder imputation with shift correction", and "missing-no-at-random" in "RMSE of imputation on RNA sequencing data"

Level of Interest

Please indicate how interesting you found the manuscript: Choose an item.

Quality of Written English

Please indicate the quality of language in the manuscript: Choose an item.

Declaration of Competing Interests

Please complete a declaration of competing interests, considering the following questions:

- Have you in the past five years received reimbursements, fees, funding, or salary from an organisation that may in any way gain or lose financially from the publication of this manuscript, either now or in the future?
- Do you hold any stocks or shares in an organisation that may in any way gain or lose financially from the publication of this manuscript, either now or in the future?
- Do you hold or are you currently applying for any patents relating to the content of the manuscript?
- Have you received reimbursements, fees, funding, or salary from an organization that holds or has applied for patents relating to the content of the manuscript?
- Do you have any other financial competing interests?
- Do you have any non-financial competing interests in relation to this paper?

If you can answer no to all of the above, write 'I declare that I have no competing interests' below. If your reply is yes to any, please give details below.

none

I agree to the open peer review policy of the journal. I understand that my name will be included on my report to the authors and, if the manuscript is accepted for publication, my named report including any attachments I upload will be posted on the website along with the authors' responses. I agree for my report to be made available under an Open Access Creative Commons CC-BY license

(http://creativecommons.org/licenses/by/4.0/). I understand that any comments which I do not wish to be included in my named report can be included as confidential comments to the editors, which will not be published.

Choose an item.

To further support our reviewers, we have joined with Publons, where you can gain additional credit to further highlight your hard work (see: https://publons.com/journal/530/gigascience). On publication of this paper, your review will be automatically added to Publons, you can then choose whether or not to claim your Publons credit. I understand this statement.

Yes Choose an item.
