# Supplementary material for: Genomic data imputation with variational auto-encoders
Source: Gigascience. 2020 Aug 6;9(8):giaa082. doi: 10.1093/gigascience/giaa082 (PMC7407276; doi:10.1093/gigascience/giaa082)
Supplement: giaa082_Reviewer_2_Report_Revision_1 — Natalie Davidson -- 5/27/2020 Reviewed [file giaa082_reviewer_2_report_revision_1.pdf]

Reviewer Report

Title: Genomic data imputation with variational autoencoders

Version: Revision 1 **Date:** 5/27/2020

Reviewer name: Natalie Davidson

Reviewer Comments to Author:

The authors have thoroughly responded to all of my concerns, there are a few minor details remaining.
1) Regarding point 1, "What are the NA values in TCGA data?" I am still not clear how this happened. Is it caused by different reference annotations being used or are these zero values that have been replaced with an NA? After alignment, if they used the same annotation, there should be no NA's, but only counts. It is useful to know exactly where this came from in the pre-processing pipeline.
2)Regarding point 7 "Reading this paper in the context of current genomics research, it may be useful to compare against a model in the wide array of single-cell data imputation models. This is an application where I can see the author's method being applied.":
I still don't fully understand the practical application of your method for RNA-Seq data. When is data missing for bulk RNA-seq data? For microarray I am able to understand it, however in my experience, missing values are not typically seen in bulk RNA-Seq data. In RNA-Seq, one would see counts that are lower than expected in a specific sample due to GC-content biases, or a count of zero when the true count is very low. In practical terms, when I run a bulk RNA-Seq experiment, how would I use your method? Would it be to 1) replace genes with 0 counts with an NA? 2) to replace genes with a lower than expected count with an NA? 3) to be used in panel based sequencing similar to the LINCS L1000?
If 1 or 2, how would be be able to distinguish between an abnormal count and a "true" count? If 3, then you would need to show imputation for a larger amount of missingness
I think if this was further elaborated it would really strengthen the paper as well as give more credence to the percentages missing in your simulation.
3) The authors state "In each missing scenario VAE has a smaller variance than KNN across ten trials (all p values <0.005)." What test was performed?
4) Introduction, first sentence "researches" should be "researchers"

Level of Interest

Please indicate how interesting you found the manuscript: Choose an item.

Quality of Written English

Please indicate the quality of language in the manuscript: Choose an item.

Declaration of Competing Interests

Please complete a declaration of competing interests, considering the following questions:

- Have you in the past five years received reimbursements, fees, funding, or salary from an organisation that may in any way gain or lose financially from the publication of this manuscript, either now or in the future?
- Do you hold any stocks or shares in an organisation that may in any way gain or lose financially from the publication of this manuscript, either now or in the future?
- Do you hold or are you currently applying for any patents relating to the content of the manuscript?
- Have you received reimbursements, fees, funding, or salary from an organization that holds or has applied for patents relating to the content of the manuscript?
- Do you have any other financial competing interests?
- Do you have any non-financial competing interests in relation to this paper?

If you can answer no to all of the above, write 'I declare that I have no competing interests' below. If your reply is yes to any, please give details below.

I declare that I have no competing interests

I agree to the open peer review policy of the journal. I understand that my name will be included on my report to the authors and, if the manuscript is accepted for publication, my named report including any attachments I upload will be posted on the website along with the authors' responses. I agree for my report to be made available under an Open Access Creative Commons CC-BY license (http://creativecommons.org/licenses/by/4.0/). I understand that any comments which I do not wish to be included in my named report can be included as confidential comments to the editors, which will not be published.

Choose an item.

To further support our reviewers, we have joined with Publons, where you can gain additional credit to further highlight your hard work (see: https://publons.com/journal/530/gigascience). On publication of this paper, your review will be automatically added to Publons, you can then choose whether or not to claim your Publons credit. I understand this statement.

Yes Choose an item.
